# Health impacts of parental migration on left-behind children and adolescents: a systematic review and meta-analysis

**DOI:** 10.1016/S0140-6736(18)32558-3

**Published:** 2018-12-15

**Authors:** Gracia Fellmeth, Kelly Rose-Clarke, Chenyue Zhao, Laura K Busert, Yunting Zheng, Alessandro Massazza, Hacer Sonmez, Ben Eder, Alice Blewitt, Wachiraya Lertgrai, Miriam Orcutt, Katharina Ricci, Olaa Mohamed-Ahmed, Rachel Burns, Duleeka Knipe, Sally Hargreaves, Therese Hesketh, Charles Opondo, Delan Devakumar

**Affiliations:** a National Perinatal Epidemiology Unit, University of Oxford, Oxford, UK; b Department of Global Health and Social Medicine, King's College London, London, UK; c Department of Child and Adolescent Psychiatry, New York University School of Medicine, New York, NY, USA; d Great Ormond Street Institute of Child Health, University College London, London, UK; e Department of Clinical, Educational and Health Psychology, University College London, London, UK; f Institute for Global Health, University College London, London, UK; g Department of Social Medicine and Health Education, School of Public Health, Peking University, Beijing, China; h Department of Population Science, University of Bristol, Bristol, UK; i Faculty of Health Sciences, University of Bristol, Bristol, UK; j Faculty of Medicine Siriraj Hospital, Mahidol University, Bangkok, Thailand; k Institute for Medical Information Processing, Biometry and Epidemiology, Ludwig Maximilian University of Munich, Munich, Germany; l Institute for Infection and Immunity, St George's, University of London, London, UK; m International Health Unit, Section of Infectious Diseases and Immunity, Imperial College London, London, UK; n Centre for Global Health, School of Medicine, Zhejiang University, Hangzhou, China; o Department of Medical Statistics, London School of Hygiene and Tropical Medicine, London, UK

## Abstract

**Background:**

Globally, a growing number of children and adolescents are left behind when parents migrate. We investigated the effect of parental migration on the health of left behind-children and adolescents in low-income and middle-income countries (LMICs).

**Methods:**

For this systematic review and meta-analysis we searched MEDLINE, Embase, CINAHL, the Cochrane Library, Web of Science, PsychINFO, Global Index Medicus, Scopus, and Popline from inception to April 27, 2017, without language restrictions, for observational studies investigating the effects of parental migration on nutrition, mental health, unintentional injuries, infectious disease, substance use, unprotected sex, early pregnancy, and abuse in left-behind children (aged 0–19 years) in LMICs. We excluded studies in which less than 50% of participants were aged 0–19 years, the mean or median age of participants was more than 19 years, fewer than 50% of parents had migrated for more than 6 months, or the mean or median duration of migration was less than 6 months. We screened studies using systematic review software and extracted summary estimates from published reports independently. The main outcomes were risk and prevalence of health outcomes, including nutrition (stunting, wasting, underweight, overweight and obesity, low birthweight, and anaemia), mental health (depressive disorder, anxiety disorder, conduct disorders, self-harm, and suicide), unintentional injuries, substance use, abuse, and infectious disease. We calculated pooled risk ratios (RRs) and standardised mean differences (SMDs) using random-effects models. This study is registered with PROSPERO, number CRD42017064871.

**Findings:**

Our search identified 10 284 records, of which 111 studies were included for analysis, including a total of 264 967 children (n=106 167 left-behind children and adolescents; n=158 800 children and adolescents of non-migrant parents). 91 studies were done in China and focused on effects of internal labour migration. Compared with children of non-migrants, left-behind children had increased risk of depression and higher depression scores (RR 1·52 [95% CI 1·27–1·82]; SMD 0·16 [0·10–0·21]), anxiety (RR 1·85 [1·36–2·53]; SMD 0·18 [0·11–0·26]), suicidal ideation (RR 1·70 [1·28–2·26]), conduct disorder (SMD 0·16 [0·04–0·28]), substance use (RR 1·24 [1·00–1·52]), wasting (RR 1·13 [1·02–1·24]) and stunting (RR 1·12 [1·00–1·26]). No differences were identified between left-behind children and children of non-migrants for other nutrition outcomes, unintentional injury, abuse, or diarrhoea. No studies reported outcomes for other infectious diseases, self-harm, unprotected sex, or early pregnancy. Study quality varied across the included studies, with 43% of studies at high or unclear risk of bias across five or more domains.

**Interpretation:**

Parental migration is detrimental to the health of left-behind children and adolescents, with no evidence of any benefit. Policy makers and health-care professionals need to take action to improve the health of these young people.

**Funding:**

Wellcome Trust.

## Introduction

Globally, nearly one in seven individuals are migrants. The majority are labour migrants who originate from low-income or middle-income countries (LMICs) and relocate in search of employment opportunities either internationally or internally within a country (eg, from rural to urban settings).^1^ Some individuals are forced to migrate because of acute drivers such as conflict and disasters. As a result of migration, especially low-skilled labour migration, children are often left behind in the care of other family members or carers. Among labour migrants, a key incentive for migration is the hope of improving the circumstances of their families and children through increased household income and financial stability. International migrants send an estimated US$613 billion per year in remittances to their countries of origin.^2^ Although the health and rights of migrant workers is recognised as a priority in the UN Sustainable Development Goals,^3^ the health of children of migrants has been largely overlooked in research and policy.

**Research in context**

**Evidence before this study**

Migration is increasing globally, which has resulted in a growing number of children and adolescents being left behind when their parents migrate. Before starting this study, we searched the scientific literature for articles on the effect of parental migration on child and adolescent health, and found two narrative reviews: one focused on left-behind children in the Philippines and the other on mental health outcomes in left-behind children in China. These reviews suggested that children benefited from the remittances their parents sent home in terms of improved education and reduced child labour, which could result in improved health, but reported that family separation might have long-term psychological and societal costs. We also identified more than 30 studies, mainly from China, investigating the effects of parental migration on a broad range of health outcomes across different countries. On Nov 26, 2018, we did an updated search of MEDLINE for systematic reviews with no date or language restrictions, using the broad search terms “(child* OR adolescent) AND health AND (migration OR left-behind)”. Although we identified 99 systematic reviews, none reviewed the key areas of health of left-behind children and adolescents across all low-income and middle-income countries (LMICs).

**Added value of this study**

This is the largest and most comprehensive study to date assessing the impact of parental migration on all key areas of child and adolescent health across all LMICs. Compared with children of non-migrants, left-behind children and adolescents had an increased risk of depression, suicidal ideation, and risk of anxiety. Left-behind children also had smaller increases in risk for wasting, stunting, and substance use. These results highlight a rarely discussed consequence of global migration with implications for global policy making and health-care provision in migrant-sending countries. Although a small number of individual studies found positive health effects of parental migration, overall we found no evidence of benefit across any of the health outcomes.

**Implications of all the available evidence**

Our findings highlight the unmet health needs of left-behind children and adolescents. Research to date has focused primarily on China and longitudinal studies in a wider range of LMICs with high rates of emigration are needed to better understand risk and resilience factors within this population, and to inform policy and practice to address unmet health needs in left-behind children, adolescents, and their carers.

No estimates are available for the number of left-behind children and adolescents globally, but the figure is thought to be in the hundreds of millions. More than a third of all children residing in rural China (61 million) are left behind by one or both migrant parents.^4^ 27% of children in the Philippines,^5^ 36% in Ecuador,^6^ and more than 40% in rural South Africa^7^ are estimated to be left behind.

Evidence about the health status of left-behind children is conflicting. On the one hand, material benefits and greater income security from remittances might be expected to confer improvements in health and facilitate access to health care and education. In Pakistan, migration had positive effects on the growth of left-behind children, with girls benefitting more than boys.^8^ However, on the other hand, some studies suggest poorer health outcomes among left-behind children. In China, where the most research has been done to date, studies have shown poorer nutritional,^9^ developmental^10^ and mental health outcomes^11^ in left-behind children than children of non-migrant parents. It is unclear to what extent the health of these children is affected by parental migration, and how the impact might vary according to contextual factors, including sex and age. For example, in China, boys who were left before age 6 years were not as tall as boys whose parents left them at an older age.^12^ Although adolescents might be more independent than younger children, parental absence, and lack of supervision at this crucial age has been associated with risk-taking behaviours, including substance use and physical inactivity, with implications for long-term health.^13^ Furthermore, effects might vary according to the circumstances of parental migration. For example, maternal absence and the absence of both parents might have more pronounced effects on children's health than paternal absence alone.^14^ To date, to our knowledge, no studies have comprehensively examined the health status of left-behind children and adolescents across all settings and key areas of health. To address this research gap, we did a systematic review and meta-analysis to assess the impact of parental migration on child and adolescent nutrition, mental health, unintentional injuries, infectious disease, substance use, unprotected sex, early pregnancy, and verbal, physical, and sexual abuse in LMICs. We investigated whether parental migration status (one or both parents migrating), type of migration (internal or international; labour or forced) and child characteristics (age, sex) differentially influence the health of left-behind children and adolescents.

## Methods

### Search strategy and selection criteria

For this systematic review and meta-analysis, we searched MEDLINE, Embase, CINAHL, the Cochrane Library, Web of Science, PsychINFO, Global Index Medicus, Scopus, and Popline from database inception to April 27, 2017. Full search terms are provided in the appendix. We searched for observational studies reporting the risk of health outcomes done in LMICs (classified according to the World Bank classification) that included children and adolescents (aged 0–19 years) with at least one migrant parent. We defined parental migration as one or more parent moving away from the place their children are living, for a minimum of 6 months. We included studies in which parents had migrated for any reason, such as employment (labour migrants) or armed conflict or disasters (forced migrants). We included internal and international parental migration, defined as migration within and beyond a country's borders, respectively. The comparator group was children of non-migrating parents. We excluded studies in which less than 50% of participants were aged 0–19 years, the mean or median age of participants was more than 19 years, fewer than 50% of parents had migrated for more than 6 months, or the mean or median duration of migration was less than 6 months.

We updated our searches to include all literature published before Sept 5, 2018, to assess whether studies published after our original search might alter the implications of our findings. We tailored search strategies to each database and used controlled vocabulary and search filters where available, or Boolean search methods and free text terms. No date or language restrictions were used. Because of the large volume of research on left-behind children available in China, we searched the China National Knowledge Infrastructure database and key Chinese public health journals. We also searched reference lists of relevant systematic reviews and grey literature published by key international organisations (UN Children's Fund, International Organization for Migration, and UN High Commissioner for Refugees). The full search strategy is detailed in the appendix. We used Covidence systematic review software (Veritas Health Innovation, Melbourne, VIC, Australia) to organise and screen articles. Two reviewers (KR-C, GF, CZ, LKB, YZ, HS, BE, AB, WL, MO, DK, or DD) independently screened each title and abstract and excluded those that were not relevant, and then independently screened the full text of remaining studies to assess eligibility. Two reviewers (GF, CZ, LKB, YZ, AM, HS, BE, RB, WL, MO, KR, or OM-A) extracted data, and assessed the risk of bias for all included studies. Discrepancies about study inclusion were resolved through discussion with a third reviewer or by contacting study authors. Studies that reported results as mean scores with SDs or as raw proportions or unadjusted odds ratios (ORs) were included in meta-analysis. When insufficient data were reported for inclusion in the meta-analysis, we contacted study authors to request further information.

This study is reported in accordance with the PRISMA guidelines^15^ (appendix). The study protocol is available online.

### Data analysis

We extracted data on study design, participant numbers and characteristics, and exposures and outcomes using data extraction sheets designed by the authors (appendix). When duplicate data were identified, only data for the most recent timepoint were extracted.

Outcomes of interest were risk and prevalence of the main causes of disability-adjusted life-years for children aged younger than 5 years, 5–9 years, and 10–19 years, including nutrition (stunting, wasting, underweight, overweight and obesity, low birthweight, and anaemia), mental health (depressive disorder, anxiety disorder, conduct disorders, self-harm, and suicide), unintentional injuries, substance use, physical, emotional, and sexual abuse,^16^ and infectious disease. Additional outcomes were unprotected sex and early pregnancy (<18 years; appendix).

We summarised outcomes from all studies included in the review diagrammatically, using adjusted estimates to classify studies according to their effect estimates and provide a visual overview of the evidence. Studies with sufficient data to examine the effect of being left behind on nutrition, mental health, injury, and substance use outcomes were included in random-effects meta-analysis. We estimated pooled risk ratios (RRs) with 95% CIs for binary outcomes and standardised mean differences (SMDs) with 95% CI for continuous outcomes. Binary categorisations indicate the presence or absence of a disorder (caseness), and continuous outcomes were associated with symptom severity. We used unadjusted study outcomes for three main reasons. First, only 15 studies reported adjusted effect estimates. Second, the adjusted effects estimates varied considerably with regard to the covariates included and effects were not directly comparable. Third, a number of studies reported adjusted ORs; due to the so-called non-collapsibility property of ORs,^17^ estimates from adjusted ORs can differ significantly from unadjusted estimates even in the absence of confounding. We used meta-regression to assess the effect of child age and sex on study-specific effect estimates.

Risk of bias was assessed using an adapted version of the Newcastle Ottawa Scale^18^ incorporating items from the National Institute for Clinical Excellence Quality Appraisal^19^ (appendix). Studies with a high or unclear risk of bias across five or more domains were defined as being at high risk of bias. This definition was based on consensus between the authors, which acknowledged that any such cutoffs are arbitrary.^20^ No studies were excluded on the basis of quality. Funnel plots were used to assess publication bias.

We used the *I*^2^ statistic to indicate the proportion of total variation between study estimates due to heterogeneity.^20^ To identify and assess sources of heterogeneity, we planned a-priori subgroup analyses to assess migration of one versus both parents, and forced versus labour migration. We also planned to do subgroup analyses of internal versus international migration, but due to the predominance of Chinese studies, which were all on internal migration, we decided to do subgroup analyses of studies in China versus the rest of the world. We did a sensitivity analysis to assess the robustness of our conclusions with regard to the assumptions underlying our analytic approach. We did fixed-effects meta-analyses and repeated analyses using only studies with low risk of bias. All statistical analyses were done using Stata (version 13.0) and MetaXL (version 5.3). The study is registered with PROSPERO, number CRD42017064871.

### Role of the funding source

The funder had no role in study design, data collection, data analysis, data interpretation, writing of the report, or the decision to submit. The corresponding author had full access to all the data in the study and had final responsibility for the decision to submit for publication.

## Results

**Figure 1:**
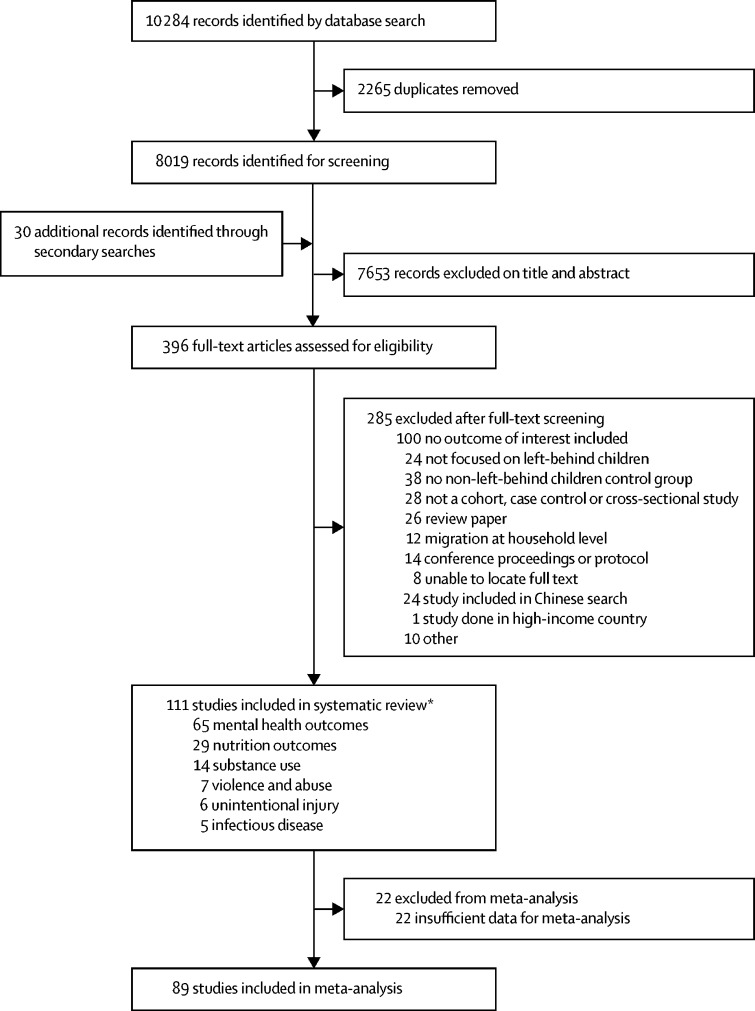
Study selection *Some studies included more than one outcome.

**Figure 2:**
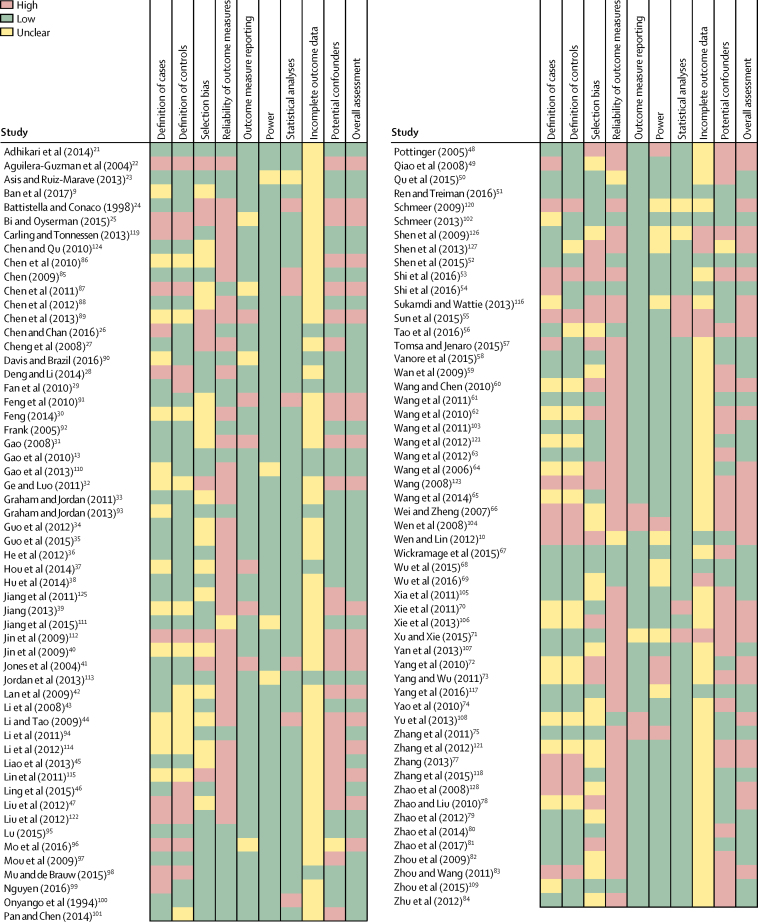
Quality assessment of studies included in the systematic review Scoring was based on an adapted version of the Newcastle Ottawa Scale^18^ incorporating items from the National Institute for Clinical Excellence Quality Appraisal. Studies with a high or unclear risk of bias across five or more domains were defined as being at high risk of bias overall.

Our systematic search of the literature identified 10 284 records, of which 2265 were duplicates (figure 1). Of the 396 full-text articles retrieved, 111 studies^9^, ^10^, ^13^, ^21^, ^22^, ^23^, ^24^, ^25^, ^26^, ^27^, ^28^, ^29^, ^30^, ^31^, ^32^, ^33^, ^34^, ^35^, ^36^, ^37^, ^38^, ^39^, ^40^, ^41^, ^42^, ^43^, ^44^, ^45^, ^46^, ^47^, ^48^, ^49^, ^50^, ^51^, ^52^, ^53^, ^54^, ^55^, ^56^, ^57^, ^58^, ^59^, ^60^, ^61^, ^62^, ^63^, ^64^, ^65^, ^66^, ^67^, ^68^, ^69^, ^70^, ^71^, ^72^, ^73^, ^74^, ^75^, ^76^, ^77^, ^78^, ^79^, ^80^, ^81^, ^82^, ^83^, ^84^, ^85^, ^86^, ^87^, ^88^, ^89^, ^90^, ^91^, ^92^, ^93^, ^94^, ^95^, ^96^, ^97^, ^98^, ^99^, ^100^, ^101^, ^102^, ^103^, ^104^, ^105^, ^106^, ^107^, ^108^, ^109^, ^110^, ^111^, ^112^, ^113^, ^114^, ^115^, ^116^, ^117^, ^118^, ^119^, ^120^, ^121^, ^122^, ^123^, ^124^, ^125^, ^126^, ^127^, ^128^ done between 1994 and 2017 in 16 countries (n=106 167 left-behind children and adolescents; n=158 800 children and adolescents of non-migrant parents) were included in the systematic review (appendix). 89 studies (n=78 273 left-behind children and adolescents; n=88 350 children of non-migrant parents) were included in meta-analyses. Reasons for exclusion at the full-text screening stage and characteristics of included studies are shown in the appendix. Of the 111 included studies, 91 (82%) were done in China, 58 (52%) were published in Chinese, nine (8%) were done in Asia (Thailand, Philippines, Indonesia, Vietnam, Sri Lanka, and India), six (5%) in Latin America (Mexico, Guatemala, and Peru), three (3%) in Africa (Ethiopia, Kenya, and Malawi), two (2%) in the Caribbean (Trinidad and Tobago and Jamaica), and two (2%) in eastern Europe (Romania and Moldova). 101 studies were cross-sectional, seven were cohort studies, and three were case-control studies. All studies included children of labour migrants; none included children of forced migrants. All Chinese studies examined internal migration within China, whereas studies from the rest of the world, with the exception of one study,^100^ focused on international migration. 71 studies included children aged younger than 10 years. Among the 92 studies that reported participant sex, the proportion of male participants ranged from 13·1% to 76·3%. Study quality varied by domain assessed (figure 2). 48 (43%) of the 111 included studies were at high or unclear risk of bias across five or more domains. Funnel plots showed no evidence of publication bias (appendix).

**Figure 3:**
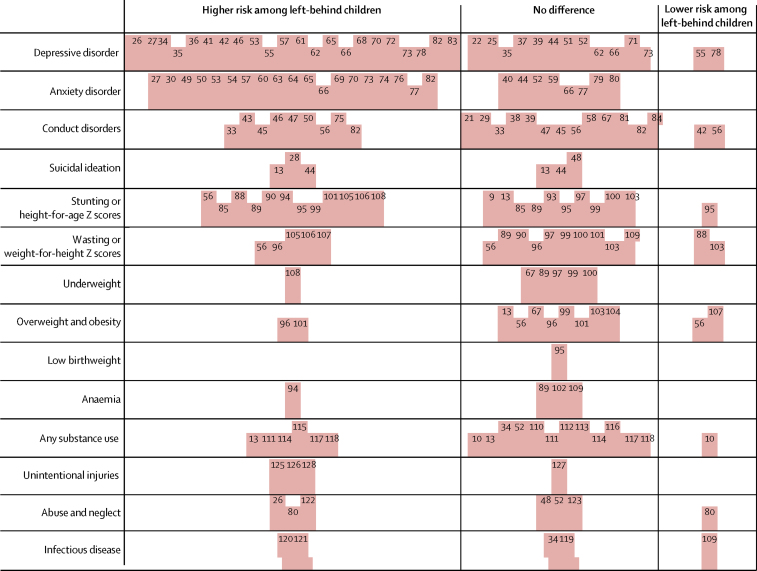
Harvest plot of health outcomes among left-behind children and children of non-migrant parents included in the systematic review Each full-height bar represents the health outcomes reported by an individual study included in the systematic review. Half-height bars represent studies which found varying directionality of health outcomes between different population subgroups (eg, a higher risk among girls who were left behind but no difference among boys who were left behind compared with children of non-migrant parents). Numbers refer to study references as cited in the reference list. Nine studies^23^, ^24^, ^31^, ^32^, ^86^, ^87^, ^91^, ^98^, ^124^ were excluded from this plot due to the absence of significance testing reported in these studies. Two studies^105^, ^109^ were included for those outcomes for which significance testing was reported and excluded for other outcomes.

Of the 111 studies included in the systematic review, mental disorders were the most common study outcome (n=64), followed by nutritional status (n=29), substance use (n=14), experience of violence and abuse (n=7), unintentional injury (n=6), and infectious disease (n=5; figure 3). Across all outcomes, only 12 studies reported a lower risk of adverse health outcomes among left-behind children and adolescents.

**Figure 4:**
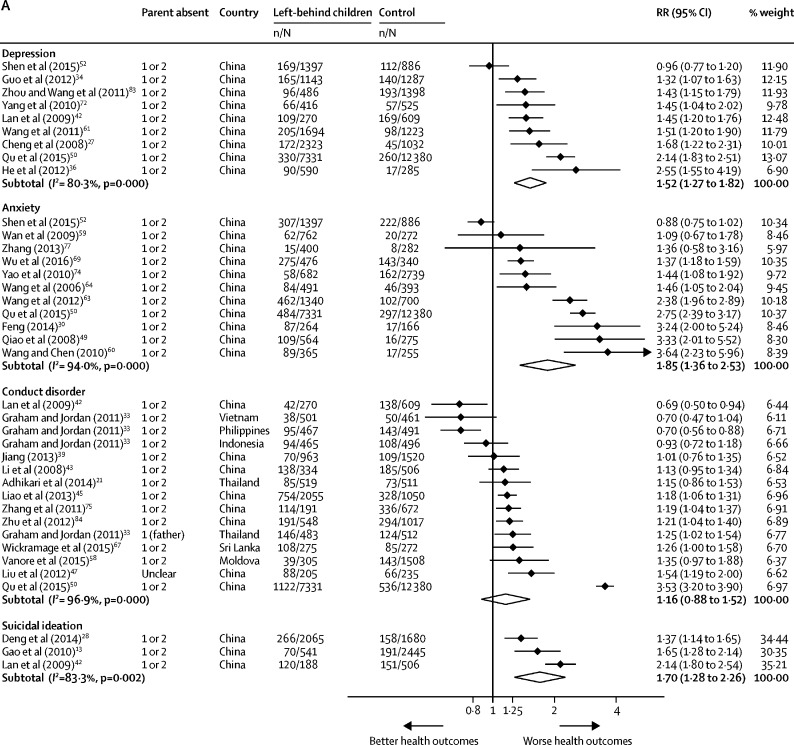

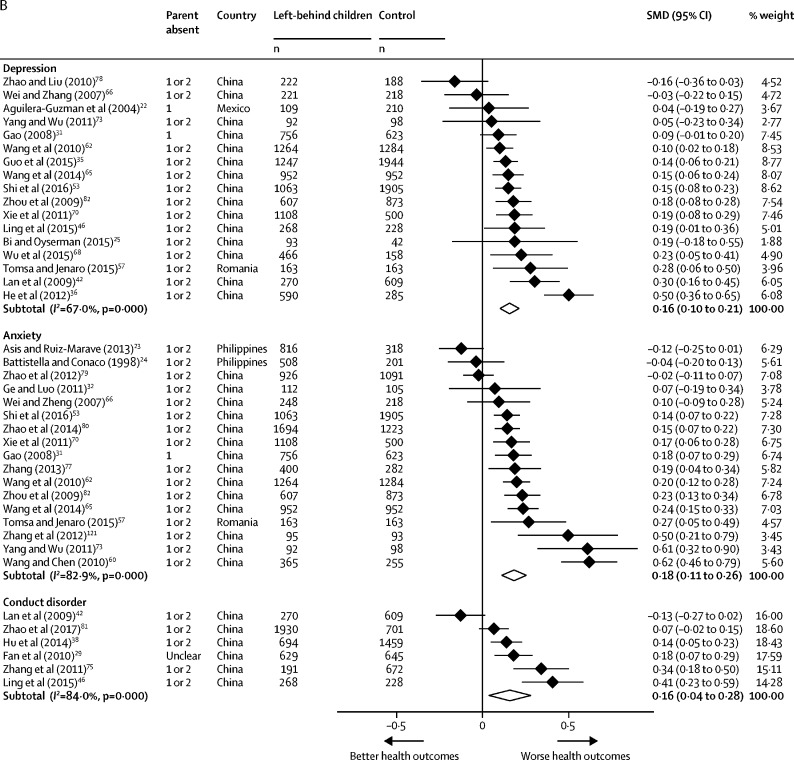

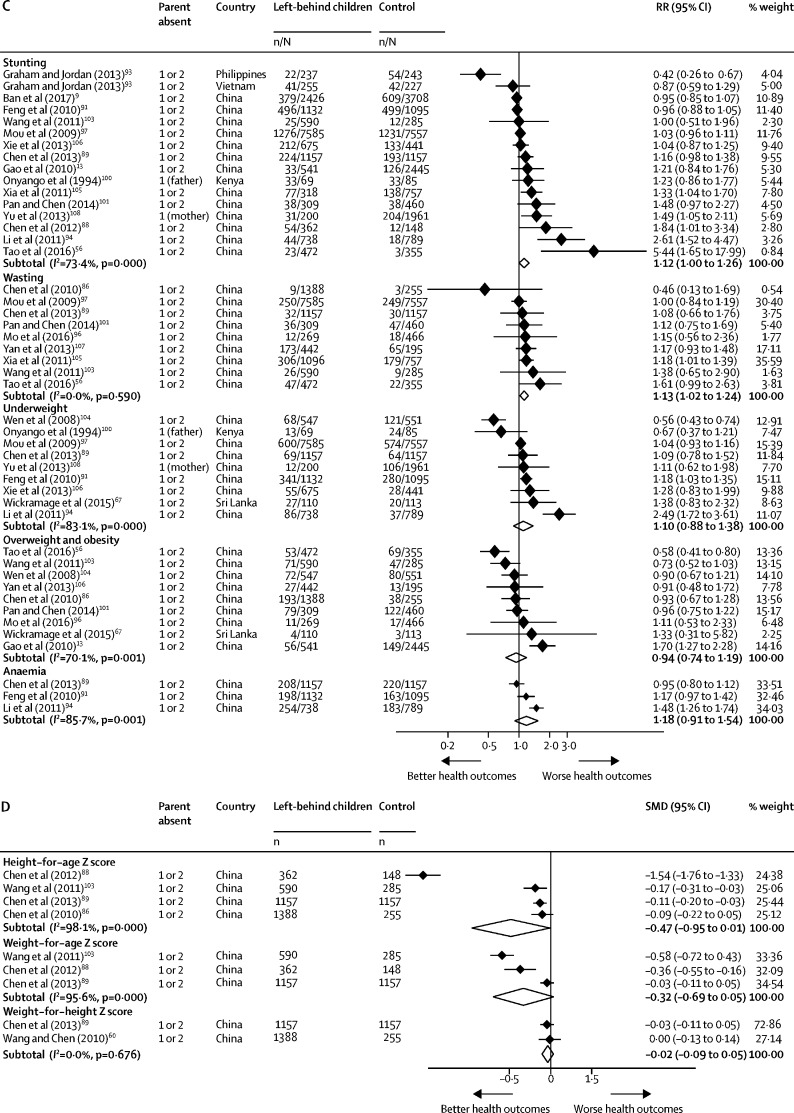

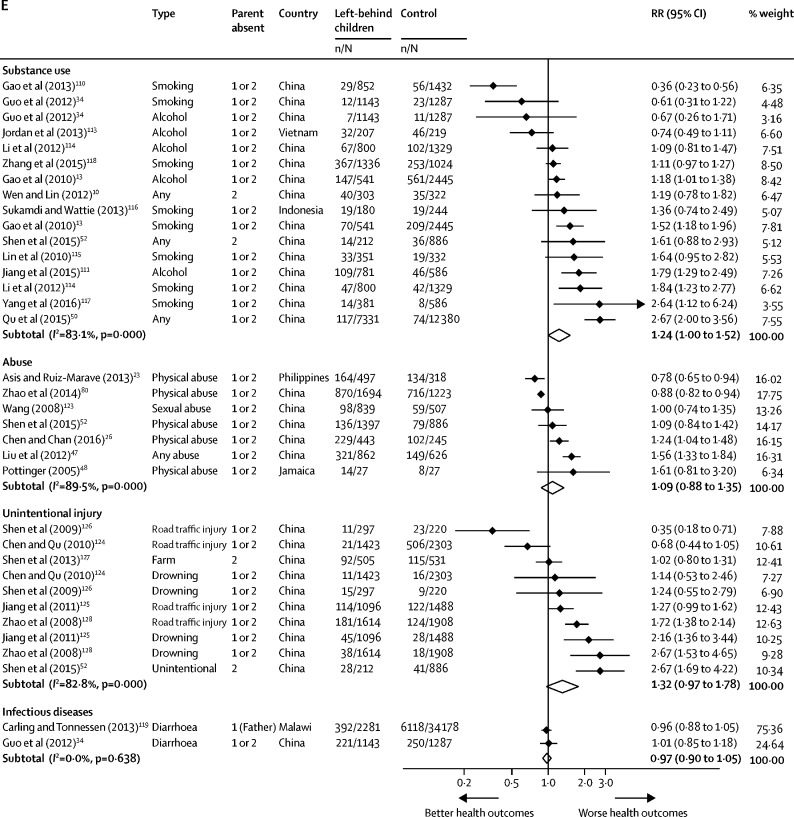
Forest plots of relative risks or standardised mean differences for health outcomes Data are presented for mental health binary outcomes (A), mental health continuous outcomes (B), nutrition binary outcomes (C), nutrition continuous outcomes (D), and substance use, abuse and injury outcomes (E). Weights were assigned by random effects analysis. RR=relative risk. SMD=standardised mean difference.

64 of 65 studies reporting mental health outcomes used self-reported screening tools. Meta-analysis showed that left-behind children and adolescents had a significantly higher risk of depression caseness (RR 1·52 [95% CI 1·27–1·82]) and symptoms (SMD 0·16 [95% CI 0·10–0·21]), anxiety caseness (RR 1·85 [1·36–2·53]) and symptoms (SMD 0·18 [0·11–0·26]), and suicidal ideation caseness (RR 1·70 [1·28–2·26]) compared with children of non-migrating parents (figures 4A, figure 4B). Left-behind children and adolescents had a higher risk of symptoms of conduct disorder (SMD 0·16 [95% CI 0·04–0·28]) but not caseness (RR 1·16 [95% CI 0·88–1·52]). Statistical heterogeneity across mental disorder outcomes was high (*I*^2^=67·0–96·9%). In subgroup analyses, no differences were identified in risk of anxiety caseness or symptoms among children and adolescents left behind by one parent or by both parents compared with children and adolescents of non-migrant parents (appendix). Children and adolescents left behind by both parents had a higher risk of depressive symptoms than did those of non-migrants (SMD 0·11 [95% CI 0·01 to 0·22]), but no differences were identified between children or adolescents left behind by one parent compared with children or adolescents of non-migrants (0·04 [–0·03 to 0·12]; appendix). No significant differences in depression caseness were identified between children and adolescents left behind by one (RR 0·93 [95% CI 0·74–1·17]) or by both parents (1·13 [0·78–1·64]) when compared with children and adolescents of non-migrant parents (appendix). In studies outside of China, no significant differences in risk of mental disorders were found among children and adolescents of international migrants compared with children of non-migrant parents. With the exception of conduct disorders, the number of studies of children of international migrants done outside of China was limited. Among children of migrants in China (all of whom were internal migrants) risks for all health outcomes were consistent with the results of the main analyses (appendix). Excluding studies at high risk of bias did not alter mental disorder outcomes. Using a fixed-effects model did not significantly alter effect estimates with the exception of conduct disorder caseness (RR 1·45 [95% CI 1·38–1·52]; appendix). We did not include any studies in the meta-analysis reporting outcomes for self-harm.

Meta-analyses showed that left-behind children had a significantly increased risk of wasting (RR 1·13 [95% CI 1·02–1·24]) and stunting (RR 1·12 [1·00–1·26]) than children of non-migrants. No differences were identified in mean height-for-age Z score (SMD −0·47 [95% CI −0·95 to 0·01]), mean weight-for-height Z score (SMD −0·02 [–0·09 to 0·05]), mean weight-for-age Z score (SMD −0·32 [–0·69 to 0·05]), risk of being underweight (RR 1·10 [0·88 to 1·38]), risk of being overweight or obese (RR 0·94 [0·74 to 1·19]), or risk of having iron-deficiency anaemia (RR 1·18 [0·91 to 1·54]) between left-behind children and children of non-migrating parents (figure 4C, figure 4D). Heterogeneity varied across nutrition outcomes (*I*^2^=0·0–98·1%), with all outcomes (with the exception of wasting and weight-for-height Z score) varying substantially between studies.

Subgroup analyses of wasting for children left behind by one parent and by both parents showed no statistical evidence of an increased risk in left-behind children compared with children of non-migrants (appendix). Subgroup analyses of stunting revealed a significant increase in risk for children left behind by one parent (RR 1·28 [95% CI 1·06–1·55]) and children left behind by both parents (1·26 [1·06–1·50]; appendix) compared with children of non-migrating parents. Among studies done in China, children left behind had a higher risk of stunting than children of non-migrants, but all other nutrition outcomes remained unchanged. Of the studies done outside of China, only three^67^, ^93^, ^100^ reported nutrition outcomes for children who were left behind. Overall no difference was found in nutrition outcomes in studies outside of China, with the exception of wasting and weight-for-height Z scores (appendix). Excluding studies at high risk of bias did not alter nutrition outcomes. Using a fixed-effects model resulted in significantly worse height-for-age Z scores (SMD −0·23 [95% CI −0·29 to −0·17]) and weight-for-age Z scores (−0·19 [–0·25 to −0·12]) among left-behind children than children of non-migrant parents; all other outcomes remained unchanged (appendix).

Left-behind children had a marginally higher risk of substance use (RR 1·24 [95% CI 1·00–1·52]) including alcohol, smoking, and any substance use. No significant differences were identified in risk of unintentional injury (RR 1·32 [95% CI 0·97–1·78]), abuse (RR 1·09 [0·88–1·35]), or diarrhoea (RR 0·97 [0·90–1·05]; figure 4E) between left-behind children and children of non-migrant parents. Statistical heterogeneity was high across these outcomes (*I*^2^=82·8–83·1%). Pooled risk for substance use outcomes in studies outside of China and among children left behind by one parent showed no increased risk compared with children of non-migrants; however, each subgroup included only two studies (appendix). When studies at high risk of bias were excluded, no significant differences were identified in risk of substance use among left-behind children compared with children of non-migrant parents. Fixed effects meta-analysis revealed a higher risk of unintentional injury among left-behind children than children of non-migrant parents (RR 1·35 [95% CI 1·21–1·52]; appendix). No studies included in the meta-analysis reported outcomes for infectious disease, with the exception of diarrhoea, or outcomes for unprotected sex or early pregnancy.

Meta-regression showed sex and mean age had no significant effects on any health outcomes (appendix).

## Discussion

Although most studies identified by our systematic review focused on internal labour migration in China, our findings suggest that, as a group, left-behind children and adolescents have worse outcomes than children of non-migrant parents, especially with regard to mental health and nutrition. Compared with children of non-migrants, left-behind children and adolescents had a 52% increased risk of depression, 70% increased risk of suicidal ideation, and an 85% increased risk of anxiety. We found smaller increases in risk for wasting (13%), stunting (12%) and substance use (24%). Left-behind children and adolescents had no increased risk of conduct disorders, being overweight or obese, anaemia, unintentional injury, diarrhoea, or abuse. Although a minority of individual studies^10^, ^42^, ^55^, ^56^, ^78^, ^80^, ^88^, ^95^, ^103^, ^107^, ^109^ reported beneficial health effects, no overall benefits were found across any of the outcomes assessed. We found no studies investigating the effect of forced migration, which might be explained by the fact that leaving children behind in the context of conflict or disaster is unlikely.

We updated our search from April 28, 2017, to Sept 5, 2018, using the same search terms and databases. This updated search identified nine additional papers (six published in English and three published in Chinese), all of which focused on internal migration and were done in China. Findings from the studies were consistent with the results from our meta-analyses and provide no evidence of benefits of parental migration for left-behind children and adolescents and support our overall findings in terms of the negative health impact of migration on left-behind children and adolescents. Four studies reported outcomes for depression: three studies^129^, ^130^, ^131^ found a small increase in depressive symptoms or worse depression scores among left-behind children and adolescents and one study^132^ found no difference between left-behind children and children of non-migrant parents. Studies^129^, ^133^ reporting anxiety outcomes similarly found increased risks among left-behind children and adolescents compared with children of non-migrants. Consistent with our findings, a large study of adolescents in China (n=13 952) found a significant increase in suicide attempts in left-behind adolescents compared with adolescents who were not left behind (3·75% *vs* 2·86%, p<0·01).^134^ Two studies^135^, ^136^ assessed nutrition outcomes. A cohort study^135^ found that left-behind children and adolescents had lower weight-for-age Z scores and height-for-age Z scores at baseline and follow-up after migration, although the effect of migration varied according to which parent migrated. Li and Zhang^136^ found that a higher proportion of left-behind children had stunting and wasting than did children with non-migrant parents. One study^137^ reported a higher risk of any type of unintentional injury (eg, vehicle and traffic injuries and falls) among left-behind children and adolescents than children and adolescents with non-migrant parents (adjusted OR 1·208, p<0·05).

Labour migration is a global trend, shaping families and communities across the world.^138^ Our findings are consistent with previous reviews^5^, ^11^ about left-behind children in rural China and the Philippines: although parental labour migration might have economic benefits for families, it might have hidden costs for the health of children and adolescents who are left behind. A previous study^139^ reported that these negative health consequences extend to other family members. The Child Health and Migrant Parents in South-East Asia study^139^ showed that left-behind mothers and other carers in transnational migrant households were more likely to have poor mental health than carers in non-migrant households: mental health problems were associated with infrequent contact with the migrant and migrant destinations in the Middle East, whereas receiving remittances in the past 6 months was found to have a protective effect on the mental health of carers. With the exception of age and sex, we were unable to investigate factors mediating poor health outcomes among left-behind children and adolescents, although family structure, community social capital, living conditions, and level of caregiver supervision might be important.^68^, ^140^ Future research should consider the circumstances of parental migration. Children of parents migrating because of extreme poverty, disasters, or oppression are likely to have worse health outcomes than children from wealthier migrant families that are financially stable with access to adequate health care. Residing with siblings and relationships between children and their caregivers could also be important.

82% of studies included in our systematic review were done in China, an upper middle-income country where migration is mainly internal, rural-to-urban labour migration. Our study highlights a major research gap in countries beyond China, potentially limiting the generalisability of our findings to other forms of migration and to other settings, especially low-income countries. Subgroup analyses of studies done across the rest of the world showed no difference in outcomes for left-behind children and adolescents; however, with the exception of conduct disorder, few studies have been done, limiting the conclusions that can be drawn.

Addressing the needs of families who are left behind will be essential for health-care workers and policy makers.^141^ In China, the health and wellbeing of left-behind children is a priority and steps are being taken to address this. In 2013, the Chinese Government called on local authorities to take specific responsibility for the education and care of left-behind children.^142^ The Chinese Women's Federation has taken a lead in most provinces, but action has been inconsistent and it is unclear whether the health of left-behind children is improving as a consequence. Community-based clubs for children have been established to provide left-behind children with educational and recreational opportunities.^143^ Other strategies include conditional cash transfer schemes for caregivers to encourage them to attend health education sessions, vaccinations, and health checks.^144^ In China until around 10 years ago, the national household registration system limited rural children's access to urban health and educational services, with children forced to attend designated migrant schools, which varied in quality. The situation is now changing, especially in smaller cities, as a result of the national household registration system restrictions being relaxed, migrant children attending mainstream schools, and using rural health insurance to access health care. These changes have led to an increase in the number of children migrating with their parents.^145^

Next steps for research and practice require a multifaceted approach, involving clinical, epidemiological, intervention research, and policy perspectives (panel). Focusing on all levels of society, the International Organization for Migration recommends a multi-dimensional intervention framework that includes the government and business.^146^ Clinicians, teachers, and other individuals working with left-behind children and adolescents must be aware of the potential mental health and nutritional needs of this population, and be trained to support and treat them. Increased awareness is particularly important with regard to common mental disorders and risk-taking behaviours that children or adolescents might not present with, or that might be underlying another clinical presentation. Global mental health initiatives should be encouraged to incorporate a focus on left-behind children. However, a one-size-fits-all approach to intervention is likely to be ineffective since left-behind children and adolescents will have different experiences of migration and being left behind. A study in China^68^ found that children who were left behind had more depressive symptoms than children residing in rural China with both parents who had never migrated or been left behind, regardless of whether they had previously migrated or not. However, children who were previously left-behind but were now living with their parents had fewer depressive symptoms than children in rural areas without any experience of migration or being left behind.^68^ Although sex was not a predictor of health outcomes among left-behind children and adolescents in our study, girls and boys might require different intervention approaches. Interventions are also needed to support caregivers, many of whom might be elderly relatives with health needs of their own. Increasing the evidence base beyond China is essential, as are longitudinal studies investigating the long-term effects of parental migration on children and adolescents. Although familial separation is acutely detrimental for health, children might go on to develop resilience and have potentially better health outcomes.

Panel **Next steps and future research**

**Clinical**

• Health-care providers should have an increased awareness with regard to mental health and nutritional disorders when dealing with left-behind children and adolescents. More focus should be placed on research to better understand the health needs of this group in a range of countries globally.

**Epidemiology**

• Increase in the evidence base and available data to understand the short-term and long-term health consequences of migration on left-behind children, with a particular focus on internal migration outside of China and international migration, elucidating the mechanisms by which being left behind might lead to improvements or worsening health. To do this, more work is needed on the moderating and mediating factors—for example, the number of parents migrating, the type and duration of migration, the degree of contact with parents, or age of the children and the differing family situations, including alternate family structures.

**Intervention research**

• Moving beyond understanding the problem, interventions are needed to improve the health or to mitigate the adverse effects of being left behind. This might involve community actions, laws, or technology to improve the connectedness of families. Research is particularly needed on interventions at an individual or community level to promote resilience and enable young people to overcome the negative aspects of parental absence due to migration.

**Policy**

• Both global and national policies need to consider the health needs of children who are left behind. Research is needed to identify and implement national policies to provide services for children who might not have parental support. Globally, policies for migrant workers should consider the impact on their families. Migrant workers must be allowed the time to visit and communicate with their families. Global mental health initiatives need to better consider this excluded group.

The comprehensive scope of this review is a strength, since evidence was included across all LMICs, in all languages, across multiple health outcomes, with low publication bias. However, our study has several limitations. Our original systematic search included literature published up to April, 2017, and thus newer studies might alter the conclusions. However, when updating the searches to September, 2018, the studies were consistent with our findings. Statistical heterogeneity was high in the meta-analyses, which persisted in subgroup analyses and meta-regression. This heterogeneity suggests that, despite our use of a strict definition of left-behind children and adolescents, other mediating or moderating factors might influence the results reported in individual studies, including caregiver and contextual factors. Similarly high heterogeneity was identified in a systematic review and meta-analysis^147^ of mental disorders among refugees resettled in western countries. Most of the studies included in our systematic review and meta-analysis were from China, focused on internal migration, and were cross-sectional, which means temporal causal inference is limited and might not generalise beyond China. Despite these limitations, our study defines and identifies a global population of young people at risk.

In summary, left-behind children and adolescents have substantial unmet mental health and nutritional needs that have not been well described outside of China. The prevalence of labour migration is increasing, thus interventions that support these young people are urgently needed to prevent long-term negative effects on their health and development.
