# Supplementary material for: Health impacts of parental migration on left-behind children and adolescents: a systematic review and meta-analysis
Source: Lancet. 2018 Dec 15;392(10164):2567–82. doi: 10.1016/S0140-6736(18)32558-3 (PMC6294734; doi:10.1016/S0140-6736(18)32558-3)
Supplement: Supplementary appendix [file mmc1.pdf]

THE LANCET

Supplementary appendix

This appendix formed part of the original submission and has been peer reviewed. We post it as supplied by the authors.

Supplement to: Fellmeth G, Rose-Clarke K, Zhao C, et al. Health impacts of parental migration on left-behind children and adolescents: a systematic review and meta-analysis. *Lancet* 2018; published online Dec 5. http://dx.doi.org/10.1016/S0140-6736(18)32558-3.

Health impacts of parental migration on left-behind children and adolescents: a systematic review and meta-analysis

Gracia FELLMETH[1]*, Kelly ROSE-CLARKE[2]*, Chenyue ZHAO[3], Laura K BUSERT[4], Yunting ZHENG[5], Alessandro MASSAZZA[6], Hacer SONMEZ[7], Ben EDER[8], Alice BLEWITT[8], Wachiraya LERTGRAI[9], Miriam ORCUTT[8], Katharina RICCI[10], Olaa MOHAMED-AHMED[1], Rachel BURNS[8], Duleeka KNIPE[11], Sally HARGREAVES[12,13], Professor Therese HESKETH[8,14], Charles OPONDO[1,15], Delan DEVAKUMAR[8]
*Contributed equally as joint first authors

[1] National Perinatal Epidemiology Unit, University of Oxford, Oxford, UK (Gracia Fellmeth MSc; Olaa Mohamed-Ahmed MSc; Charles Opondo PhD;)
[2] Department of Global Health and Social Medicine, King's College London (Kelly Rose-Clarke MBPhD)
[3] Department of Child and Adolescent Psychiatry, New York University School of Medicine (Chenyue Zhao PhD)
[4] Great Ormond Street Institute of Child Health, University College London (Laura K Busert, MSc)
[5] Department of Social Medicine and Health Education, School of Public Health, Peking University (Yunting Zheng MSc)
[6] Department of Clinical, Educational and Health Psychology, University College London (Alessandro Massazza MSc)
[7] Faculty of Health Sciences, University of Bristol (Hacer Sonmez BSc)
[8] Institute for Global Health, University College London (Ben Eder MBChB; Alice R Blewitt; Miriam Orcutt MBBS; Rachel Burns MSc; Therese Hesketh PhD; Delan Devakumar PhD)
[9] Faculty of Medicine Siriraj Hospital, Mahidol University (Wachiraya Lertgrai MSc)
[10] Institute for Medical Information Processing, Biometry and Epidemiology, Ludwig-Maximilian University Munich (Katharina Ricci BA Hons)
[11] Department of Population Science, Faculty of Health Sciences, University of Bristol (Duleeka Knipe PhD)
[12] Institute for Infection and Immunity, St George's, University of London, London (Sally Hargreaves FRCPE)
[13] International Health Unit, Section of Infectious Diseases and Immunity, Imperial College London, London (Sally Hargreaves FRCPE)
[14] Centre for Global Health, Zhejiang University School of Medicine (Therese Hesketh PhD)
[15] Department of Medical Statistics, London School of Hygiene & Tropical Medicine, London, UK

Contents

Preferred reporting items for systematic reviews and meta-analysis (PRISMA) guidelines	2
Inclusion criteria	5
Full search strategy	6
Data extraction including risk of bias	8
Excluded studies	17
Tables of included studies	18
Table of study characteristics: substance use	53
Table of excluded studies	62
Funnel plots	107
Subgroup analysis	111
Sensitivity analysis	122
Meta-regression	131
References (same numbering as the main text)	132

Preferred reporting items for systematic reviews and meta-analysis (PRISMA) guidelines

Section/topic	#	Checklist item	Reported on page #
**TITLE**			
Title	1	Identify the report as a systematic review, meta-analysis, or both.	1
**ABSTRACT**			
Structured summary	2	Provide a structured summary including, as applicable: background; objectives; data sources; study eligibility criteria, participants, and interventions; study appraisal and synthesis methods; results; limitations; conclusions and implications of key findings; systematic review registration number.	1
**INTRODUCTION**			
Rationale	3	Describe the rationale for the review in the context of what is already known.	1
Objectives	4	Provide an explicit statement of questions being addressed with reference to participants, interventions, comparisons, outcomes, and study design (PICOS).	1
**METHODS**			
Protocol and registration	5	Indicate if a review protocol exists, if and where it can be accessed (e.g., Web address), and, if available, provide registration information including registration number.	1, 3
Eligibility criteria	6	Specify study characteristics (e.g., PICOS, length of follow-up) and report characteristics (e.g., years considered, language, publication status) used as criteria for eligibility, giving rationale.	3
Information sources	7	Describe all information sources (e.g., databases with dates of coverage, contact with study authors to identify additional studies) in the search and date last searched.	2, 3
Search	8	Present full electronic search strategy for at least one database, including any limits used, such that it could be repeated.	Appendix (referred to on p3)
Study selection	9	State the process for selecting studies (i.e., screening, eligibility, included in systematic review, and, if applicable, included in the meta-analysis).	3
Data collection process	10	Describe method of data extraction from reports (e.g., piloted forms, independently, in duplicate) and any processes for obtaining and confirming data from investigators.	3 (forms included in

			appendix)
Data items	11	List and define all variables for which data were sought (e.g., PICOS, funding sources) and any assumptions and simplifications made.	3
Risk of bias in individual studies	12	Describe methods used for assessing risk of bias of individual studies (including specification of whether this was done at the study or outcome level), and how this information is to be used in any data synthesis.	3
Summary measures	13	State the principal summary measures (e.g., risk ratio, difference in means).	3
Synthesis of results	14	Describe the methods of handling data and combining results of studies, if done, including measures of consistency (e.g., I^2) for each meta-analysis.	3,4

**Section/topic**	**#**	**Checklist item**	**Reported on page #**
Risk of bias across studies	15	Specify any assessment of risk of bias that may affect the cumulative evidence (e.g., publication bias, selective reporting within studies).	3
Additional analyses	16	Describe methods of additional analyses (e.g., sensitivity or subgroup analyses, meta-regression), if done, indicating which were pre-specified.	4
**RESULTS**			
Study selection	17	Give numbers of studies screened, assessed for eligibility, and included in the review, with reasons for exclusions at each stage, ideally with a flow diagram.	4, Figure 1, appendix
Study characteristics	18	For each study, present characteristics for which data were extracted (e.g., study size, PICOS, follow-up period) and provide the citations.	4, appendix
Risk of bias within studies	19	Present data on risk of bias of each study and, if available, any outcome level assessment (see item 12).	4, Figure 2
Results of individual studies	20	For all outcomes considered (benefits or harms), present, for each study: (a) simple summary data for each intervention group (b) effect estimates and confidence intervals, ideally with a forest plot.	4-8, Figures 3 and 4
Synthesis of results	21	Present results of each meta-analysis done, including confidence intervals and measures of consistency.	4-8, Figures 3 and 4
Risk of bias across studies	22	Present results of any assessment of risk of bias across studies (see Item 15).	Figure 2
Additional analysis	23	Give results of additional analyses, if done (e.g., sensitivity or subgroup analyses, meta-regression [see Item 16]).	4-8, appendix

DISCUSSION			
Summary of evidence	24	Summarize the main findings including the strength of evidence for each main outcome; consider their relevance to key groups (e.g., healthcare providers, users, and policy makers).	8,10,11
Limitations	25	Discuss limitations at study and outcome level (e.g., risk of bias), and at review-level (e.g., incomplete retrieval of identified research, reporting bias).	12
Conclusions	26	Provide a general interpretation of the results in the context of other evidence, and implications for future research.	10-12
**FUNDING**			
Funding	27	Describe sources of funding for the systematic review and other support (e.g., supply of data); role of funders for the systematic review.	1,4

From: Moher D, Liberati A, Tetzlaff J, Altman DG, The PRISMA Group (2009). Preferred Reporting Items for Systematic Reviews and Meta-Analyses: The PRISMA Statement. PLoS Med 6(7): e1000097. doi:10.1371/journal.pmed1000097

For more information, visit: **www.prisma-statement.org**.

Inclusion criteria

Table of outcomes included in the systematic review

Area of health	Outcomes	Outcome measures
Nutrition	Iron deficiency anaemia<br>Protein-energy malnutrition (stunting, wasting, underweight, overweight/obesity)	• Clinical outcomes: serum samples; anthropometric measurements
Mental health	Depression<br>Conduct disorder<br>Anxiety<br>Self-harm & suicide	• Clinical outcomes: clinical assessment<br>• Self-reported symptoms: validated mental health screening tools
Unintentional injuries	Road traffic incidents<br>Drowning	• Clinical outcomes: clinical assessment; records
Infectious disease	Intestinal infection<br>Lower respiratory tract infection<br>Encephalopathy<br>Malaria<br>Diarrhoea<br>HIV/AIDS<br>Meningitis<br>Neonatal sepsis	• Clinical outcomes: serum, sputum, faecal or urine tests; clinical assessment by a trained health professional<br>• Self-reported symptoms e.g. of diarrhoea
Key determinants of health	Substance use<br>Unprotected sex<br>Early pregnancy (<18 years)<br>Exposure to physical, emotional and sexual violence	• Self-reported symptoms: screening tools; reported substance use; exposure or perpetration of violence; pregnancy<br>• Clinical outcomes: clinical assessment; serum or urine test<br>• Intermediate behavioural outcomes: condom use

Full search strategy

Table of full search strategy

**Concept I: LMICs**	
1. Developing Countries.sh,kf	80835
2. (Africa or Asia or Caribbean or West Indies or South America or Latin America or Central America).hw,kf,ti,ab,cp.	228724
3. (Afghanistan or Albania or Algeria or Angola or Antigua or Barbuda or Argentina or Armenia or Armenian or Aruba or Azerbaijan or Bahrain or Bangladesh or Barbados or Benin or Byelarus or Byelorussian or Belarus or Belorussian or Belorussia or Belize or Bhutan or Bolivia or Bosnia or Herzegovina or Hercegovina or Botswana or Brasil or Brazil of Bulgaria or Burkina Faso or Burkina Fasso or Upper Volta or Burundi or Cambodia or Khmer Republic or Kampuchea or Cameroon or Cameroons or Cameron or Camerons or Cape Verde or Central African Republic or Chad or Chile or China or Colombia or Comoros or Comoro Islands or Comores or Mayotte or Congo or Zaire or Costa Rica or Cote d'Ivoire or Ivory Coast or Croatia or Cuba or Cyprus or Czechoslovakia or Czech Republic or Slovakia or Slovak Republic or Djibouti or French Somaliland or Dominica or Dominican Republic or East Timor or East Timur or Timor Leste or Ecuador or Egypt or United Arab Republic or El Salvador or Eritrea or Estonia or Ethiopia or Fiji or Gabon or Gabonese Republic or Gambia or Gaza or Georgia Republic or Georgian Republic or Ghana or Gold Coast or Greece or Grenada or Guatemala or Guinea or Guam or Guiana or Guyana or Haiti or Honduras or Hungary or India or Maldives or Indonesia or Iran or Iraq or Isle of Man or Jamaica or Jordan or Kazhakstan or Kazakh or Kenya or Kiribati or Korea or Kosovo or Kyrgystan or Kirghizia or Kyrgyz Republic or Kirghiz or Kirgizstan or Lao PDR or Laos or Latvia or Lebanon or Lesotho or Batusoland or Liberia or Libya or Lithuania or Macedonia or Madagascar or Malagasy Republic or Malaysia or Malaya or Malay or Sabah or Sarawak or Malawi or Nyasaland or Mali or Malta or Marshall Islands or Mauritania or Mauritius or Agalega Islands or Mexico or Micronesia or Middle East or Moldova or Moldovia or Moldovian or Mongolia or Montenegro or Morocco or Ifni or Mozambique or Muanmar or Myanma or Burma or Namibia or Nepal or Netherlands Antilles or New Caledonia or Nicaragua or Niger or Nigeria or Northern Mariana Islands or Oman or Muscat or Pakistan or Palau or Palestine of Panama or Paraguay or Peru or Philippines or Philipines or Phillipines or Phillippines or Poland or Portugal or Puerto Rico or Romania or Rumania or Roumania or Russia or Russian or Rwanda or Ruanda or Saint Kitts or St Kitts or Nevis or Saint Lucia or St Lucia or Saint Vincent or St Vincent or Grenadines or Samoa or Samoan Islands or Navigator Island or Navigator Islands or Sao Tome or Saudi Arabia or Senegal or Serbia or Montenegro or Seychelles or Sierra Leone or Slovenia or Sri Lanka or Ceylon or Solomon Islands or Somalia or South Africa or Sudan or Suriname or Surinam or Swaziland or Syria or Tajikistan or Tadzhikistan or Tadjikistan or Tadzhik or Tanzania or Thailand or Togo or Togolese Republic or Tonga or Trinidad or Tobago or Tunisia or Turkey or Turkmenistan or Turkmen or Uganda or Ukraine or Uruguay or USSR or Soviet Union or Union of Soviet Socialist Republics or Uzbekistan or Uzbek or Vanuatu or New Hebrides or Venezuela or Vietnam or Viet Nam or West Bank or Yemen or Yugoslavia or Zambia or Zimbabwe or Rhodesia).hw,kf,ti,ab,cp.	3142848
4. ((developing or less* developed or under developed or underdeveloped or middle income or low* income or underserved or under serve or deprived or poor*) adj (countr* or nation? or population? or world)).ti,ab.	77678
5. ((developing or less* developed or under developed or underdeveloped or middle income or low* income or underserved or under serve or deprived or poor*) adj (economy or economies)).ti,ab.	407
6. (low* adj (gdp or gnp or gross domestic or gross national)).ti,ab.	199
7. (low adj3 middle adj3 countr*).ti,ab.	8215
8. (lmic or lmics or third world or lami countr*).ti,ab.	4915
9. transitional countr*.ti,ab.	135
10. 1 or 2 or 3 or 4 or 5 or 6 or 7 or 8 or 9	3272683
**Concept II. Studies**	
11. exp Observational Study/	34454
12. exp Cohort Studies/	1669562
13. exp Case-Control Studies/	862245
14. exp Cross-Sectional Studies/	242969
15. exp "Review"/	2285378
16. (observational stud* or cohort stud* or case-control stud* or cross-sectional stud* or review*).mp. [mp=title, abstract, original title, name of substance word, subject heading word, keyword heading word, protocol supplementary concept word, rare disease supplementary concept word, unique identifier, synonyms]	3739790
17. 11 or 12 or 13 or 14 or 15 or 16	4980029
**Concept III: Child**	
18. exp Child/	1748124
19. exp Infant/	1052722
20. exp Infant, Newborn/	559976
21. exp Child, Preschool	838962
22. exp Adolescent/	1833132
23. (child* or infant or adolescen* or young adult* or teen* or young person* or juvenile or boy or girl or youth or pupil* or student* or newborn or baby).mp. [mp=title, abstract, original title, name of substance word, subject heading word, keyword heading word, protocol supplementary concept word, rare disease supplementary concept word, unique identifier, synonyms]	4243802
24. 18 or 19 or 20 or 21 or 22 or 23	4243802
**Concept IV: Left Behind**	
A) Parents	
25. exp Parents/	92582
26. exp Mothers/	35097
27. exp Fathers/	7588
28. exp Legal Guardians/	3355
29. exp Caregivers/	28538
30. (Parent* or mother or father or guardian* or caregiver*).mp. [mp=title, abstract, original title, name of substance word, subject heading word, keyword heading word, protocol supplementary concept word, rare disease supplementary concept word, unique identifier, synonyms]	569574
31. 25 or 26 or 27 or 28 or 29 or 30	589214

B) Migration	
32. exp “Emigration and Immigration”/	24380
33. exp Human Migration/	24964
34. exp “Transients and Migrants”/	9680
35. exp “Emigrants and Immigrants”/	8923
36. International labo?r migration.mp	78
37. Internal labo?r migration.mp	6
38. labo?r migrat*.mp. [mp= title, abstract, original title, name of substance word, subject heading word, keyword heading word, protocol supplementary concept word, rare disease supplementary concept word, unique identifier, synonyms]	885
39. labo?r migrant*.mp. [mp= title, abstract, original title, name of substance word, subject heading word, keyword heading word, protocol supplementary concept word, rare disease supplementary concept word, unique identifier, synonyms]	
40. international migrat*.mp. [mp= title, abstract, original title, name of substance word, subject heading word, keyword heading word, protocol supplementary concept word, rare disease supplementary concept word, unique identifier, synonyms]	156
41. migrant* worker*.mp. [mp= title, abstract, original title, name of substance word, subject heading word, keyword heading word, protocol supplementary concept word, rare disease supplementary concept word, unique identifier, synonyms]	3260
42. (migrat* or migrant* or emigrat* or immigrat* or transient*).mp. [mp= title, abstract, original title, name of substance word, subject heading word, keyword heading word, protocol supplementary concept word, rare disease supplementary concept word, unique identifier, synonyms]	1310
43. 32 or 33 or 34 or 35 or 36 or 37 or 38 or 39 or 40 or 41 or 42	606149
44. 31 and 43	12735
**Combining parents and migration**	
45. parent* migrat*.mp. 38. [mp= title, abstract, original title, name of substance word, subject heading word, keyword heading word, protocol supplementary concept word, rare disease supplementary concept word, unique identifier, synonyms]	78
46. 36 or 37 or 38 or 39 or 40 or 41 or 44 or 45	17402
**Key terms around transnational families / left behind families**	
47. labo?r sending countr*.mp. [mp= title, abstract, original title, name of substance word, subject heading word, keyword heading word, protocol supplementary concept word, rare disease supplementary concept word, unique identifier, synonyms]	9
48. transnational families.mp. [mp= title, abstract, original title, name of substance word, subject heading word, keyword heading word, protocol supplementary concept word, rare disease supplementary concept word, unique identifier, synonyms]	23
49. left behind families.mp. [mp= title, abstract, original title, name of substance word, subject heading word, keyword heading word, protocol supplementary concept word, rare disease supplementary concept word, unique identifier, synonyms]	4
50. remittance*.mp.	534
51. 47 or 48 or 49 or 50	555
**Key terms for left behind children**	
52. Left behind.mp.	1825
53. Left behind children.mp.	101
54. ((Left behind or unaccompanied or abandon* or left or leave or away or absen* or separat*) adj3 (child* or infant or adolescen* or young adult or youth or girl or boy or juvenile or teen* or newborn or baby)).mp. [mp= title, abstract, original title, name of substance word, subject heading word, keyword heading word, protocol supplementary concept word, rare disease supplementary concept word, unique identifier, synonyms]	10472
55. 52 or 53 or 54	12056
**Combining elements of concept IV**	
56. 46 or 51 or 55	29540
**Combining all concepts**	
57. 10 and 24 and 56	4650
58. 17 and 57	1379

16	12 or 13 or 14 or 15	4091793	Advanced	Display Results	More	
17	11 and 16	14403	Advanced	Display Results	More	
18	((parent* or mother or father) adj3 (absen* or abandon* or migrant* or migrat* or emigrat* or immigrat*)).mp. [mp=title, abstract, original title, name of substance word, subject heading word, keyword heading word, protocol supplementary concept word, rare disease supplementary concept word, unique identifier, synonyms]	1940	Advanced	Display Results	More	
19	exp Human Migration/	24890	Advanced	Display Results	More	
20	exp "Emigration and Immigration"/	24324	Advanced	Display Results	More	
21	exp "Transients and Migrants"/	9643	Advanced	Display Results	More	
22	19 or 20 or 21	31294	Advanced	Display Results	More	
23	18 or 22	33067	Advanced	Display Results	More	
24	10 and 17 and 23	233	Advanced	Display Results	More	

Data extraction including risk of bias

Template data extraction sheet for inclusion criteria

Item number	Inclusion criteria
	Study ID
	Reviewer
1	STUDY DESIGN: Is the study a case-control, cross-sectional or cohort study?
	Please provide details:
2	SETTING: Is the study set in a low- or middle-income country?
	Please provide details:
3	PARTICIPANTS: Are the study participants children aged 0-19 years?
	Please provide details:
4	PARTICIPANTS: Are the study participants children left behind by parents who have migrated?
	Please provide details:
5	COMPARATOR: Is there a control group of children whose parents are not migrants?
	Please provide details:
6	OUTCOMES: Does the study assess any of the following outcomes: nutrition, mental health, unintentional injuries, infectious disease, key determinants of health?
	Please provide details:
7	OVERALL: Does the study meet all inclusion criteria?
	Any further comments on inclusion and exclusion criteria:

Template data extraction sheet for general study information

Item number	General information
	Study ID
	Reviewer:
	A. Study data
1	Study title:
2	Purpose of the study:
3	Authors' conclusions:
4	Full reference:
5	Date of extraction:

6	Publication type:
7	Corresponding author:
8	Correspondence address (email):
	B. Conflicts of Interest
1	Copy of the funding, COI/acknowledgment section:
2	Funding source:
3	Are there any potential COI from funding?
4	Are there any other potential COI?

Template data extraction sheet for study design and setting

Item number	Study design and setting
	Study ID
	Reviewer:
	A. Study Design
1	What was the study design?
2	Did the study use primary or secondary data?
3	If the study used secondary data, please provide the name of the dataset
4	When was the study carried out (dates and duration)?
	B. Setting
5	In which country was the study based?
6	To which income group does this country belong?
7	In which city/town/area was the study based?
8	Any other relevant information about the setting (e.g. rural, school-based)?

Template data extraction sheet for participant information

Item number	Participants
	Study ID
	Reviewer:
A. General Information	
1	General description (or definition) of the left behind children
2	General description (or definition) of the control group(s)
3	What ages were the participants?
4	What gender were the participants?
B. Recruitment	
4	List of all inclusion and exclusion criteria for study participation
5	How were potential participants approached and invited to participate?
6	Comments on the representativeness of the sample:

C. Parents	
7	Drivers of parental migration, if known
8	Duration and other characteristics of parental absence, if known
9	Did the mother, father, or both parents migrate?
10	Destination town/country of parents' migration, if known
11	Primary caregivers in the absence of parents, if known
D. Other	
12	Any other relevant data regarding participant characteristics

Template data extraction sheet for general outcomes

Item number	Outcomes
	Study ID
	Reviewer:
A. Overview	
1	Primary study outcomes (as defined by authors)
2	Secondary study outcomes (as defined by authors)
3	Were there any outcomes related to NUTRITION?
4	Were there any outcomes related to MENTAL HEALTH?
5	Were there any outcomes related to UNINTENTIONAL INJURIES?
6	Were there any outcomes related to INFECTIOUS DISEASE?
7	Were there any outcomes related to KEY DETERMINANTS OF HEALTH?
B. Results	
8	Was a power calculation done?
9	Number of children eligible or invited to take part
10	<u>Total</u> number of children who participated
11	Number of <u>left-behind children</u> who participated
12	Number of <u>control</u> children who participated
13	Response rate
14	Were any attempts made to impute missing data?

Template data extraction sheet for nutritional outcomes

Item number	Outcomes: Nutrition
	Study ID
	Reviewer:
A. NUTRITION: OUTCOME 1	
1	Outcome 1:
2	Authors' definition of outcome 1
3	What was the mode of outcome assessment

4	By whom was the outcome assessed (if applicable)?
5	Which tool was used for the outcome assessment (if applicable)?
6	Has the assessment tool or measurement method been validated in this population or setting?
7	Description of the statistical method applied:
8	Were any subgroup analyses done?
9	Is any information on moderators or mediators discussed in the study?
10	Description of any moderators or mediators, if applicable:
11	Narrative summary of results for this outcome:
12	Was there a significant difference between left behind children and controls?
13	Mean (or other outcome measure) for left behind children:
14	Mean (or other outcome measure) for control group:
15	Effect estimate
B. NUTRITION: OUTCOME 2	
1	Outcome 2
2	Authors' definition of outcome 2
3	What was the mode of outcome assessment
4	By whom was the outcome assessed (if applicable)?
5	Which tool was used for the outcome assessment (if applicable)?
6	Has the assessment tool or measurement method been validated in this population or setting?
7	Description of the statistical method applied:
8	Were any subgroup analyses done?
9	Is any information on moderators or mediators discussed in the study?
10	Description of any moderators or mediators, if applicable:
11	Narrative summary of results for this outcome:
12	Was there a significant difference between left behind children and controls?
13	Mean (or other outcome measure) for left behind children:
14	Mean (or other outcome measure) for control group:
15	Effect estimate
B. NUTRITION: OUTCOME 3	
1	Outcome 3
2	Authors' definition of outcome 3
3	What was the mode of outcome assessment
4	By whom was the outcome assessed (if applicable)?
5	Which tool was used for the outcome assessment (if applicable)?
6	Has the assessment tool or measurement method been validated in this population or setting?
7	Description of the statistical method applied:
8	Were any subgroup analyses done?
9	Is any information on moderators or mediators discussed in the study?
10	Description of any moderators or mediators, if applicable:

11	Narrative summary of results for this outcome:
12	Was there a significant difference between left behind children and controls?
13	Mean (or other outcome measure) for left behind children:
14	Mean (or other outcome measure) for control group:
15	Effect estimate
B. NUTRITION: OUTCOME 3	
1	Outcome 3
2	Authors' definition of outcome 3
3	What was the mode of outcome assessment
4	By whom was the outcome assessed (if applicable)?
5	Which tool was used for the outcome assessment (if applicable)?
6	Has the assessment tool or measurement method been validated in this population or setting?
7	Description of the statistical method applied:
8	Were any subgroup analyses done?
9	Is any information on moderators or mediators discussed in the study?
10	Description of any moderators or mediators, if applicable:
11	Narrative summary of results for this outcome:
12	Was there a significant difference between left behind children and controls?
13	Mean (or other outcome measure) for left behind children:
14	Mean (or other outcome measure) for control group:
15	Effect estimate

Template data extraction sheet for mental health outcomes

Item number	Outcomes: Mental Health
	Study ID
	Reviewer:
A. MENTAL HEALTH: OUTCOME 1	
1	Outcome 1:
2	Authors' definition of outcome 1
3	What was the mode of outcome assessment
4	By whom was the outcome assessed (if applicable)?
5	Which tool was used for the outcome assessment (if applicable)?
6	Has the assessment tool or measurement method been validated in this population or setting?
7	Description of the statistical method applied:
8	Were any subgroup analyses done?
9	Is any information on moderators or mediators discussed in the study?
10	Description of any moderators or mediators, if applicable:
11	Narrative summary of results for this outcome:

12	Was there a significant difference between left behind children and controls?
13	Mean (or other outcome measure) for <u>left behind children</u>:
14	Mean (or other outcome measure) for <u>control</u> group:
15	Effect estimate
B. MENTAL HEALTH: OUTCOME 2	
1	Outcome 2:
2	Authors' definition of outcome 2
3	What was the mode of outcome assessment
4	By whom was the outcome assessed (if applicable)?
5	Which tool was used for the outcome assessment (if applicable)?
6	Has the assessment tool or measurement method been validated in this population or setting?
7	Description of the statistical method applied:
8	Were any subgroup analyses done?
9	Is any information on moderators or mediators discussed in the study?
10	Description of any moderators or mediators, if applicable:
11	Narrative summary of results for this outcome:
12	Was there a significant difference between left behind children and controls?
13	Mean (or other outcome measure) for <u>left behind children</u>:
14	Mean (or other outcome measure) for <u>control</u> group:
15	Effect estimate

Template data extraction sheet for injuries

Item number	Outcomes: Unintentional Injuries
	Study ID
	Reviewer:
A. UNINTENTIONAL INJURIES: OUTCOME 1	
1	Outcome 1:
2	Authors' definition of outcome 1
3	What was the mode of outcome assessment
4	By whom was the outcome assessed?
5	Was a standardised tool or definition used for the outcome assessment (if applicable)?
6	Description of the statistical method applied:
7	Were any subgroup analyses done?
8	Is any information on moderators or mediators discussed in the study?
9	Description of any moderators or mediators, if applicable:
10	Narrative summary of results for this outcome:
11	Was there a significant difference between left behind children and controls?
12	Mean (or other outcome measure) for <u>left behind children</u>:

13	Mean (or other outcome measure) for control group:
14	Effect estimate
B. UNINTENTIONAL INJURIES: OUTCOME 2	
1	Outcome 2:
2	Authors' definition of outcome 2
3	What was the mode of outcome assessment
4	By whom was the outcome assessed?
5	Was a standardised tool or definition used for the outcome assessment (if applicable)?
6	Description of the statistical method applied:
7	Were any subgroup analyses done?
8	Is any information on moderators or mediators discussed in the study?
9	Description of any moderators or mediators, if applicable:
10	Narrative summary of results for this outcome:
11	Was there a significant difference between left behind children and controls?
12	Mean (or other outcome measure) for left behind children:
13	Mean (or other outcome measure) for control group:
14	Effect estimate

Template data extraction sheet for infectious disease

Item number	Outcomes: Infectious Disease
	Study ID
	Reviewer:
A. INFECTIOUS DISEASES: OUTCOME 1	
1	Outcome 1:
2	Authors' definition of outcome 1:
3	What was the mode of outcome assessment?
4	By whom was the outcome assessed?
5	Description of the statistical method applied:
6	Were any subgroup analyses done?
7	Is any information on moderators or mediators discussed in the study?
8	Description of any moderators or mediators, if applicable:
9	Narrative summary of results for this outcome:
10	Was there a significant difference between left behind children and controls?
11	Mean (or other outcome measure) for left behind children:
12	Mean (or other outcome measure) for control group:
13	Effect estimate
B. INFECTIOUS DISEASES: OUTCOME 2	
1	Outcome 2:

2	Authors' definition of outcome 2:
3	What was the mode of outcome assessment?
4	By whom was the outcome assessed?
5	Description of the statistical method applied:
6	Were any subgroup analyses done?
7	Is any information on moderators or mediators discussed in the study?
8	Description of any moderators or mediators, if applicable:
9	Narrative summary of results for this outcome:
10	Was there a significant difference between left behind children and controls?
11	Mean (or other outcome measure) for left behind children:
12	Mean (or other outcome measure) for control group:
13	Effect estimate

Template data extraction sheet for key determinants of health

Item number	Outcomes: Key Determinants of Health
	Study ID
	Reviewer:
A. KEY DETERMINANTS OF HEALTH: OUTCOME 1	
1	Outcome 1:
2	Authors' definition of outcome 1:
3	What was the mode of outcome assessment?
4	By whom was the outcome assessed?
5	Which tool was used for the outcome assessment (if applicable)?
6	Has the assessment tool or measurement method been validated? (Please provide details, if applicable)
7	Description of the statistical method applied:
8	Were any subgroup analyses done?
9	Is any information on moderators or mediators discussed in the study?
10	Description of any moderators or mediators, if applicable:
11	Narrative summary of results for this outcome:
12	Was there a significant difference between left behind children and controls?
13	Mean (or other outcome measure) for left behind children:
14	Mean (or other outcome measure) for control group:
15	Effect estimate
B. KEY DETERMINANTS OF HEALTH: OUTCOME 2	
1	Outcome 2:
2	Authors' definition of outcome 2:
3	What was the mode of outcome assessment?
4	By whom was the outcome assessed?

5	Which tool was used for the outcome assessment (if applicable)?
6	Has the assessment tool or measurement method been validated? (Please provide details, if applicable)
7	Description of the statistical method applied:
8	Were any subgroup analyses done?
9	Is any information on moderators or mediators discussed in the study?
10	Description of any moderators or mediators, if applicable:
11	Narrative summary of results for this outcome:
12	Was there a significant difference between left behind children and controls?
13	Mean (or other outcome measure) for left behind children:
14	Mean (or other outcome measure) for control group:
15	Effect estimate

Template data extraction sheet for risk of bias

Item number	Risk of Bias Assessment
	Study ID
	Reviewer:
	A. POPULATION
1	Were left behind children well defined?
	Please provide reasons for your judgement:
2	Were children in the control group well described/defined?
	Please provide reasons for your judgement:
	B. SELECTION
3	Was selection bias minimised?
	Please provide reasons for your judgement:
	C. OUTCOME
4	Were the outcome measures and procedures reliable?
	Please provide reasons for your judgement:
5	Were all outcome measures reported?
	Please provide reasons for your judgement:
	D. ANALYSIS
6	Was the study sufficiently powered to detect a difference, if one exists?
	Please provide reasons for your judgement:
7	Were the statistical analyses clear and presented with a measure of precision?
	Please provide reasons for your judgement:
8	Was incomplete outcome data adequately addressed?
	Please provide reasons for your judgement:
9	Were potential confounders adequately addressed?
	Please provide reasons for your judgement:

Excluded studies

Exclusion codes:

1. <50% of the participants were aged 0-19 years or the mean or median age is above 19
2. Study conducted in a HIC
3. Study is not a cohort, case-control, or cross-sectional
4. Study does not include left behind children*
5. Study does not compare left behind children' with children whose parents are not migrants
6. Not an outcome of interest
7. Reviews
8. Cannot locate full text
9. Conference proceedings
10. Protocol for a study not meeting the inclusion criteria
11. Ongoing studies
12. Other – please specify

Tables of included studies

Table of study characteristics: mental health outcomes

**Study** *Country*	**Design**	**Definition of LBC and type of migration**	**Outcome**	**Instrument (cut-off)**	**No. of LBC**	**No. of controls**	**Effect estimate (95% CI)**
Adhikari 2014† *Thailand* [21]	CS	1 or both parents and international labour migrants; min. absence 6m<br><br>3-5y and 9-11y<br>Gender NR	Conduct disorder	SDQ (≥19)	519	511	Proportion<br>LBC: 18.1% vs. controls: 13.1%; not statistically significant
Aguilera-Guzman 2004 *Mexico* [22]	CS	Father is international labour migrant; duration NR<br><br>11-14y; mean (sd) 11.5 (0.72)<br>51% male	Depression	CES-D (mean)	109	210	Mean (sd)<br>LBC: 19.8 (7.6) vs. controls 19.5 (7.6); not statistically significant
Asis 2013† *Philippines* [23]	CS	1 or both parents are international labour migrants; min. absence 1y<br><br>10-12y; mean (sd) 10.7 (1.15)<br>46.0% male	Anxiety	SAS (mean)	Father absent: 628<br>Mother absent: 347<br>Both absent: 150	318	Anxiety: mean (sd)<br>Father absent: 4.84 (2.42)<br>Mother absent: 4.97 (2.63)<br>Both absent: 4.75 (2.41)<br>Controls 5.18 (2.30)<br>No significance testing reported
Battistella 1998† *Philippines* [24]	CS	1 or both parents are international labour migrants; mean absence 4.9y<br><br>9-15y; mean 11.3y<br>45.8% male	Anxiety	SASC (mean)	Father absent: 206<br>Mother absent: 186<br>Both absent: 116	201	Mean<br>Both parents absent: 8.90<br>Father migrated: 9.13<br>Mother migrated: 8.98<br>Controls: 9.30<br>No significance testing reported

**Study** *Country*	**Design**	**Definition of LBC and type of migration**	**Outcome**	**Instrument (cut-off)**	**No. of LBC**	**No. of controls**	**Effect estimate (95% CI)**
Bi 2015† *China* [25]	CS	1 or both parents are internal labour migrants; mean (sd) absence 7.0 (3.8) yrs<br><br>Mean (sd) 13.67y (0.83)<br>56.9% male	Depression	CES-D for Children (mean)	93	42	<u>Mean (sd)</u><br>LBC: 15.26 (9.47) vs. controls: 13.61 (6.84); p=0.346
Chen 2016† *China* [26]	CS	1 or both parents are internal labour migrants; duration NR<br><br>Mean (sd) 13y (1.71). Students between grades 4 and 9<br>52.0% male	Depression	CES-D for Children (≥15)	793	245	<u>OR (95% CI):</u><br>1.51 (1.07–2.11); adjusted for demographic factors
Cheng 2008† *China* [27]	CS	1 or both parents are internal labour migrants; min. absence 6m<br><br>Ages NR; middle and high school students<br>Gender distribution NR	Depression<br><br>Anxiety	SCL-90 (≥3)<br><br>SCL-90	2323	1032	<u>Depression:</u><br>LBC: 7.4% vs. controls 4.4%; χ^2 9.45<br><br><u>Anxiety:</u><br>LBC 12.5% vs. controls: 5.7%; χ^2 32.56<br><br>LBC significantly more likely to have depression and anxiety than controls
Deng 2014† *China* [28]	CS	1 or both parents are internal labour migrants; duration NR<br><br>Under 14y<br>50.2% male	Suicidal ideation	Self-Rating Idea of Suicide Scale (≥12)	2065	1680	<u>Proportion:</u><br>LBC: 12.9% vs. controls: 9.4%; χ^2 11.15

Study<br>*Country*	Design	Definition of LBC and type of migration	Outcome	Instrument (cut-off)	No. of LBC	No. of controls	Effect estimate (95% CI)
Fan 2010† *China* [29]	CS	Both parents are internal labour migrants; min. absence 12m<br><br>0-7y<br>51.9% male	Conduct disorder	SDQ<br>(mean)	629	645	Mean (sd)<br>LBC: 2.0 (2.4) vs. controls: 1.6 (2.0)
Feng 2014† *China* [30]	CS	1 or both parents are internal labour migrants; min. absence 6m<br><br>Mean age (sd) 9.3y (1.2); range 6-15y<br>55.3% male	Anxiety	MHT (Chinese)	264	166	Proportion:<br>LBC: 32.95% vs. controls: 10.24%; χ^2 28.67
Gao 2008† *China* [31]	CS	1 or both parents are internal labour migrants; min. absence 6m<br><br>Age distribution NR<br>Gender distribution NR	Depression<br>Anxiety	SCL-90 (mean)	756	623	Depression: mean<br>1 parent absent: 0.38<br>2 parents absent: 0.41<br>Controls: 0.34; F=3.234<br><br>Anxiety: mean<br>1 parent absent: 0.33<br>2 parents absent: 0.34<br>Controls: 0.26; F=3.952<br><br>No significance testing reported
Gao 2010† *China* [13]	CS	1 or both parents are internal labour migrants; min. absence 6m<br><br>10-18y; mean 14.2y (sd 1.4), median 14.0y	Suicidal ideation	Adapted YRBS and GSHS	541	2445	Suicidal ideation<br>uOR males: 2.1 (0.84-5.26), p=0.09<br>aOR males: 2.32 (1.33-4.04), p<0.01<br>Females not compared

Study *Country*	Design	Definition of LBC and type of migration	Outcome	Instrument (cut-off)	No. of LBC	No. of controls	Effect estimate (95% CI)
		51.4 % male					<u>Suicide planning</u><br>Non-significant [effect estimate NR]<br><br><u>Suicide attempt</u><br>uOR males: 1.37 (0.97-1.94), p<0.01<br>aOR males: 0.50 (0.08-3.38), p=0.48<br>Females not compared
Ge 2011† *China* [32]	CS	Both parents are rural-to-urban labour migrants; duration NR<br><br>Age NR. Year 3, 5 and 8 students<br>Gender distribution NR	Anxiety	Self-Rating Anxiety Scale (mean)	112	105	P<u>roportion:</u><br>LBC: 42.5% vs. controls: 38.9%; t=3.478<br>No significance testing reported
Graham 2011† *Indonesia, Philippines, Thailand, Vietnam* [33]	CS	1 or both parents are international labour migrants; duration NR<br><br>Two age groups 3-5y and 9-11y of approximately equal size<br><br>Approximately equal males and females (numbers not specified)	Conduct disorder	SDQ (>3 on conduct disorder subscale)	<u>Total: 1916</u><br>Indonesia: 465<br>Philippines: 467<br>Thailand: 483<br>Vietnam: 501	<u>Total: 1960</u><br>Indonesia: 496<br>Philippines: 491<br>Thailand: 512<br>Vietnam: 461	<u>OR (95% CI) [adjusted for age & sex]:</u><br>Indonesia 0.97 (0.70–1.36)<br>Philippines 0.69 (0.51–0.93)<br>Thailand 1.41 (1.05–1.89)<br>Vietnam 0.68 (0.43–1.06)
Guo 2012† *China* [34]	CS	1 or both parents are internal labour migrants; min. duration 6m<br><br>8-17y; mean (sd) 12.56 (1.83)<br>48.0% male	Depression	CDI-SF (≥7)	1143	1287	<u>Proportions:</u><br>LBC 14.3%<br>Controls 10.9%<br><br>The prevalence of depression

Study *Country*	Design	Definition of LBC and type of migration	Outcome	Instrument (cut-off)	No. of LBC	No. of controls	Effect estimate (95% CI)
							was higher among LBC. No significance testing reported
Guo 2015† *China* [35]	CS	1 or both parents are internal labour migrants; min. duration 6m<br><br>8-17y; mean (sd) 12.64y (1.91)<br>51.5% male	Depression	CDI-SF<br>(≥7 and mean)	Total: 1247<br>1 parent absent: 824<br>2 parents absent: 423	1944	Proportion:<br>1 parent absent: 13.1%<br>2 parents absent: 16.1%<br>Controls: 10.5%<br><br>Mean (sd)<br>1 parent absent: 3.41 (2.80)<br>2 parents absent: 3.71 (3.05)<br>Controls: 3.12 (2.90)<br><br>Children left by both parents had higher depression scores than controls. [Unclear difference between 1-parent absent vs. controls]. No significance testing reported.
He 2012† *China* [36]	CS	1 or both parents are internal labour migrants; min. duration 6m<br><br>9-14y, mean (sd) 11.08y (SD = 1.09)<br>Gender distribution NR	Depression	CDI<br>(mean)	590	285	Mean (sd):<br>LBC: 11.44 (7.21) vs. controls: 8.02 (5.83)<br><br>OR (95% CI):<br>Father migrant: 3.42 (1.86-6.28)<br>Mother migrant: 2.62 (1.10-6.22)<br>Both parents migrants: 2.73 (1.77-4.20)

**Study** *Country*	**Design**	**Definition of LBC and type of migration**	**Outcome**	**Instrument (cut-off)**	**No. of LBC**	**No. of controls**	**Effect estimate (95% CI)**
Hou 2014 China [37]	CS	1 or both parents are labour migrants; duration NR<br><br>7.9-18.5y; mean (sd) 12.7 (1.9)<br>51.0% male	Depression	CDI	1457	2355	OLS showed that LBC (both 1-parent and both-parents absent) were more depressed than controls, but the difference became non-significant after propensity score matching.
Hu 2014† *China* [38]	CS	1 or both parents are labour migrants; min. duration 6m<br><br>5-17; mean (sd) 10.95 (2.66)<br>50.79% male	Conduct disorder	SDQ (mean)	694	1459	Mean (sd):<br>LBC: 1.99 (1.64) vs. controls: 1.76 (1.64). Not significant after controlling for confounding variables
Jiang 2013† *China* [39]	CS	1 or both parents are labour migrants; duration NR<br><br>13-15y; mean (sd) 13.93 (0.78)<br>53.2% male	Depression<br><br>Conduct disorder	SDQ (>7)	963	1520	Depression: proportion<br>LBC: 6.1% vs. controls: 6.0%<br><br>Conduct disorder: proportion<br>LBC: 7.3% vs. controls: 7.2%<br><br>No significant difference in prevalence of depression or conduct disorders between LBC and controls
Jin & Wu 2009 *China* [40]	CS	1 or both parents are labour migrants; duration NR<br><br>8-16y; mean (sd) 12.26 (1.40)<br>56.3% male	Anxiety	MHT(Chinese) (mean)	748	531	Mean<br>LBC: 39.99 vs. controls: 38.87<br>No significant difference

**Study** *Country*	**Design**	**Definition of LBC and type of migration**	**Outcome**	**Instrument (cut-off)**	**No. of LBC**	**No. of controls**	**Effect estimate (95% CI)**
Jones 2004 *Trinidad and Tobago* [41]	CS	1 or both parents are international labour migrants; duration 2–15y<br><br>13-16y<br>53.0% male	Depression	CDI<br>(T-score 60)	74	72	LBC scored significantly higher on CDI and a greater number demonstrated symptoms suggestive of emotional or behavioural problems
Lan 2009[†] *China* [42]	CS	1 or both parents are internal labour migrants; min. duration 6m<br><br>7-16y<br>Gender distribution NR	Depression<br><br>Conduct disorder	CES-D<br><br>Rutter Children Behaviour Questionnaire	270	609	Depression: mean:<br>LBC: 18.24 vs. controls 15.85; t=3.95<br>Depression: proportion:<br>LBC 40.4% vs. controls 27.8%; χ^2 13.78<br><br>LBC had significantly higher depression than non-LBC<br><br>Conduct disorder: mean:<br>LBC: 5.06 vs. controls 5.80; t=1.85<br>Conduct disorder: proportion:<br>LBC 15.6% vs. controls 22.7%; χ^2 5.8<br><br>Control group had significantly higher prevalence of conduct disorder. No significant difference in conduct scores.

**Study** *Country*	**Design**	**Definition of LBC and type of migration**	**Outcome**	**Instrument (cut-off)**	**No. of LBC**	**No. of controls**	**Effect estimate (95% CI)**
Li 2008† *China* [43]	CS	1 or both parents are internal labour migrants; min. duration 6m<br><br>12-16y<br>53.0% male	Conduct disorder	Child Behaviour Checklist (NR)	3589	2384	Proportion:<br>LBC: 41.3% vs. controls: 36.6%; χ^2 13.19 LBC significantly more likely to experience conduct disorder compared with controls.
Li 2009† *China* [44]	CS	1 or both parents are internal labour migrants; duration NR<br><br>14-16y<br>13.1% male	Suicidal thoughts, planning, behaviour<br><br>Depression<br><br>Anxiety	Self-rating Depression Scale (>41)<br><br>Self-rating Anxiety Scale (>40)	334	506	Suicidal ideation<br>Both parents absent: 27.4%<br>Father absent: 63.8%<br>Mother absent: 64.1%<br>Controls: 29.8% (p<0.001)<br><br>Suicide planned<br>Both parents absent: 13.0%<br>Father absent: 10.3%<br>Mother absent: 15.4%<br>Controls: 12.8%<br><br>Suicide behaviour<br>Both parents absent: 3.4%<br>Father absent: 4.7%<br>Mother absent: 2.6%<br>Controls: 7.9%<br><br>Depression:<br>LBC: 34.2% vs. controls: 32.3%; NS<br><br>Anxiety:<br>LBC: 12.3% vs. controls:

**Study** ***Country***	**Design**	**Definition of LBC and type of migration**	**Outcome**	**Instrument (cut-off)**	**No. of LBC**	**No. of controls**	**Effect estimate (95% CI)**
							13.6%; NS
Liao 2013† *China* [45]	CS	1 or both parents are internal labour migrants; min. duration 6m<br><br>2-16y<br>47.7% male	Conduct disorder	Child Behavior Checklist	Total: 2055<br>1 parent away: 966<br>2 parents away: 1059	1050	LBC were more likely to have behavioral problems than non-LBC among the 2-3 year age group. Left-behind boys were more likely to have behavioral problems than non-left-behind boys. No significant differences in the older age groups.
Ling 2015† *China* [46]	CS	1 or both parents are internal labour migrants; min. duration 6m<br><br>Mean (sd) age 11.88y (1.67)<br>54.9% male	Conduct disorder<br><br>Depression	YSR (mean)<br><br>DSRSC (mean)	268	228	Mean (SD):<br>Conduct disorder: LBC: 56.81 (22.94) vs. controls: 47.89 (20.44)<br>Depression: LBC: 12.51 (3.97) vs. controls: 11.78 (3.78)<br><br>LBC had significantly higher depression symptoms
Liu & Yang 2012† *China* [47]	CS	1 or both parents are internal labour migrants<br><br>12-16y; mean (sd) 14.02y (1.46)<br>52.7% male	Conduct disorder	Emotional Problem Behavior Assessment Questionnaire (Chinese)	205	235	Mean:<br>LBC: 42.95<br>Controls: 27.86<br>No significance testing reported

Study *Country*	Design	Definition of LBC and type of migration	Outcome	Instrument (cut-off)	No. of LBC	No. of controls	Effect estimate (95% CI)
Pottinger 2005† *Jamaica* [48]	CC	1 or both parents are international migrants; min. duration 6m<br><br>9-10y<br>52.0% male	Suicidal ideation	12-item list of depressive symptoms	27	27	Mean scores (sd):<br>Boys: LBC 2 +/-15 vs. controls: 1 +/- 7<br>Girls: LBC: 2 +/- 15; Controls: 2 +/- 15<br>ANOVA revealed no statistically significant differences between cases and controls, but parental migration was associated with suicidal thoughts (r=41, p<0.05)
Qiao 2008† *China* [49]	CS	1 or both parents are internal labour migrants; parents came home no more than once every 6m<br><br>Age NR (years 3-5 primary school students)<br>53.0% male	Anxiety	MHT (Chinese)	564	275	Proportion:<br>LBC 19.3%<br>Controls: 5.8%<br>χ^2 26.6
Qu 2015† *China* [50]	CS	1 or both parents are internal labour migrants; min. duration 6m<br><br>6-16y<br>50.42% male	Anxiety<br><br>Conduct disorder	MINI-KID	7331	12380	Anxiety: LBC: 4.5% (4.0–5.0) *vs.* controls 2.1% (1.8–2.3): OR 2.22 (1.9–2.6)<br><br>Conduct disorder: LBC: 15.3% (14.5–16.1) vs. controls: 4.3% (4.0–4.5); OR 3.99 (3.6-4.5)
Ren 2016 *China* [51]	CS	1 or both parents are internal labour migrants; duration NR<br><br>10-15y	Depression	CES-D (six items)<br>(mean)	725	2629	Means:<br>1 parent LBC: 0.13; 2 parents LBC: 0.15<br>Controls (rural intact family):

Study *Country*	Design	Definition of LBC and type of migration	Outcome	Instrument (cut-off)	No. of LBC	No. of controls	Effect estimate (95% CI)
		Gender distribution NR					0.11<br>No significance testing reported
Shen 2015[†]<br>*China* [52]	CS	1 or both parents are internal labour migrants; min. duration 6m<br><br>10-18y; mean 14.22y (sd 1.71y)<br>45.0% male	Depression<br><br>Anxiety	CDI<br>(>20)<br><br>SCARED (>25)	1397	886	Depression:<br>1 parent absent: 11.7%<br>Both parents absent: 14.2%<br>Controls: 12.6%<br><br>Anxiety:<br>1 parent absent: 22.0%<br>Both parents absent: 22.2%<br>Controls: 25.1%<br><br>Parental migration was not significantly associated with depression and anxiety
Shi 2016a[†]<br>*China* [53]	CS	Both parents are internal labour migrants; 50% migrated for 1 yr; 21% for 1–3 yrs; remainder for >3 yrs<br><br>16-21y; mean 18.27y (sd 0.79)<br>61.2% male	Depression<br><br>Anxiety	SCL-90 (mean)	1063	1905	Mean scores (sd):<br>Depression:<br>LBC: 1.56 (0.56); Controls: 1.48 (0.51); p=0.000<br><br>Anxiety:<br>LBC: 1.54 (0.52); Control: 1.47 (0.47) ; p=0.000<br><br>Significantly increased risk of depression and anxiety among LBC

**Study** ***Country***	**Design**	**Definition of LBC and type of migration**	**Outcome**	**Instrument (cut-off)**	**No. of LBC**	**No. of controls**	**Effect estimate (95% CI)**
Shi 2016b *China* [54]	CS	Both parents are internal labour migrants; min. duration 8m<br><br>Grade 4 and 5 children; ages NR<br>Gender distribution NR	Anxiety	SASC (mean)	1130	6251	Mean scores of LBC increased by 0.32 compared with control children (p=0.01)
Sun 2015 China [55]	CS	1 or both parents are internal labour migrants; 39.3% migrated >5 years; 36.5% migrated 1–5 years<br><br>15.03 +/- 1.93<br>45.2% male	Depression	CDI<br>no cut-off	1108	600	LBC with both parents who migrated had significantly higher levels of depression than controls (p=0.006).<br><br>LBC with one parent at home fared better than those with both parents absent (no quantitative results reported).
Tao 2016[†] *China* [56]	CS	1 or both parents are internal labour migrants; min. duration 6m<br><br>7-15y<br>51.2% female	Conduct disorder	Modified Chinese JEPQ-R	472	355	Extraversion mean:<br>LBC 38.46 (11.33) vs. controls 39.15 (12.09); non-significant<br><br>Neuroticism mean:<br>LBC: 48.31 (8.37) vs. controls: 42.79 (9.29); p<0.01<br><br>Psychoticism mean:<br>LBC: 46.71 (6.09) vs. controls: 45.12 (5.27); p<0.01<br><br>Lie:<br>LBC: 49.28 (8.77) vs. controls: 52.12 (6.36); p<0.01

**Study** *Country*	**Design**	**Definition of LBC and type of migration**	**Outcome**	**Instrument (cut-off)**	**No. of LBC**	**No. of controls**	**Effect estimate (95% CI)**
Tomsa 2015[†] *Romania* [57]	CS	1 or both parents are international labour migrants; duration NR<br><br>12-15y; LBC mean (sd) 13.5 (1.0); control mean (sd) 13.3 (1.0)<br>51.5% males	Anxiety<br><br>Depression	STAIC (mean)<br><br>SMFQ >8 (mean)	163	163	State anxiety mean (sd):<br>LBC: 29.73 (5.7) vs. controls: 28.32 (5.27); p=0.02<br><br>Trait anxiety mean (sd):<br>LBC: 34.5 (7.31) vs. controls: 32.68 (6.16); p=0.02<br><br>Depression mean (sd):<br>LBC: 8.76 (5.72)vs. controls: 7.24 (5.15); p=0.01
Vanore 2015[†] *Moldova* [58]	CS	1 or both parents are international labour migrats; min. duration 12m<br><br>4-17y; mean (sd) 10.8y (4.03)<br>49.9% male	Conduct disorder	SDQ (mean)	471	1508	*Males:* mother absent: 1.77 (1.62); father absent: 2.16 (1.19); both parents absent: 1.79 (1.52); controls: 1.55 (1.58)<br><br>*Females:* mother absent: 1.18 (1.77); father absent: 1.03 (1.19); both parents absent: 1.25 (1.41); controls: 1.20 (1.32)
Wan 2009[†] *China* [59]	CS	1 or both parents are internal labour migrants; min. duration 6m<br><br>7-17y; mean (sd) 12.51y (2.20)<br>58.4% male	Anxiety	SASC	762	272	Primary school:<br>Father absent 6.47; Mother absent 8.00; Both absent 6.98; Controls 6.67; F=3.8<br><br>Middle school:<br>Father absent 8.01; Mother

**Study** ***Country***	**Design**	**Definition of LBC and type of migration**	**Outcome**	**Instrument (cut-off)**	**No. of LBC**	**No. of controls**	**Effect estimate (95% CI)**
							absent 7.40; Both absent 8.12; Controls 7.39; F=2.6
Wang & Chen 2010† *China* [60]	CS	1 or both parents are internal labour migrants; duration NR<br><br>8-14y<br>49.7% male	Anxiety	SASC (>9.5)	365	255	Prevalence:<br>LBC: 24.4% vs. controls: 6.7%<br><br>Mean:<br>LBC: 7.03 vs. controls: 5.04; t=7.60
Wang & Chen 2011† *China* [61]	CS	1 or both parents are internal labour migrants; min. duration 6m<br><br>Age distribution NR<br>52.6% male	Depression	CDI (>19)	1694	1223	Prevalence<br>LBC: 12.1% vs. controls: 8.0%<br>OR (95% CI) 1.45 (1.11–1.90)
Wang & Deng 2010† *China* [62]	CS	1 or both parents are internal labour migrants; duration NR<br><br>6-16y<br>Gender distribution NR	Depression<br><br>Anxiety	SCL90 (mean)	1284	1264	Depression:<br>Both parents absent: 0.38; Mother absent: 0.36; Father absent: 0.34; Controls 0.32; F=1.009<br><br>Anxiety:<br>Both parents absent: 0.35; Mother absent: 0.30; Father absent: 0.29; Controls 0.25; F=5.322<br><br>Children left by both parents had higher depression and anxiety than controls. No

Study *Country*	Design	Definition of LBC and type of migration	Outcome	Instrument (cut-off)	No. of LBC	No. of controls	Effect estimate (95% CI)
							interpretation of children left by 1 parent vs. controls. No significance testing reported
Wang & Zhu 2012† *China* [63]	CS	1 or both parents are internal labour migrants; min. duration 6m<br><br>10-16y<br>53.5% male	Anxiety	Mental Health Test (Chinese) (>65)	1340	700	<u>Mean:</u><br>LBC: 114 vs. controls: 41<br><br><u>Prevalence:</u><br>LBC: 34.5% vs. controls: 14.5%; χ^2 11.2
Wang 2006† *China* [674]	CS	1 or both parents are internal labour migrants; duration NR<br><br>Mean (sd) age 10.53y (1.57)<br>53.4% male	Anxiety	SASC (>9)	491	393	OR (95% CI) 1.53 (1.05–2.27)
Wang 2014† *China* [65]	CS	1 or both parents are internal labour migrants; duration NR<br><br>Ages NR (primary school students)<br>Gender distribution NR	Depression<br>Anxiety	SCL-90 (mean)	952	952	<u>Depression: mean</u><br>LBC: 1.78 vs. controls: 1.67; F=1.009<br><br><u>Anxiety: mean</u><br>LBC: 1.74 vs. controls: 1.62; F=5.322<br><br>LBC had higher levels of depression and anxiety than non-LBC
Wei 2007† *China* [66]	CS	1 or both parents are internal labour migrants; duration NR	Depression<br>Anxiety	SCL-90 (mean)	248	218	<u>Depression: mean</u><br>Both parents absent: 1.1<br>1 parent absent: 1.2

**Study** *Country*	**Design**	**Definition of LBC and type of migration**	**Outcome**	**Instrument (cut-off)**	**No. of LBC**	**No. of controls**	**Effect estimate (95% CI)**
		Mean age 16y<br>44.6% male					Controls: 1.2<br><br><u>Anxiety: mean</u><br>Both parents absent: 1.4<br>One parent absent: 1.1<br>Controls: 1.1<br><br>Children left behind by both parents had higher levels of anxiety than children left behind by one parent and controls. No significant difference between children left behind by one parent and controls.
Wickramage 2015[†]<br>*Sri Lanka* [67]	CS	1 or both parents are international labour migrants; min. duration NR<br><br>1-17y (mental health outcomes assessed for ages 6-17y)<br>52% male	Conduct disorder	SDQ	110	113	<u>Proportion:</u><br>LBC: 39.3% vs. controls: 31.3%; χ^2 3.75, p=0.05
Wu 2015[†]<br>*China* [68]	CS	1 or both parents are internal labour migrants; duration NR<br><br>8-17y<br>39.9% male	Depression	CES-DC (mean)	466	158	<u>Mean:</u><br>Previously left-behind: 41.72<br>Currently left-behind: 44.13<br>Controls: : 41.95 (9.39)<br>Compared with controls, children who were currently left-behind had significantly higher levels of depression

Study *Country*	Design	Definition of LBC and type of migration	Outcome	Instrument (cut-off)	No. of LBC	No. of controls	Effect estimate (95% CI)
							(p<0.05). Children currently living with both parents but previously left-behind had significantly lower levels of depression than controls (p<0.01).
Wu 2016† *China* [69]	Cohort	1 or both parents are internal labour migrants; duration NR<br><br>7-16y<br>Gender NR	Anxiety	SASC<br>(>9)	476	340	T1: LBC: 57.8% vs. controls: 42.2%<br>T2: LBC: 62.1% vs. controls: 37.9%<br>T3: LBC: 59.0% vs. controls: 41.0%
Xie 2011† *China* [70]	CS	1 or both parents are internal labour migrants; duration NR<br><br>Ages NR (middle school and high school students)<br>51.0% males	Depression<br><br>Anxiety	Mental Health Inventory of Middle-school Students (mean)	1108	500	Depression: mean<br>LBC: 2.27 vs. controls: 2.14<br><br>Anxiety: mean<br>LBC: 2.39 vs. controls: 2.27<br><br>LBC had higher levels of depression and anxiety than non-LBC. No significance testing reported
Xu 2015 *China* [71]	Cohort	1 or both parents are internal labour migrants; duration NR<br><br>10-15y<br>49.3% male	Depression	Factor analysis score of 6 items of CES-D	256	1624	Mean (se):<br>LBC: 0.09 vs. controls: –0.01 (se 0.08); NS

**Study** *Country*	**Design**	**Definition of LBC and type of migration**	**Outcome**	**Instrument (cut-off)**	**No. of LBC**	**No. of controls**	**Effect estimate (95% CI)**
Yang 2010† *China* [72]	CS	1 or both parents are internal labour migrants; duration NR<br><br>8-15y; mean (sd) age 12.10y (2.19)<br>47.2% male	Depression	CDI (>19)	416	525	Proportion:<br>LBC 15.8% (male 19.6%; female 5.3%)<br>Controls 10.9% (male 15.0%; female 7.9%)<br><br>LBC significantly more likely to be depressed than controls
Yang 2011† *China* [73]	CS	1 or both parents are internal labour migrants; duration NR<br><br>10-16y<br>Gender distribution NR	Depression<br><br>Anxiety	SCL-90 (mean)	235	190	Depression: mean:<br>Primary school: LBC 1.32 vs. controls: 1.31; NS<br>Middle school: LBC: 1.59 vs. controls 1.57; significant<br><br>Anxiety mean:<br>Primary school: LBC: 1.47 vs. controls: 1.27; significant<br>Middle school: LBC: 1.43 vs. controls: 1.22; significant<br><br>No significance testing reported
Yao 2010† *China* [74]	CS	1 or both parents are internal labour migrants; min. duration 6m<br><br>10-18y<br>Gender distribution NR	Anxiety	MHT (Chinese) (NR)	682	2739	Proportion:<br>LBC: 8.50% vs. controls: 5.91%; χ^2 6.08<br><br>LBC were more likely to have anxiety symptoms than non-LBC. No significance testing

Study *Country*	Design	Definition of LBC and type of migration	Outcome	Instrument (cut-off)	No. of LBC	No. of controls	Effect estimate (95% CI)
							reported
Zhang 2011† *China* [75]	CS	1 or both parents are internal labour migrants; min. duration 12m<br><br>Mean (sd) age 14.23y (1.04)<br>Gender distribution NR	Conduct disorder	Child Behaviour Checklist (NR)	191	672	Mean:<br>LBC:45.79 vs. controls: 37.44<br><br>Proportion:<br>LBC: 59.7% vs. controls: 50.0%; χ^2 5.59, t=3.809
Zhang 2012† *China* [76]	CS	1 or both parents are internal labour migrants; duration NR<br><br>Ages NR (Years 3 to 6 at primary school)<br>Gender distribution NR	Anxiety	SASC	95	93	Mean:<br>LBC: 1.68 vs. controls: 1.51; t = 3.41<br><br>LBC had higher levels of anxiety than controls. No significance testing reported
Zhang 2013† *China* [77]	CS	1 or both parents are internal labour migrants; duration NR<br><br>Ages NR (Years 4 to 9 at primary school)<br>48.2% male	Anxiety	Mental Health Test (>65)	400	282	Mean:<br>LBC: 44.87 vs. controls: 42.45<br><br>Proportion:<br>LBC: 3.8% vs. controls: 2.8%; χ^2 1.66, F=0.36<br><br>LBC had significantly higher levels of anxiety than controls, though no significant difference was seen in terms of prevalence

Study<br>*Country*	Design	Definition of LBC and type of migration	Outcome	Instrument (cut-off)	No. of LBC	No. of controls	Effect estimate (95% CI)
Zhao 2010[†]<br>*China* [78]	CS	1 or both parents are internal labour migrants; duration NR<br><br>10-17; mean 14.03y<br>59.8% male	Depression	CDI (mean)	222	188	Mean:<br>1 parent absent: 14.10; B=-0.13<br>2 parents absent: 16.13; B=0.04<br>Controls: 15.96<br><br>Children left behind by one parent had lower levels of depression than children left behind by both parents and controls.
Zhao 2012[†]<br>*China* [79]	CS	1 or both parents are internal labour migrants; min. duration 6m<br><br>5-16y; mean 10.05y<br>51.2% male	Anxiety	SASC (mean)	926	1091	Mean:<br>LBC: 6.11 vs. controls: 5.84; p=0.09
Zhao 2014[†]<br>*China* [80]	CS	1 or both parents are internal labour migrants; duration NR<br><br>7-17y; mean (sd) 12.52y (2.20)<br>52.6% male	Anxiety	SASC (mean)	1694	1223	Mean (sd):<br>LBC: 5.99 (3.78) vs. controls: 5.45 (3.62); t = 3.90; p<0.001
Zhao 2017[†]<br>*China* [81]	CS	1 or both parents are internal labour migrants; duration NR<br><br>9-17y; mean (sd) 12.4y (2.1)<br>47.5% male	Conduct disorder	SDQ (mean)	2837	701	Mean:<br>Current LBC: 2.3 (1.5)<br>Previous LBC: 2.4 (1.6)<br>Controls: 2.2 (1.5)

Study *Country*	Design	Definition of LBC and type of migration	Outcome	Instrument (cut-off)	No. of LBC	No. of controls	Effect estimate (95% CI)
Zhou 2009† *China* [82]	CS	1 or both parents are internal labour migrants; min. duration 6m<br><br>6-16y; mean (sd) 10.71 (2.83); (depression and anxiety only among children in Year 3 or above)<br>49.7% males	Conduct disorder<br><br>Anxiety<br><br>Depression	Child Behaviour Checklist<br><br>Screen for Child Anxiety Related Disorders<br><br>Depression Self-Rating Scale for Children	607	873	Conduct disorder (internalising behaviour): mean<br>Father absent 6.25<br>Both parents absent 7.65<br>Controls 5.93<br>Children left-behind by both parents had more internalizing behaviours than non-LBC and children left-behind only by father<br><br>Conduct disorder (externalising behaviour): mean<br>No significant difference<br><br>Anxiety: mean<br>Both parents absent 20.99<br>Controls 18.42<br>Children left-behind by both parents had higher levels of anxiety than non-LBC<br><br>Depression: mean<br>Father absent 10.58<br>Mother absent 12.07<br>Both parents absent 11.36<br>Controls 10.56<br>Children left-behind by both parents or by mother only had higher levels of depression than controls

**Study**<br>***Country***	**Design**	**Definition of LBC and type of migration**	**Outcome**	**Instrument (cut-off)**	**No. of LBC**	**No. of controls**	**Effect estimate (95% CI)**
Zhou 2011†<br>*China* [83]	CS	1 or both parents are internal labour migrants; duration NR<br><br>8-17y; mean (sd) 12.3y (2.03)<br>52.0% male	Depression	CDI	486	1398	<u>Mean:</u><br>LBC: 13.13 vs. controls: 6.16<br><br><u>Proportion:</u><br>LBC: 19.8% vs. controls = 13.8%; F=5.05
Zhu 2012†<br>*China* [84]	CS	1 or both parents are internal labour migrants; min. duration 6m<br><br>6-16y<br>48.9% male	Conduct disorder	Child Behaviour Checklist (NR)	548	1017	<u>Prevalence:</u><br>LBC (6-11 yrs): 27.59%<br>LBC (12-16 yrs): 41.4%<br>LBC (all): 34.88%<br>Controls (6-11 yrs): 26.46%<br>Controls (12-16 yrs): 30.62%<br>Controls (all): 28.87%<br>p=0.06; χ^2 5.652

Table of study characteristics: nutrition outcomes

**Study**<br>***Country***	**Design**	**Definition of LBC and type of migration**	**Outcome**	**Instrument (cut-off)**	**No of LBC**	**No of controls**	**Effect estimate (95% CI)**
Ban 2017†<br>*China* [9]	CS	1 or both parents are internal labour migrants; duration NR<br><br>Under 3y<br>2010 survey: 53.1% male<br>2011 survey: 54.5% male	Stunting	WHO 2006 Child Growth Standards: HAZ<-2	2428	3708	Father absent only:<br>uOR 0.90 (0.67–1.21)<br>aOR 0.94 (0.77–1.16)<br><br>Mother absent (with/without father):<br>uOR 1.02 (0.83–1.27)<br>aOR 0.79 (0.61–1.03)

**Study** *Country*	**Design**	**Definition of LBC and type of migration**	**Outcome**	**Instrument (cut-off)**	**No of LBC**	**No of controls**	**Effect estimate (95% CI)**
Chen 2009 *China* [85]	CS	1 or both parents are internal labour migrants; duration NR<br><br>Under 18y<br>Gender distribution NR	Stunting<br>HAZ	WHO Child Growth Standards:<br>Stunting: HAZ <-2	NR	NR	LBC overall: B=0.370 (0.288);<br>2 parents absent, unadj.: B=0.677 (0.398) (significant);<br>2 parents absent, adj.: B=0.582 (0.398) (NS)
Chen & Wang 2010[†] *China* [86]	CS	1 or both parents are internal labour migrants; duration NR<br><br>Mean age 12.2y<br>52.1% male	HAZ<br>Stunting<br>BMIZ<br>Wasting<br>Overweight	WHO 2007 Child Growth Reference:<br>Stunting: HAZ <-2<br>Wasting: WHZ <-2<br>Overweight: BMIZ>1	1554	2462	HAZ mean: LBC -0.69 vs. controls -0.60<br>Stunting: LBC 6.7% vs. controls 6.4%<br>BMIZ mean: LBC -0.07 vs. controls 0.09<br>Wasting: LBC 0.2% vs. controls 1.3%<br>Significance NR
Chen 2011 *China* [87]	CS	Mother is internal labour migrant; duration NR<br><br>0-5y<br>Gender distribution NR	Stunting<br>Wasting	WHO 2006 Child Growth Standards:<br>HAZ<-2<br>WAZ<-2	NR	NR	% stunting (all children <5 years):<br>Rural average 17.8; General rural 12.5; Poor rural 22.9<br><br>% stunting (infants <12 months):<br>Rural average 7.4; General rural 3.3; Poor rural 15.9<br><br>% wasting (all children <5 years):<br>Rural average 6.8; General rural 5.9;<br>Poor rural 7.6

**Study** *Country*	**Design**	**Definition of LBC and type of migration**	**Outcome**	**Instrument (cut-off)**	**No of LBC**	**No of controls**	**Effect estimate (95% CI)**
							% wasting (infants <12 months):<br>Rural average 5.2; General rural 5.4; Poor rural 4.6
Chen 2012† *China* [88]	CS	1 or both parents are internal labour migrants; duration NR<br><br>Mean (sd) age 10.22y (1.68)<br>54.9% male	Stunting<br>HAZ<br>WAZ	Nutrition Assessment Standards for Chinese School-age Children (1985)	362	148	HAZ: F = 6.424<br>1-parent LBC had lower HAZ than 2-parent LBCs and controls<br><br>WAZ: F = 8.169<br>Both LBC groups had significantly lower WAZ than controls
Chen 2013 *China* [89]	CC	1 or both parents are internal labour migrants; duration NR<br><br>Under 7y<br>Gender distribution NR	Stunting<br>HAZ<br>Underweight<br>WAZ<br>Wasting<br>IDA	WHO 2006 Child Growth Standards:<br>Stunting: HAZ <-2<br>Underweight: WAZ<-2<br>Wasting: WHZ <-2<br>IDA (not defined)	1157	1157	HAZ: t=2.74; LBC had lower HAZ than controls<br>Stunting: χ^2 2.032 (non-significant)<br>Underweight: t=0.94 (non-significant)<br>WAZ: χ^2 0.147 (non-significant)<br>Wasting: χ^2 0.042 (non-significant)<br>IDA: χ^2 0.295 (non-significant)
Davis 2016 *Guatemala* [90]	CS	Father is international labour migrant; duration NR<br><br>0-35 months, mean 1.47y	HAZ<br>WHZ<br>WAZ	WHO 2006 Child Growth Standards	171	3802	HAZ: -0.427 (SE 0.176) $p \leq 0.05$<br><br>WHZ and WAZ: results

Study *Country*	Design	Definition of LBC and type of migration	Outcome	Instrument (cut-off)	No of LBC	No of controls	Effect estimate (95% CI)
		51.2% male					reported as non-significant only
Feng 2010† *China* [91]	CS	1 or both parents are internal labour migrants; min. duration 6m<br><br>0–7y<br>51.9% males	Stunting<br>Underweight<br>IDA	Chinese Children's Growth Standard Metrics<br><br>Hb<110g/L	1132	1095	Stunting: LBC 43.8% vs. controls 45.6%<br>Underweight: LBC 30.2% vs. controls 25.6%<br>IDA: LBC 17.5% vs. controls 14.9%<br><br>No significance reported
Frank 2005 *Mexico* [92]	CS	Father is international labour migrant to USA; duration NR<br><br>Newborn<br>Gender distribution NR	LBW	WHO cut-off: birthweight <2500g	90	475	χ^2 0.30 (p>0.1)<br>aOR 0.647 (p>0.05)
Gao 2010† *China* [13]	CS	1 or both parents are internal labour migrants; min. duration 6m<br><br>10-18y, mean 14.2y, median 14y<br>51.4% male	Overweight<br>Obesity<br>Stunting	Growth reference NR<br><br>Overweight & obesity: Chinese BMI-for-age cut-off points<br><br>Stunting: HAZ<-2	541	2445	**Overweight:**<br><u>Males:</u><br>aOR 1.76 (0.96-3.25) p=0.069<br>uOR 2.17 (1.39-3.37) p<0.001<br><u>Females:</u> NR<br><br>**Obesity:** not significant (results NR)<br>**Stunting:** not significant (results NR)
Graham 2013† *Vietnam, Philippines* [93]	CS	1 parent is international labour migrant; duration NR<br><br>9–11y<br>Gender distribution NR	Stunting	2007 WHO Growth Reference: HAZ<-2	Vietnam: 255<br>Phil: 237	Vietnam: 227<br>Phil: 243	<u>Vietnam:</u><br>*Unadj. model:*<br>Father absent: 0.94 (0.47-1.85)<br>Mother absent: 0.58 (0.31-1.09)

Study<br>*Country*	Design	Definition of LBC and type of migration	Outcome	Instrument (cut-off)	No of LBC	No of controls	Effect estimate (95% CI)
							Both absent: 1.08 (0.50-2.35)<br><br>*Fully adjusted model:*<br>Father absent: 1.10 (0.37-3.31)<br>Mother absent: 0.88 (0.25-3.03)<br>Both parents absent: 1.17 (0.34, 3.98)<br><br><u>Philippines:</u><br>*Unadj. model:*<br>Father absent: 0.33 (0.17-0.61) $p<0.001$<br>Mother absent: 0.36 (0.12-1.07)<br>Both absent: 0.41 (0.14-1.22)<br><br>*Fully adjusted model:*<br>Father absent: 0.59 (0.24-1.46)<br>Mother absent: 0.66 (0.10-4.52)<br>Both absent: 0.55 (0.10-3.17)
Li 2011[†]<br>*China* [94]	CS	1 or both parents are internal labour migrants; duration NR<br><br>3-6y<br>Gender distribution NR	Stunting<br>Underweight<br>IDA	US National Center for Health Statistics growth standards:<br>Stunting: HAZ<-2<br>Wasting	738	789	**Stunting:** $\chi^2 = 135.17$<br>LBC more likely to be stunted<br><br>**IDA:** $\chi^2 = 22.95$<br>LBC more likely to have anaemia

Study *Country*	Design	Definition of LBC and type of migration	Outcome	Instrument (cut-off)	No of LBC	No of controls	Effect estimate (95% CI)
				Underweight: WAZ<-2<br>Hb<110g/L			
Lu 2015<br>*Indonesia*, *Mexico* [95]	Cohort	1 or both parents are internal or international labour migrants<br><br>Indonesia: 0-15y; mean (sd) 8.5y (4.1)<br>51.0% male<br><br>Mexico: 0-12y; mean (sd) 8.2y (3.6)<br>50.9% male	HAZ<br>BMIZ	WHO 2006 Child Growth Standards & WHO 2007 Growth Reference	Indonesia:<br>Internal 178<br>Internat 101<br><br>Mexico:<br>Internal 283<br>Internat. 357	Indonesia: 3205<br><br>Mexico: 4606	Indonesia:<br>**HAZ:**<br>Internal: 0.402 (SE 0.133), p<0.01<br>Internat.: 0.096 (SE 0.601), p>0.05<br><br>**BMIZ:**<br>Internal: 0.262 (SE 0.141), p<0.1<br>Internat.: 0.079 (SE 0.081), p>0.05<br><br>Mexico<br>**HAZ:**<br>Internal: -0.044 (SE 0.099), p>0.05<br>Internat.: -0.205 (SE 0.083), p<0.05<br><br>**BMIZ:**<br>Internal: -0.042 (SE 0.089), p>0.05<br>Internat.: 0.058 (SE 0.073), p>0.05
Mo 2016[†]<br>*China* [96]	CS	1 or both parents are internal labour migrants; duration NR	Wasting, overweight,	2006 WHO Child Growth	269	466	**Wasting:**<br>Grandparent carer: OR 2.98

Study *Country*	Design	Definition of LBC and type of migration	Outcome	Instrument (cut-off)	No of LBC	No of controls	Effect estimate (95% CI)
		Under 6y<br>55.9% male	obesity	Standards:<br><br>**Wasting:** WHZ <-2<br><br>**Overweight:** BAZ (BMI-for-age z-scores) > 2			(1.43-6.19), p=0.003<br>Parent carer: OR 1.22 (0.71-2.74), p=0.853<br><br>**Overweight:**<br>Grandparent carer: 1.97 (1.17-3.31), p=0.011<br>Parent carer: 0.96 (0.54-3.22), p=0.675
Mou 2009† *China* [97]	CC	1 or both parents are internal labour migrants; min. duration 6,<br><br>Mean (sd) age 3.64y (1.87)<br>56.7% males	Stunting<br>Underweight<br>Wasting	WHO 2006 Child Growth Standards<br>Stunting: HAZ<-2<br>Underweight: WAZ<-2<br>Wasting: WHZ <-2	7585	7557	**Stunting:**<br>1 parent absent: 14.9%<br>2 parents absent: 17.9%<br>Controls: 16.3%<br>χ^2 =12.445<br>2-parent LBCs more likely to be stunted than 1-parent LBCs. No significant difference with the control group.<br><br>**Underweight:**<br>1 parent absent: 7.2%<br>Both parents absent: 8.3%<br>Control mean: 7.6%<br>χ^2 =3.050 (non-significant)<br><br>**Wasting**<br>1 parent absent: 3.1%<br>Both parents absent: 3.4%

Study<br>*Country*	Design	Definition of LBC and type of migration	Outcome	Instrument (cut-off)	No of LBC	No of controls	Effect estimate (95% CI)
							Control: 3.3%<br>χ^2 =0.521 (non-significant)
Mu 2015<br>*China* [98]	Cohort	Both parents are internal labour migrants; mean absence 14m<br><br>1997: 0-5y<br>2000: 0-8y<br>2004: 0-9y<br>2006: 2-7y<br>Gender distribution NR	Change in scores between surveys:<br>HAZ<br>WAZ<br>BMIZ	WHO 2006 Child Growth Standards & WHO 2007 Growth Reference	**2006:**<br>Total=85<br>1 (any) parent migrant: 29<br>Father migrant: 11<br>Mother migrant: 4<br>Both parents migrants: 13	**2006:**<br>56	**Change in HAZ**<br>0.058 (SE 0.736), p>0.05<br><br>**Change in WAZ:**<br>0.190 (SE 0.092), p<0.05<br><br>**Change in BMIZ:**<br>0.105 (SE 0.074), p>0.05
Nguyen 2016<br>*Ethiopia, India, Peru, Vietnam* [99]	Cohort	1 or both parents are labour migrants; duration ca. 1y<br><br>5-8y<br>Gender distribution NR	Underweight<br>WAZ<br>Stunting<br>HAZ<br>Overweight<br>Obesity	WHO 2007 growth reference<br><br>Underweight: WAZ<-2<br>Stunting: HAZ<-2<br>Overweight: BMIZ>1<br>Obesity : BMIZ>2	Ethiopia:<br>Mother absent: 35 (2007), 39 (2009)<br>Father absent: 135 (2007), 109 (2009)<br><br>India:<br>Mother absent: 10 (2007), 29 (2009)<br>Father absent: 52 (2007), 71(2009)<br><br>Peru:<br>Mother absent: 32 (2007),	Ethiopia:<br>1811 (2007). 1758 (2009) see mother daily; 1543 (2007). 1536 (2009) see father daily<br><br>India:<br>1879 (2007), 1778 (2009) see mother daily; 1786 (2007), 1651 (2009) see father daily<br><br>Peru:	Ethiopia:<br>Underweight: 0.0486<br>WAZ (continuous): -0.0368<br>Stunting: 0.0236<br>HAZ (continuous): -0.0253<br>Obesity: 0.0225<br>Overweight: -0.0035<br>None significant at 5%<br><br>India:<br>Underweight: -0.0671<br>WAZ (continuous): 0.1265<br>Stunting: -0.0159<br>HAZ (continuous): 0.3391<br>Obesity: 0.0005<br>Overweight: 0.427<br>None significant at 5%<br><br>Peru:

Study<br>*Country*	Design	Definition of LBC and type of migration	Outcome	Instrument (cut-off)	No of LBC	No of controls	Effect estimate (95% CI)
					45 (2009)<br>Father absent: 128 (2007), 163 (2009)<br><br>Vietnam:<br>Mother absent: 51 (2007),<br>52 (2009)<br>Father absent: 86 (2007),<br>67 (2009)<br><br>Note: groups not mutually exclusive	1886 (2007), 1841 (2009) see mother daily; 1586 (2007), 1510 (2009) see father daily<br><br>Vietnam:<br>1862 (2007), 1837 (2009) see mother daily; 1693 (2007), 1697 (2009) see father daily<br><br>Note: groups not mutually exclusive	Underweight: 0.0124<br>WAZ (continuous): -0.0659<br>Stunting: 0.0792 (significant at 5%)<br>HAZ (continuous): -0.1129<br>Obesity: -0.0276<br>Overweight: -0.0014<br><br>Vietnam:<br>Underweight: -0.0230<br>WAZ (continuous): 0.0233<br>Stunting: -0.0070<br>HAZ (continuous): -0.1614<br>Obesity: 0.0150<br>Overweight: 0.0256<br>None significant at 5%
Onyango 1994†<br>*Kenya* [100]	CS	Father is internal labour migrant; min. duration 6m<br><br>12-36m<br>Gender distribution NR	Stunting<br>Underweight<br>Wasting	NCHS reference (1978)<br>Stunting: HAZ <-2<br>Underweight WAZ <-2<br>Wasting: WHZ < -2	69	85	Stunting: LBC 47.8% vs. controls 38.8% (non-significant)<br><br>Underweight: LBC 18.8% vs. controls 28.8 % (non-significant)<br><br>Wasting: LBC 2.9% vs. controls 0% (non-significant)
Pan 2014†	CS	1 or both parents are internal labour	Stunting	WHO 2006	874	460	Stunting: χ^2 15.00; LBC more

Study *Country*	Design	Definition of LBC and type of migration	Outcome	Instrument (cut-off)	No of LBC	No of controls	Effect estimate (95% CI)
China [101]		migrants; min. duration 6m<br><br>10-14y, mean (sd) 11.63 (0.89)<br>49.6% males	HAZ<br>Wasting<br>Overweight	Child Growth Standards & WHO 2007 Child Growth Reference<br>Stunting: HAZ<-2<br>Wasting: BMIZ <-2<br>Overweight: BMIZ>1			likely to be stunted<br><br>HAZ: F=4.656; LBC have lower HAZ<br><br>Wasting: χ^2 =11.09; no significant differences between LBC and controls<br><br>Overweight: χ^2 =11.70; Children left behind by mother had higher overweight prevalence than controls. No significant differences between other types of LBC and controls.
Schmeer 2013 *Mexico* [102]	Cohort	Father is international labour migrant; min. duration 4y<br><br>3-12y<br>50% male	IDA	WHO cut-offs: Hb<11g/dL (age <5y)<br>Hb<11.5g/dL (5-12y)	Father current migrant: 93<br><br>Father previous migrant: 140	2882	**uOR:**<br>Father current migrant: 0.86 (0.31)<br>Father previous migrant: 0.58 (1.21)<br><br>**aOR (fully adjusted model):**<br>Father current migrant: 0.82 (-0.41)<br>Father previous migrant: 0.56 (1.30)<br><br>None significant at p<0.5
Tao 2016[†]	CS	1 or both parents are internal labour	Stunting:	Chinese	472	355	Stunting: higher prevalence

Study *Country*	Design	Definition of LBC and type of migration	Outcome	Instrument (cut-off)	No of LBC	No of controls	Effect estimate (95% CI)
China [56]		migrants; min. duration 6m<br><br>7-15y<br>51.1% male	'Abnormal development'<br><br>Wasting: 'emaciation'<br><br>Overweight<br>Obesity	National Health and Family Planning Commission (NHFPC) reference<br><br>Stunting: Chinese height-for-age Z scores<br><br>Wasting: Chinese BMI-for-age cut-offs<br><br>Overweight & obesity: Chinese BMI-for-age cut-offs			among LBC $p<0.05$<br><br>Wasting: higher prevalence among female LBC ($p<0.05$), no difference in males ($p>0.05$)<br><br>Overweight: no difference in females ($p>0.05$); lower prevalence among male LBC ($p<0.05$)<br><br>Obesity: no difference in females ($p>0.05$); lower prevalence among male LBC ($p<0.05$) as determined by χ^2 -test at $\alpha=0.05$
Wang & He 2011[†]<br>*China* [103]	CS	1 or both parents are internal labour migrants; min. duration 6m<br><br>Mean (sd) age 11.1y (1.0)<br>Gender distribution NR	Stunting<br>HAZ<br>WAZ<br>Overweight<br>Obesity<br>Wasting	WHO 2007 Child Growth Reference<br>Stunting: HAZ <-2<br>Wasting: WHZ < -2<br>Overweight: BMIZ>1<br>Obesity: BMIZ>2	590	285	Stunting: LBC 4.2% vs. controls 4.2%<br>Wasting: LBC 4.4% vs. controls 3.2%<br>Overweight: LBC 9.2% vs. controls 13.0%<br>Obesity: LBC 2.9% vs. controls 3.5%<br>No significant differences in stunting, wasting, overweight, obesity

**Study** *Country*	**Design**	**Definition of LBC and type of migration**	**Outcome**	**Instrument (cut-off)**	**No of LBC**	**No of controls**	**Effect estimate (95% CI)**
							WAZ:<br>LBC -0.98 vs. controls -0.70<br>LBC had significantly lower WAZ than controls
Wen 2008† *China* [104]	CS	1 or both parents are internal labour migrants; duration NR<br><br>6-17y, mean (sd) 11.14y (2.85)<br>49.6% males	Undernutrition (not defined)<br><br>Underweight<br>Overweight<br>Obesity	Nutrition Assessment Standards for Chinese School-age Children (1985); cut-offs NR	547	551	**Undernutrition:** χ^2 5.65 (no clear interpretation provided)<br><br>**Underweight:** χ^2 9.57 (no clear interpretation provided)<br><br>**Overweight:** χ^2 1.77; no significant differences between groups<br><br>**Obesity:** χ^2 0.965; no significant differences between groups
Wickramage 2015† *Sri Lanka* [67]	CS	1 or both parents are international labour migrants; min. duration NR<br><br>1-17y (228 aged <5y; 592 aged 12-17y)<br>52% male	Overweight<br>Underweight	Growth reference NR<br><br>Overweight: WHZ>+2 & ≤+3 sd<br><br>Underweight: WAZ<-2 to ≥-3	110	113	Group difference: χ^2 2.28 (df=4), p=0.061
Xia 2011† *China* [105]	CS	1 or both parents are internal labour migrants; min. duration 6m<br><br>Ages NR; year 4 & 7 students	Stunting<br>Wasting	WHO 2006 Child Growth Standards	1096	757	Stunting: LBC 24.2% vs. controls 18.2%<br><br>Wasting: LBC 27.9% vs.

Study *Country*	Design	Definition of LBC and type of migration	Outcome	Instrument (cut-off)	No of LBC	No of controls	Effect estimate (95% CI)
		50.8% males					controls 23.6%<br><br>Significantly higher prevalence of stunting and wasting among LBC
Xie 2013† *China* [106]	CS	1 or both parents are internal labour migrants; duration NR<br><br>Mean age (sd) 9.33y (4.17)<br>50.7% males	Stunting<br>Wasting	WHO 2007 Child Growth Reference<br>Stunting: HAZ <-2<br>Underweight: WAZ<-2	675	441	Stunting:<br>LBC 31.4% vs. controls 30.1%<br>LBC more likely to be stunted (significance NR)<br><br>WAZ:<br>LBC 8.2% vs. controls 6.4%<br>LBC more likely to be wasted (significance NR)
Yan 2013† *China* [107]	CS	1 or both parents are internal labour migrants; min. duration 6m<br><br>15-16y<br>Gender distribution NR	Wasting<br>Overweight<br>Obesity	Chinese School Aged Children BMI Screening Standard	442	195	LBC more likely to be wasted, less likely to be overweight and more likely to be obese) than controls. No effect estimates reported.
Yu 2013† *China* [108]	CS	Mother is internal labour migrant duration NR<br><br><18m<br>56.5% males	Malnutrition (defined as stunting or underweight**)**	WHO 2006 Child Growth Standards<br><br>Stunting: HAZ<-2<br>Underweight: WAZ<-2	200	1961	OR (95% CI) 1. 60 (1.09-2.37)
Zhou 2015 *China* [109]	CS	1 or both parents are internal labour migrants	WAZ<br>HAZ	Unclear which growth	NR	NR	HAZ mean: LBC -0.89 vs. controls -1.01

**Study** ***Country***	**Design**	**Definition of LBC and type of migration**	**Outcome**	**Instrument (cut-off)**	**No of LBC**	**No of controls**	**Effect estimate (95% CI)**
		3-17y Gender distribution NR	IDA	reference used <br>Hb<120g/L			WAZ mean: LBC -0.57 vs. controls -0.59 <br>IDA: LBC 27% vs. controls 27%

Table of study characteristics: substance use

Study *Country*	Design	Definition of LBC and type of migration	Outcome	Instrument & cut-off	No of LBC	No of controls	Effect estimate (95% CI)
Gao 2010† *China* [13]	CS	1 or both parents are internal labour migrants; min. duration 6m<br><br>10-18y, mean (sd) 14.2 (1.4), median 14.0<br>51.4 % male	Smoking (ever & current)<br><br>Alcohol use (ever, binge, current)	Adapted CYRBS and GSHS	541	2445	**Tobacco use: Ever smoked**<br>Males uOR: 1.20 (0.93-1.55); p=0.167<br>Males aOR:1.39 (1.02-1.90); p= 0.037<br>Females uOR 1.46 (0.98-2.16); p=0.060<br>Females aOR 1.88 (1.19-2.96); p=0.007<br><br>**Tobacco use: currently smoking**<br>Males uOR: 1.31 (0.96-1.80); p=0.093<br>Males aOR: 1.37 (0.92-2.05); p=0.118<br>Females uOR: 6.07 (2.02-18.24); p=0.002<br>Females adj OR : 6.58 (2.00-21.69); p=0.002<br><br>**Alcohol use: Ever drank**<br>Males: non-significant (results NR)<br>Females uOR 0.96 (0.72-1.28) p=0.786<br>Females aOR 1.35 (0.97-1.87) p=0.078<br><br>**Alcohol use: Binge drinking**<br>Males: non-significant (results NR)<br>Females uOR 2.74 (1.49-5.02) p=0.001<br>Females aOR 2.64 (1.30-5.37) p=0.007<br><br>**Alcohol use: currently drank**<br>Males: non-significant (results NR)<br>Females: non-significant (results NR)

Study *Country*	Design	Definition of LBC and type of migration	Outcome	Instrument & cut-off	No of LBC	No of controls	Effect estimate (95% CI)
Gao 2013† *China* [110]	CS	1 or both parents are internal labour migrants; min. duration 6m<br><br>11-19y<br>55.0% males	Smoking (past 30 days)	Self-report	Parent that ever migrated: 773<br><br>Parent currently migrated: 281	1504	OR (95% CI):<br><br>Boys:<br>Father migrant 0.54 (0.29–1.00), p=0.05<br>Mother migrant 1.91 (0.81–4.51), p=0.14<br>Both migrants 0.86 (0.43–1.73), p=0.67<br><br>Girls:<br>Father migrant 0.87 (0.16–4.79), p=0.87<br>Mother migrant 4.50 (0.46–43.66), p=0.19<br>Both migrants 1.15 (0.12–10.76), p=0.90
Guo 2012† *China* [34]	CS	1 or both parents are internal labour migrants; min. absence 6m<br><br>8-17y, mean (sd) 12.56 (1.83)<br>48.0% males	Smoking<br><br>Alcohol	Self-report	1143	1287	Smoking<br>LBC: 1.1% vs. controls: 1.8%; χ^2 3.44; p=0.179<br><br>Alcohol<br>LBC: 0.6% vs. controls: 0.9%; χ^2 0.82; p=0.663
Jiang 2015† *China* [111]	CS	1 or both parents are internal labour migrants; min. duration 6m<br><br>10-14y<br>56.0% males	Alcohol use (current)	Self-report	781	586	OR (95% CI) (vs. non-LBC controls):<br>2-parent LBCs: 1.91 (1.27-2.81); p<0.002<br>Father-only migrant 0.90 (0.52-1.55); p=0.704<br>Mother-only migrant: 1.16 (0.55-2.45); p=0.696
Jin & Qu	CS	1 or both parents are internal	Smoking (ever	Self-report	1405	2191	Mean cigarettes smoked:

Study<br>*Country*	Design	Definition of LBC and type of migration	Outcome	Instrument & cut-off	No of LBC	No of controls	Effect estimate (95% CI)
2009<br>*China*<br>[112]		migrants; duration NR<br><br>Year 4 to 9 students (ages NR)<br>51.2% males	smoked)				Father absent 1.20; Mother absent 1.22; Both absent 1.23; Control 1.23; F=0.317.<br>No significant difference between LBC and controls
Jordan 2013†<br>*Vietnam*<br>[113]	CS	1 or both parents are international labour migrants; min. duration 6m<br><br>41.97% aged 9y; 30.92% aged 10y; 27.11% aged 11y<br>45.8% male	Alcohol use (ever)	Self-report	Father migrant: 43<br><br>Mother migrant: 73<br><br>2 parents migrants: 40	219	Father migrant: OR 1.09 (0.52-2.29)<br>Mother migrant: OR 0.53 (0.26–1.07), $p<0.1$<br>2 parents migrants: OR 0.39 (0.15–1.04), $p<0.1$<br><br>No significant impact of parental migration on alcohol use in LBC
Li 2012†<br>*China*<br>[114]	CS	1 or both parents are internal labour migrants; duration NR<br><br>Years 7-9 students (<14y)<br>Gender distribution NR	Smoking, alcohol or internet use	Self-reported	810	1329	<u>Smoking:</u><br>Father absent 3.80%; Mother absent 8.22%; 2 parents absent; 7.19%; Controls 3.23%<br>Children left behind by mother were more likely to smoke than children left behind by father and controls<br><br><u>Alcohol:</u><br>Father absent 6.40%; Mother absent 6.85%; 2 parents absent 11.25%; Controls 7.67%<br>2-parent LBCs more likely to drink alcohol than children left behind by father and controls
Lin 2011†<br>*China*<br>[115]	CS	1 or both parents are internal labour migrants; duration NR	Smoking (ever and past 30 days)	Self report	416	525	<u>Ever smoked:</u><br>LBC 14.0% vs. 7.3%; χ^2 7.80

**Study** *Country*	**Design**	**Definition of LBC and type of migration**	**Outcome**	**Instrument & cut-off**	**No of LBC**	**No of controls**	**Effect estimate (95% CI)**
		Primary and junior middle school students (ages NR)<br>48.5% males					Past 30 days smoking:<br>LBC 9.5% vs. 5.8%; χ^2 3.32
Qu 2015† *China* [50]	CS	1 or both parents are internal labour migrants; min. duration 6m<br><br>6-16y<br>50.4% males	Substance use	MINI-KID (DSM-IV diagnostic criteria)	7331	12380	OR (LBC vs. controls):<br>2.59 (1.95–3.45); p=0.001
Shen 2015† *China* [52]	CS	1 or both parents are internal labour migrants; min. duration 6m<br><br>10-18y; mean (sd) 14.22 (1.71)<br>45% males	Smoking<br>Alcohol	Self-report	1397	886	Substance use<br>1 parent migrated: 4.0%<br>2 parents migrated: 6.6%<br>Controls: 4.1%<br>Non-significant difference
Sukamdi 2013† *Indonesia* [116]	CS	1 or both parents are migrants; duration NR<br><br>9-11y<br>49.7% males	Smoking (ever)	Self report	Father migrant; mother carer 73<br><br>Mother migrant; father carer 107<br><br>1 parent migrant; other carer 27	244	Controls: 7.79%<br>Father-migrant/mother-carer 8.22%<br>Mother-migrant/father-carer 12.15%<br>Parent-migrant /other carer 7.41%<br>Any migrant household: 10.14%<br><br>OR (95% CI):<br>Father-migrant/mother-carer: 0.65 (0.20–2.16)<br>Mother-migrant/father-carer: 1.65 (0.68–3.99)<br>Parent-migrant/other carer: 1.31 (0.23–7.35)
Wen 2012† *China* [10]	CS	1 or both parents are internal labour migrants; duration NR<br><br>8-18y	Smoking<br>Illicit drug use<br>Alcohol (binge in past month	Self-report	303	322	OR (95% CI)<br>2 parents migrants 0.78 (0.42–1.44)<br>Father migrant 0.56 (0.29–1.07), p<0.10

Study<br>*Country*	Design	Definition of LBC and type of migration	Outcome	Instrument & cut-off	No of LBC	No of controls	Effect estimate (95% CI)
		Gender distribution NR					Mother migrant 0.43 (0.19 – 0.97), p<0.05
Yang 2016†<br>*China*<br>[117]	CS	1 or both parents are internal labour migrants; min. duration 6m<br><br>10-14y<br>55.8% males	Smoking (past 30 days)	Self-report	757	586	uOR (95% CI):<br>Father migrant: 2.39 (0.91-6.26) p=0.076<br>Mother migrant: 3.80 (1.22-11.87) p=0.021<br>2 parents migrants 4.71 (2.08-10.64) p=0.000<br><br>aOR (95% CI):<br>Father migrant 3.20 (0.94-8.77) p=0.063<br>Mother migrant 5.60 (1.67-18.73) p=0.005<br>2 parents migrants 5.59 (2.38-13.15) p=0.000
Zhang 2015†<br>*China*<br>[118]	CS	1 or both parents are internal labour migrants; duration NR<br><br>Mean age (sd) 15.03y (1.76)<br>45.4% males	Smoking (past 30 days)<br>Alcohol (past 30 days)	Self-report	1336	1024	Parental migration was not associated with smoking behaviour, but LBC were more likely to be both smoking and drinking, compared to non-LBC, according to the adjusted analysis. No effect estimate reported

Table of study characteristics: infectious disease

Study<br>*Country*	Design	Definition of LBC and type of migration	Outcome	Instrument & cut-off	No of LBC	No of controls	Effect estimate (95% CI)
Carling 2013<br>*Malawi*	CS	Father is a migrant; duration NR	Diarrhoea	Self -report	Total 2281	Total 34178	Father migrant: 17.2%<br>Controls: 17.9%

**Study** *Country*	**Design**	**Definition of LBC and type of migration**	**Outcome**	**Instrument & cut-off**	**No of LBC**	**No of controls**	**Effect estimate (95% CI)**
[119]		0-14y (diarrhoea only assessed in 0-5y)<br>Gender distribution NR					OR 0.92 (non-significant)
Guo 2012[†]<br>*China* [34]	CS	1 or both parents are internal labour migrants; min. absence 6m<br><br>8-17y, mean (sd) 12.56y (1.83)<br>48% males	Diarrhoea in past 2 weeks	Self-report	1143	1287	LBC: 19.9% vs. controls 19.8%; χ^2 3.88; p=0.144
Schmeer 2009<br>*Mexico*<br>[120]	Cohort	Father is a labour or educational migrant; duration NR<br><br>0-5y<br>50% male	Diarrhoea	Self-report	Wave 1: 14481<br>Wave 2: 12 447<br>Wave 3: 11094<br>Wave 4: 8848	Unclear	<u>OR:</u><br>Father absent vs. father present: 1.51 (SE 0.12); p<0.01<br><br>Conditional logit model: 1.79 (SE 0.42); p<0.05
Wang & Zhang 2012<br>*China* [121]	CS	Both parents are internal labour migrants; duration NR<br><br><5y<br>49.6% males	Diarrhoea	Self-report	79	265	<u>OR (95% CI):</u><br>OR=2.29 (1.04-5.07); B= 0.829<br>LBCs more likely to have diarrhoea in the past two weeks in the adjusted model
Zhou 2015<br>*China* [109]	CS	1 or both parents are internal labour migrants; duration NR<br><br>3-17y<br>Gender distribution NR	Soil-transmitted helminth infection	Kato-Katz smear test (ascaris, whipworm and hookworm)	NR	NR	LBC 25%; controls 39%; p<0.001

Table of study characteristics: abuse

**Study** *Country*	**Design**	**Definition of LBC and type of migration**	**Outcome**	**Instrument & cut-off**	**No of LBC**	**No of controls**	**Effect estimate (95% CI)**
Asis 2013† *Philippines* [23]	CS	1 or both parents are international labour migrants; min. duration 1 year<br><br>10-12y; mean (sd) 10.7 (1.15)<br>46.0% male	Abuse	Self-report	Father absent: 628<br><br>Mother absent: 347<br><br>2 parents absent: 150	318	Physical abuse:<br>Father absent 39.4%; Mother absent 31.1%; Both absent 37.3%; Controls 42.2%<br><br>Neglect:<br>Father absent 9.6%; Mother absent 10.6%; Both absent 10.0%; Controls 11.3%<br><br>Significance not reported
Chen 2016† *China* [26]	CS	1 or both parents are internal labour migrants; duration NR<br><br>Grade 4-9 students; mean age (sd) 13y (1.71)<br>52% males	Physical, emotional and sexual violence	Adapted version of Juvenile Victimisation Questionnaire	443	245	OR (95% CI):<br>1-3 types of victimisation 1.27 (0.83-1.95)<br><br>≥4 types of victimisation 1.58 (0.98-2.57)<br><br>Any physical assault: 1.49 (1.09-2.05)<br><br>Any victimization in past year:<br>LBC 84.8% vs. controls 80.4% ; $p<0.05$
Liu & Zhong 2012 *China* [122]	CS	1 or both parents are internal labour migrants; duration NR<br><br>0-6y<br>53.8% males	Physical, emotional and sexual violence	Child Neglect Scale for Rural Chinese Children	862	626	Mean scores:<br>LBC 49.3 vs. controls 47.0<br><br>Proportion:<br>LBC 37.2% vs. controls 23.8%

**Study** *Country*	**Design**	**Definition of LBC and type of migration**	**Outcome**	**Instrument & cut-off**	**No of LBC**	**No of controls**	**Effect estimate (95% CI)**
							LBC were more prone to child neglect than controls
Pottinger 2005† *Jamaica* [48]	CC	1 or both parents are international labour migrants; min. duration 6m; 44% absent for >4y<br><br>9-10y<br>52% males	Violence and abuse	Self-report	27	27	No significant differences overall but parental migration was associated with community violence (r=0.39, p<0.05) and domestic violence (r=0.70, p<0.05)
Shen 2015† *China* [52]	CS	1 or both parents are internal labour migrants; min. duration 6m<br><br>10-18y; mean (sd) 14.22 (1.71)<br>45% males	Physical abuse	Self-report	1397	886	1 parent migrated: 9.3%<br>2 parents migrated: 12.3%<br>Controls: 8.9% (non-significant)
Wang 2008† *China* [123]	CS	1 or both parents are internal labour migrants; duration NR<br><br>11-16y<br>Gender distribution NR	Unsafe sex behaviours<br><br>Physical, emotional and sexual violence<br><br>Rape	Self-report	839	507	Unprotected sex<br>LBC 75.9% vs. control 63.5%; χ^2 1.365<br>A lower proportion of LBC correctly used contraceptive during sex.<br><br>Sexual abuse:<br>LBC 11.69% vs. control 11.68%; non-significant (effect estimate NR)<br><br>Rape:<br>LBC: 8.37% vs. control: 6.47%; χ^2 1.636 (non-significant)
Zhao 2014† *China* [80]	CS	1 or both parents are internal labour migrants; duration NR<br><br>7-11y; mean (sd) 12.52 (2.20)<br>52.6% males	Neglect and physical abuse	Parents-Child Conflicts Tactics Scale (Chinese version)	1694	1223	LBC had lower prevalence of physical abuse (v2=14.79, p<0.001) and higher prevalence of neglect (v2=14.32, p<0.001) compared to non-LBC

Table of study characteristics: unintentional injuries

**Study** *Country*	**Design**	**Definition of LBC and type of migration**	**Outcome**	**Instrument & cut-off**	**No of LBC**	**No of controls**	**Effect estimate (95% CI)**
Chen & Qu 2010† *China* [124]	CS	1 or both parents were internal labour migrants; min. interval between parents visiting home 3m<br><br>Year 3-9 students (ages NR)<br>52.0% males	Road traffic incidents	Self-report	1423	2303	Mean for LBC: 1.5%<br>Mean for Control: 2.2%<br>Significance NR; no effect estimate reported
Jiang 2011† *China* [125]	CS	1 or both parents are internal labour migrants; min. duration 6m<br><br>2-12y<br>56.6% males	Road traffic incidents in past year	Self-report	1096	1488	LBC were more likely to have any road traffic accident in the past year than non-LBC<br>Mean LBC: 10.4%<br>Mean Control: 8.2%<br>Reported as significant; no effect estimate reported
Shen 2009† *China* [126]	CS	1 or both parents are internal labour migrants; min. duration 6m<br><br>5-17y<br>76.3% males	Road traffic incidents<br><br>Drowning	Self-report	297	220	Road traffic incidents:<br>LBC 3.7% vs. controls 10.5%<br><br>Drowning:<br>LBC 5.1% vs. controls 4.1%<br><br>Annual injury rate per 1000 (95% CI):<br>LBC: 252.9 (233.0-273.0)<br>Controls: 119.8 (104.9-134.7)<br><br>Annual injury rate among LBC was more than double that among controls
Shen 2013† *China* [127]	CS	1 or both parents are internal labour migrants; min. duration 6m	Farm related injuries	Self-report	Father home 89	531	Incidence rate of injury:<br>No parents home 18.2 (14.9-21.6)<br>Father home 27.0 (17.7-36.2)

Study<br>*Country*	Design	Definition of LBC and type of migration	Outcome	Instrument & cut-off	No of LBC	No of controls	Effect estimate (95% CI)
		13-19y<br>59.7% males			Mother home 518<br><br>2 parents absent 505		Mother home 19.8 (16.7-22.9)<br>Controls 17.8% (14.9-20.8)<br><br>OR (95% CI):<br>Mother at home 1.08 (0.79-1.48)<br>Father at home 1.30 (0.65-2.63)<br>No parents at home 1.27 (1.00-1.69)
Zhao 2008†<br>*China* [128]	CS	1 or both parents are internal labour migrants; duration NR<br><br>Mean age (sd) 14.25y (1.77)<br>53.5% males	Road traffic incidents (in past year)	Self-report	1614	1908	LBC 11.15% vs. controls: 6.50%; χ^2 24.01 (reported as significant)<br>LBC were more likely to have any road traffic incident within the past year vs. controls

† Included in meta-analysis

Abbreviations: aOR adjusted odds ratios; BMI body mass index; BMIZ body mass index z-scores; CC case-control; CDI Children's Depression Inventory; CDI Children's Depression Inventory Short Form; CES-D Center for Epidemiologic Studies Depression Scale; CES-DC Center for Epidemiologic Studies Depression Scale in Chinese; CS cross-sectional; CYRBS Chinese Youth Risk Behavior Survey; DSRSC Depression Self-Rating Scale; F F-test; GSHS Global School-based Health Survey; HAZ height-for-age z-score; internatl international; IDA iron deficiency anaemia; JEPQ-R Junior Eysenck Personality Questionnaire Revised; LBC left-behind children; m months; MHT Mental Health Test; MINI-KID Mini-International Neuropsychiatric Interview for Children and Adolescents; OR odds ratio; SAS Self-rating Anxiety Scale; SASC Social Anxiety Scale for Children; SCL-90 Symptom Checklist-90; SCARED Screen for Child Anxiety Related Disorders; sd standard deviation; SDQ Strengths and Difficulties Questionnaire; STAIC State-Trait Anxiety Inventory for Children; SMFQ Short Mood and Feelings Questionnaire; uOR unadjusted odds ratio; WAZ weight-for-age z-score; WHZ weight-for-height z-score; y year; YSR Youth Self-Report; YRBS Youth Risk Behavior Survey; 95% CI 95% confidence interval; χ^2 Chi-squared tes

Table of excluded studies

Study ID	Authors	Title	Year	Journal	Vol	Issue	Pages	Languag e	Primary or secondar y	Reason for exclusion
Adam	Adam, S.;	The Effects of the Labour Force	2016	Social Inclusion and Equal			13-22	English	Primary	Exclude 3

2016	Whitehouse, S.	Migration on Children's Identity Formation. Case-study: Romania and United Kingdom		Opportunities (Sieo 2016)						
Adrian 2012	Adrian, J.; Andreea, B.	Aspects of the social protection for children left behind due to parental cross-border migration	2012	International Conference Social Work Perspectives on Quasi-Coercive Treatment of Offenders: Violence among Adolescents (Specto 2012), 3rd Edition			4	English	Primary	Exclude 7
Adriana 2016	Adriana, B.; Cristiana, G.; David-Rus, R.; Emilia, I.	Value changes in left-behind children of Romanian migrants	2016	Mental Health: Actual Views in Psychology, Medicine and Anthropology			20-24	English	Primary	Exclude 8
Ai 2016	Ai, H. S.; Hu, J. M.	Psychological resilience moderates the impact of social support on loneliness of "left-behind" children	2016	J. Health Psychol.	21	6	1066-1073	English	Primary	Exclude 6
Antai 2010	Antai, D.	Migration and child immunization in Nigeria: individual- and community-level contexts	2010	BMC Public Health	10		12	English	Primary	Exclude 6
Anton 2010	Anton, J. I.	The Impact of Remittances on Nutritional Status of Children in Ecuador	2010	Int. Migr. Rev.	44	2	269-299	English	Primary	Exclude 5
Azzarri 2011	Azzarri, C.; Zezza, A.	International migration and nutritional outcomes in Tajikistan	2011	Food Policy	36	1	54-70	English	Primary	Exclude 13 household level
Bakker 2009	Bakker C, Elings-Pels M, Reis M	The impact of migration on children in the Caribbean	2009	UNICEF report			https://www.unicef.org/easterncaribbean/Impact_of_Migration_Paper.pdf	English	Secondary	Exclude 7
Bennett 2015	Bennett, R.; Hosegood, V.;	An Approach to Measuring Dispersed Families with a Particular Focus on	2015	Popul. Space Place.	21	4	322-334	English	Primary	Exclude 6

	Newell, M. L.; McGrath, N.	Children 'Left Behind' by Migrant Parents: Findings from Rural South Africa								
Biao 2007	Biao, X.	How far are the left-behind left behind? A preliminary study in rural China	2007	Popul. Space Place.	13	3	179-191	English	Primary	Exclude 6
Boehm 2008	Boehm, D. A.	"For My Children:" Constructing Family and Navigating the State in the US-Mexico Transnation	2008	Anthropol. Q.	81	4	777-802	English	Primary	Exclude 5
Bohr 2010	Bohr, Y.	Transnational Infancy: A New Context for Attachment and the Need for Better Models	2010	Child Develop. Perspect.	4	3	189-196	English	Primary	Exclude 7
Bowen 2015	Bowen, L.; Taylor, A. E.; Sullivan, R.; Ebrahim, S.; Kinra, S.; Krishna, K. V. R.; Kulkarni, B.; Ben-Shlomo, Y.; Ekelund, U.; Wells, J. C. K.; Kuper, H.	Associations between diet, physical activity and body fat distribution: a cross sectional study in an Indian population	2015	BMC Public Health	15		12	English	Primary	Exclude 4
Brockerhoff 1998	Brockerhoff		1998					English	Secondary	Exclude 4
Bryant 2005	Bryant J	Children of international migrants in Indonesia, Thailand, and the Philippines: a review of evidence and policies	2005	UNICEF Innocenti Working Paper			https://www.unicef-irc.org/publications/pdf/iwp20	English	Secondary	Exclude 6
Cao 2011	Cao, Q., Wu, Z., Sun, Y., Wang, T.,	Application of negative binomial regression and modified Poisson regression in the research of risk	2011	Journal of hygiene research	40	6	702-704	Chinese	Primary	Exclude 6

	Han, T. and Gu, C	factors for injury frequency								
Cao 2011	CAO Jie-jie, CHEN Ting-ting, HE Nan, Zhang Zhi-gang, Cao Xiu-jing	Effects of early maternal separation on physical and psychological health in rural middle school students	2011	Chinese Journal of School Health	32	7	769-773	Chinese	Primary	Exclude 7
Carletto 2011	Carletto, C.; Covarrubias, K.; Maluccio, J. A.	Migration and child growth in rural Guatemala	2011	Food Policy	36	1	16-27	English	Primary	Exclude 13 household level
Cebotari 2017	Cebotari, V.; Mazzucato, V.; Siegel, M.	Child Development and Migrant Transnationalism: The Health of Children Who Stay Behind in Ghana and Nigeria	2017	J. Dev. Stud.	53	3	444-459	English	Primary	Exclude 6
Chen 2006	Chen, C.M., He, W. and Chang, S.Y.	The changes of the attributable factors of child growth	2006	Journal of hygiene research	35	6	765-768	Chinese	Primary	Exclude 8
Chen 2011	Chen, H.H., Hu, B.S., He, F.R., Liu, Q.L.	Influencing factors on psychological health between left-behind children and not left-behind children in junior middle school	2011	Chinese Journal of School Health		4	407-411	Chinese	Primary	Exclude 9
Chen 2014	Chen, S.H., Liao, Z.G., Wang, S.H., Xi, B.R., Liu, H., He, S.J.	Associated factors of behavioral problems among left-behind children in Jiangxi	2014	Chinese Journal of School Health	35	1	95-97	Chinese	Primary	Exclude 5
Chen SF 2016	Chen, S. F.; Tian, Y.; Yao, D. Z.	The Relationship between Attachment and Impact of Life Events of College Student with Left-behind Experience	2016	2016 2nd Bf International Conference on Psychology, Sports Science	5		42-46	English	Primary	Exclude 10

				and Social Sciences						
Chen Y 2016	Chen, Y.; Gao, Y.; Zhou, L.; Tan, Y. F.; Li, L. P.	A Comparative Study of Dog- and Cat-Induced Injury on Incidence and Risk Factors among Children	2016	Int. J. Environ. Res. Public Health	13	11	11	English	Primary	Exclude 6
Cheng 2012	Cheng, S., Zheng, H.F.; Pang, P.; Zhang, Y.K.; Hu, C.L.; Li, L.; Wang, S.F.; Su, P.Y.	Health status among left-behind children aged 1-4 years with both parents working out in rural Anhui province	2012	Chinese Journal of School Health	28	12	1583-1586	Chinese	Primary	Exclude 11
Choe 2004	Choe MK, Hatmadji SH, Podhista C, Raymundo CM, Thapa S	Substance use and premarital sex among adolescents in Indonesia, Nepal, the Philippines and Thailand	2004	Asia-Pacific Population Journal	19	1	http://www.unescapsdd.org/files/documents/PUB_APPJ-Vol-19-No-1.pdf	English	Secondary	Exclude 4
Collinson 2010	Collinson, M. A.	Striving against adversity: the dynamics of migration, health and poverty in rural South Africa	2010	Glob. Health Action	3		14	English	Primary	Exclude 3
Colvin 2000	Colvin, M.	Sexually transmitted infections in southern Africa: a public health crisis	2000	S. Afr. J. Sci.	96	6	335-339	English	Primary	Exclude 8
Cortes 2007	Cortes, P.	Children and women left behind in labor sending countries: an appraisal of social risks	2007	Report			http://www.childmigration.net/files/Rosalia_Cortes_07.pdf	English	Secondary	Exclude 6
Cortes 2015	Cortes, P.	The Feminization of International Migration and its Effects on the Children Left Behind: Evidence from the Philippines	2015	World Dev.	65		62-78	English	Primary	Exclude 6
Creighton	Creighton, M. J.; Goldman,	Migrant networks and pathways to child obesity in Mexico	2011	Soc. Sci. Med.	72	5	685-693	English	Primary	Exclude 13

2011	N.; Teruel, G.; Rubalcava, L.									household level
Dai 2017	Dai, Q.; Yang, G.; Hu, C.; Wang, L.; Liu, K.; Guang, Y.; Zhang, R.; Xu, S.; Lin, B.; Yang, Y.; Feng, Z.	The alienation of affection toward parents and influential factors in Chinese left-behind children	2017	Eur. Psychiat.	39		114-122	English	Primary	Exclude 6
Damon 2014	Damon, A.; Kristiansen, D.	Childhood obesity in Mexico: the effect of international migration	2014	Agric. Econ.	45	6	711-727	English	Primary	Exclude 13 household level
Dang 2010	Dang, Y. H.; Yao, M. L,	Investigation on the current situation of psychological and behavioral challenges among rural left-behind children	2010	Maternal and Child Health Care of China	25	11	1519-1520	Chinese	Primary	Exclude 6
de Snyder 1996	de Snyder, V. N. S.	Psychosocial problems in international migration	1996	Salud Ment.	19		53-59	English	Primary	Exclude 7
deBrauw 2011	de Brauw, A.	Migration and child development during the food price crisis in El Salvador	2011	Food Policy	36	1	28-40	English	Primary	Exclude 13 household level
deBrauw 2011	de Brauw, A.; Mu, R.	Migration and the overweight and underweight status of children in rural China	2011	Food Policy	36	1	88-100	English	Primary	Exclude 13 duplicate dataset
D'Emilio 2007	D'Emilio AL, Cordero B, Bainvel B, Skoog C,	The impact of international migration: children left behind in selected countries of Latin America and the Caribbean	2007	UNICEF report			https://www.unicef.org/socialpolicy/files/The_Impact_of_Internation	English	Secondary	Exclude 7

	Comini D, Gough J, Dias M, Saab R, Kilbane T						al_Migration_LAC.pdf			
Deng 2014	Deng, F.M., Gong, X.M., Cui, H.Y., Yang, Y.J. and Hu, P.C.	Risk factors for unintentional injury among children in rural areas of Liling	2014	Chinese journal of contemporary pediatrics	16	5	524-528	Chinese	Primary	Exclude 6
Dillon 2012	Dillon, M.; Walsh, C. A.	Left Behind: The Experiences of Children of the Caribbean Whose Parents Have Migrated	2012	J. Comp. Fam. Stud.	43	6	871-+	English	Primary	Exclude 7
Ding 2014	Ding, G. D.; Bao, Y. X.	Editorial Perspective: Assessing developmental risk in cultural context: the case of 'left behind' children in rural China	2014	J. Child Psychol. Psychiatry	55	4	411-412	English	Primary	Exclude 7
Donato 2011	Donato, K. M.; Duncan, E. M.	Migration, Social Networks, and Child Health in Mexican Families	2011	J. Marriage Fam.	73	4	713-728	English	Primary	Exclude 6
Dreby 2015	Dreby, J.	US immigration policy and family separation: The consequences for children's well-being	2015	Soc. Sci. Med.	132		245-251	English	Primary	Exclude 3
Duan 2009	Duan, D.H., Zhu, M.Y., Luo, J.Y., Wang, Z., Gu, C.H., Zhang, W.M., Yao, M.L. and Duan, G.Q	Investigation on dietary nutrients among rural stranded children of 2-7 year olds in china.	2009	Chinese Journal of Epidemiology	30	4	326-330	Chinese	Primary	Exclude 6
Duan 2014	Duan, B.J., Zhang, Y.J.	Mediation effects of self-concept between loneliness and psychological abuse and neglect among left-behind	2014	Chinese Journal of School Health	35	10	1551-1553	Chinese	Primary	Exclude 5

		children								
Duval 2010	Duval, L.; Wolff, F. C.	Remittances matter: longitudinal evidence from Albania	2010	Post-Communist Econ.	22	1	73-97	English	Primary	Exclude 5
Fan 2009	Fan, X.H., Fang, X.Y., Liu, Q.X. and Liu, Y.	A social adaptation comparison of migrant children, rear children, and ordinary children	2009	Journal of Beijing Normal University		5	33-40	Chinese	Primary	Exclude 6
Fan 2011	Fang, X.H.	On the Comparison of Emotional Adaptation Between Left-at-Home Rural Children of Various Types and Normal Children	2011	Chinese Journal of Special Education		2	71-77	Chinese	Primary	Exclude 6
Fan 2013	FAN Zhi-guang, WEI Xin, DU Ling-li, LI Ying	A study on aggressive behaviors of urban primary school left-behind children	2013	Modern Preventive Medicine	40	13	2426-2428	Chinese	Primary	Exclude 11
Fang 2009	Fang, Q., Wang, H., LI, L.L., LI, J.	Survey on the mental health status of middle-school students in the Three-Gorge Area, Chongqing	2009	Chinese Health Service Management		11	774-778	Chinese	Primary	Exclude 5
Fang 2013	Fang, Q., Wang, H. and LI, L.L.	Sub-health status and behavioral risk factors among middle school students in three gorges area, Chongqing.	2013	Chinese Journal of Public Health	29	1	94-96	Chinese	Primary	Exclude 5
Feng 2011	Feng, H.; Liu, J. H.; Wang, Y.; He, G. P.	Sociodemographic Correlates of Behavioral Problems Among Rural Chinese Schoolchildren	2011	Public Health Nurs.	28	4	297-307	English	Primary	Exclude 6
Fernando 1989			1989					English	Secondary	Exclude 8
Frank 2002	Frank, R.; Hummer, R. A.	The other side of the paradox: The risk of low birth weight among infants of migrant and nonmigrant households within Mexico	2002	Int. Migr. Rev.	36	3	746-765	English	Primary	Exclude 13 household level
Gao 2008	Gao, Y.	Research on mental health and personality traits of rural children	2008	Chinese Public Health	24	8	76-80	Chinese	Primary	Exclude 6

		without parental care								
Gibson 2008	Gibson, M. A.	Does investment in the sexes differ when fathers are absent? Sex-biased infant survival and child growth in rural Ethiopia	2008	Hum. Nat.-Interdiscip. Biosoc. Perspect.	19	3	263-276	English	Primary	Exclude 5
Gibson 2011	Gibson, J.; McKenzie, D.; Stillman, S.	What happens to diet and child health when migration splits households? Evidence from a migration lottery program	2011	Food Policy	36	1	42186	English	Primary	Exclude 5
Givaudan 2013	Givaudan, M.; Pick, S.	Children Left Behind: How to Mitigate the Effects and Facilitate Emotional and Psychosocial Development	2013	Child Abuse Negl.	37	12	1080-1090	English	Primary	Exclude 6
Givaudan 2016	Givaudan, M.; Barriga, M.; Kercheval, J.; Pick, S.	Children left behind by migration: training their caretakers	2016	Int. J. Migr. Health Soc. Care	12	3	170-184	English	Primary	Exclude 6
Goian 2010	Goian, C.; Vintila, M.	Migration as a cause of communication problems in adolescents	2010	12th Biennial Conferences of the European Association for Research on Adolescence			161-166	English	Primary	Exclude 10
Gong 2010	Gong, Z. and Yang, Q.	Analysis on nutrition status and its influencing factors of left-behind children in rural of Hubei	2010	Maternal and Child Health Care of China	25	26	3775-3778	Chinese	Primary	Exclude 6
Graham 2012	Graham, E.; Jordan, L. P.; Yeoh, B. S. A.; Lam, T.; Asis, M.; Su, kamdi	Transnational families and the family nexus: perspectives of Indonesian and Filipino children left behind by migrant parent(s)	2012	Environ. Plan. A	44	4	793-815	English	Primary	Exclude 6
Graham 2013	Graham, E.; Yeoh, B. S. A.	Child Health and Migrant Parents in South-East Asia: Risk and Resilience among Primary School-Aged Children INTRODUCTION	2013	Asian Pac. Migr. J.	22	3	297-314	English	Primary	Exclude 11
Guan	Guan, S. P.	Investigation and strategies on mental	2012	Education and Management			81-82	Chinese	Secondar	Exclude 3

2012		health development among rural left-behind children							y	
Hadi 1999	Hadi A	Overseas migration and the well-being of those left behind in rural communities of Bangladesh	1999	Asia Pacific Population Journal	14	1	https://www.ncbi.nlm.nih.gov/pubmed/12295290	English	Secondary	Exclude 6
Hamilton 2009	Hamilton, E. R.; Villarreal, A.; Hummer, R. A.	Mother's, Household, and Community US Migration Experience and Infant Mortality in Rural and Urban Mexico	2009	Popul. Res. Policy Rev.	28	2	123-142	English	Primary	Exclude 5
Hao 2013	HAO, G., ZHANG, R. and ZHANG, S.	Macronutrient and calcium intakes of rural left-behind children living in Ziyang	2013	Chinese Journal of School Health	34	6	644-650	Chinese	Primary	Exclude 6
He 2009	He, X.H., Zhang, J.Y., Mei, X.J.	Research on the mental health status of left-behind children and educational management strategy	2009	Agricultural Archaeology		3	81-83	Chinese	Primary	Exclude 5
He 2010	He, L. L. & Duan, C. Y.	Empirical study on the current situation and challenges of rural left-behind children	2010	Agricultural Archaeology	6		144-146	Chinese	Primary	Exclude 6
He 2010	He, L. L. & Qu, J. F.	Empirical study on the situation of rural left-behind children	2010	Chinese Agricultural Science Bulletin	19		91	Chinese	Primary	Exclude 6
Heymann 2009	Heymann J, Flores-Macias F, Hayes JA, Kennedy M, Lahaie C, Earle A	The impact of migration on the well-being of transnational families: new data from sending communities in Mexico	2009	Community, Work and Family	12	1	http://www.tandfonline.com/doi/full/10.1080/13668800802155704?scroll=top&needAccess=true	English	Secondary	Exclude 13 household level
Hiew 1992	Hiew, C. C.	Separated by their	1992	Environ. Behav.	24	2	206-225	English	Primary	Exclude 6
Hildebrandt 2005	Hildebrandt N, McKenzie DJ	The effects of migration on child health in Mexico	2005	Economia	Fall		https://s3.amazonaws.com/ssrc-cdn1/crmuploads	English	Secondary	Exclude 13 household

							/new_publication_3/%7BF394B238-ED51-DE11-AFAC-001CC477EC70%7D.pdf			level
Hoang 2012	Hoang, L. A.; Yeoh, B. S. A.	Sustaining Families across Transnational Spaces: Vietnamese Migrant Parents and their Left-Behind Children	2012	Asian Stud. Rev.	36	3	307-325	English	Primary	Exclude 3
Hoang 2015	Hoang, L. A.; Lam, T.; Yeoh, B. S. A.; Graham, E.	Transnational migration, changing care arrangements and left-behind children's responses in South-east Asia	2015	Child. Geogr.	13	3	263-277	English	Primary	Exclude 5
Howard 2017	Howard, L. L.; Stanley, D. L.	Remittances channels and the physical growth of Honduran children	2017	Int. Rev. Appl. Econ.	31	3	376-397	English	Primary	Exclude 5
Hu 2016	Hu, Y.; Lonne, B.; Burton, J.	The social exclusion of children left behind in China	2016	Asia Pac. J. Soc. Work Dev.	26	42796	77-87	English	Primary	Exclude 3
Hugo 2010	Hugo, G.; Ukwatta, S.	Sri Lankan Female Domestic Workers Overseas - The Impact on Their Children	2010	Asian Pac. Migr. J.	19	2	237-263	English	Primary	Exclude 5
Jampaklay 2013	Jampaklay, A.; Vapattanawong, P.	The Subjective Well-Being of Children in Transnational and Non-Migrant Households: Evidence from Thailand	2013	Asian Pac. Migr. J.	22	3	377-400	English	Primary	Exclude 6
Jayasuriya 2015	Jayasuriya, R.; Opeskin, B.	The Migration of Women Domestic Workers from Sri Lanka: Protecting the Rights of Children Left Behind	2015	Cornell Int. Law J.	48	3	579-638	English	Primary	Exclude 3
Ji 2016	Ji, Y.; Wang, Y. L.; Sun, L.; Zhang, Y.;	The Migrant Paradox in Children and the Role of Schools in Reducing Health Disparities: A Cross-Sectional Study of	2016	PLoS One	11	7	12	English	Primary	Exclude 4

	Chang, C.	Migrant and Native Children in Beijing, China								
Jia 2010	Jia, Z. B.; Shi, L. Z.; Cao, Y.; Delancey, J.; Tian, W. H.	Health-related quality of life of "left-behind children": a cross-sectional survey in rural China	2010	Qual. Life Res.	19	6	775-780	English	Primary	Exclude 6
Jia 2010	Jia, Z. B.; Tian, W. H.	Loneliness of left-behind children: a cross-sectional survey in a sample of rural China	2010	Child Care Health Dev.	36	6	812-817	English	Primary	Exclude 6
Jiang 2013	Jiang, F.P., Wang, X.Y.	Analysis on the current research on the mental health of left-behind children	2013	Chinese Rural Health Service Administration		2	171-174	Chinese	Secondary	Exclude 7
Kaciniene 2013	Kaciniene, I.; Pugevicius, A.	Family with Parents Abroad in the Context of Province	2013	Rural Development 2013: Proceedings, Vol6, Book 1	6		168-173	English	Primary	Exclude 2
Kanaiaupuni 1999	Kanaiaupuni SM, Donato KM	Migradollars and mortality: the effects of migration on infant survival in Mexico	1999	Demography	36	3	https://link.springer.com/content/pdf/10.2307%2F2648057.pdf	English	Secondary	Exclude 13 household level
Khan 2010	Khan, I. A.; Mahmood, S.; Yasin, G.; Shahbaz, B.	Impact of international migration on social protection of migrants families left behind in agrarian communities of district Toba Tek Singh, Punjab, Pakistan	2010	Pak. J. Agric. Sci.	47	4	425-428	English	Primary	Exclude 6
Kidman 2016	Kidman, R.; Palermo, T.	The relationship between parental presence and child sexual violence: Evidence from thirteen countries in sub-Saharan Africa	2016	Child Abuse Negl.	51		172-180	English	Primary	Exclude 5
Kong	Kong T, Meng X	The educational and health outcomes of the children of migrants	Unknown				http://globalnetwork.princeton.edu/piirs/Kong_Meng.pdf	English	Secondary	Exclude 6

Kroeger 2014	Kroeger, A.; Anderson, K. H.	Remittances and the human capital of children: New evidence from Kyrgyzstan during revolution and financial crisis, 2005-2009	2014	J. Comp. Econ.	42	3	770-785	English	Primary	Exclude 5
Lai 2011	Lai, C.F.	Investigation and thoughts on the mental health problems among left-behind children	2011	The Party Building and Ideological Education in Schools Sponsor		15	19-20	Chinese	Primary	Exclude 3
Lam 2013	Lam, T.; Ee, M.; Anh, H. L.; Yeoh, B. S. A.	Securing a Better Living Environment for Left-Behind Children: Implications and Challenges for Policies	2013	Asian Pac. Migr. J.	22	3	421-445	English	Primary	Exclude 7
Lan 2009	Lan, Y. L., Yan Li, X. J. Tang, Y. Y. Zhang, Y. N. Tan, and J. Y. Tan	Personality and depressive symptoms and their influential factors in children left-behind in rural area	2009	Chinese Journal of Public Health	25	8	901-903	Chinese	Primary	Exclude 11 Protocol for a study not meeting the inclusion criteria
Li 2009	Li, S. H.; Huang, H.; Cai, Y.; Xu, G.; Huang, F. R.; Shen, X. M.	Characteristics and determinants of sexual behavior among adolescents of migrant workers in Shangai (China)	2009	BMC Public Health	9		10	English	Primary	Exclude 4
Li 2009	Li, C.H.	Survey and analysis on the mental health status among rural left-behind children in Liaoning province	2009	Journal of Liaoning Educational Administration Institute	26	3	64-65	Chinese	Primary	Exclude 3
Li 2009	LI CY, SHEN LQ, HONG Y	STUDY ABOUT MENTAL HEALTH OF LEFT-BEHIND CHILDREN IN	2009	Modern Preventive	36	13	2508-2509	Chinese	Primary	Exclude 6

		RURAL AREA		Medicine						
Li 2012	Li, Y.J.; Wang X.J.	Investigation Research into Psychological Problems of Children of Migrant Workers	2012	Theory and Practice of Education	32	21	43－45	Chinese	Primary	Exclude 5
Li 2014	LI, Z. and SU, Q	Parental Non-agricultural Employment and Child Health: Evidence from Rural China.	2014	Population & Economics		3	51-58	Chinese	Primary	Exclude 6
Li 2015	Li, Q.; Liu, G.; Zang, W.	The health of left-behind children in rural China	2015	China Econ. Rev.	36		367-376	English	Primary	Exclude 6
Liang 2008	http://csda.albany.edu/imc/migration_and_the_well-being_of_children_in_china.pdf		2008					English	Secondary	Exclude 4
Lin 2010	Lin, X.H., Shen, M. and Wang, L.	Meta-analysis on the mental health of the left-in-hometown children in rural China.	2010	Acta Med Univ Sci Technol Huazhong		39	228-231	Chinese	Secondary	Exclude 7
Liu 2008	Liu, C.X.	A Case Study on the Female Children Staying in Rural Areas in Minority Region of Guizhou Province	2008	Guizhou Ethnic Studies	28	5	41-49	Chinese	Primary	Exclude 6
Liu 2009	Liu, Z. K.; Li, X. Y.; Ge, X. J.	Left Too Early: The Effects of Age at Separation From Parents on Chinese Rural Children's Symptoms of Anxiety and Depression	2009	Am. J. Public Health	99	11	2049-2054	English	Primary	Exclude 5
Liu 2011	Liu, B. Q.	Commentary on the research on the mental health status of left-behind children	2011	Continuing Education Research		12	51-54	Chinese	Secondary	Exclude 3
Liu 2012	Liu, C.Y., Zhong, Z.H., Pan, J.P., Wang, Y.X., Zhong, Y., Yang, X., Hu, C. and Cai, L.L.	The current situation of children neglect and its influencing factors for rural children aged 0—6 years in Chongqing	2012	Chinese journal of preventive medicine	46	1	33-37	Chinese	Secondary	Exclude 3
Liu	LIU, X.,	Comparison of mental health status	2012	Chinese General	15	13	1507-1510	Chinese	Primary	Exclude 6

2012	WANG, X. and YANG, Y.	between left-behind children under different guardianships and non-left-behind children.		Practice						
Liu 2016	Liu, S. Z.; Johnson, J. A.; Destech Publicat, Inc	Population Mobility and Children's Education	2016	2016 2nd International Conference on Social Science and Development (Icssd 2016)			165-170	English	Primary	Exclude 7
Lu 2009	Lu, D.P.	Key issues and current situation of preschool rural left-behind children	2009	Journal of China Youth College for Political Sciences		3	33-38	Chinese	Primary	Exclude 6
Lu 2010	Lu, Y.	Rural-urban migration and health: Evidence from longitudinal data in Indonesia	2010	Soc. Sci. Med.	70	3	412-419	English	Primary	Exclude 4
Lu 2014	Lu, W.J.; Hu, Y.; Zhang, D.K.; Peng, G.D.; Yang, S.P.; Xu, Y.H.; Xie, S; Liu, W.W.; Han, J.	Left-behind characteristics and the effects of related factors on psychological health of rural left-behind children	2014	Maternal and Child Health Care of China	29	21	3463-3466	Chinese	Primary	Exclude 5
Lu 2014	Lu, Y., Yu, Y.J., Zhu,Y.	Mental health status and family characteristics among rural left-behind children in Guizhou	2014	Chinese Journal of School Health	35	9	1413-1415	Chinese	Primary	Exclude 6
Lu 2016	Lu, S.; Lin, Y. T.; Vikse, J. H.; Huang, C. C.	Well-being of migrant and left-behind children in China: Education, health, parenting, and personal values	2016	Int. J. Soc. Welf.	25	1	58-68	English	Primary	Exclude 5
Luo 2008	Luo, J. Y.; Peng, X. C.; Zong, R.; Yao, K. B.; Hu, R. S.; Du, Q. Y.;	The status of care and nutrition of 774 left-behind children in rural areas in China	2008	Public Health Rep.	123	3	382-389	English	Primary	Exclude 13 less than 3 months

	Fang, J. Q.; Zhu, M. Y.									
Luo 2011	Luo, R. F.; Zhang, L. X.; Liu, C. F.; Zhao, Q. R.; Shi, Y. J.; Miller, G.; Yu, E.; Sharbono, B.; Medina, A.; Rozelle, S.; Martorell, R.	Anaemia among Students of Rural China's Elementary Schools: Prevalence and Correlates in Ningxia and Qinghai's Poor Counties	2011	J. Heatlh Popul. Nutr.	29	5	471-485	English	Primary	Exclude 5 (no direct/clear comparison between LBC and non-LBC; defined on basis of father vs mother absence only)
Luo 2014	Luo, J.; Xiang, B.; Ding, Y.P.; Peng, J.; Zhu, C.C.	Analysis on the influencing factors of injuries among the left behind Children in rural area of Hanchuan City	2014	Maternal and Child Health Care of China		4	553-555	Chinese	Primary	Exclude 6
Macours 2010	Macours, K.; Vakis, R.	Seasonal Migration and Early Childhood Development	2010	World Dev.	38	6	857-869	English	Primary	Exclude 13 less than 3 months
Mansuri 2006	https://openknowledge.worldbank.org/bitstream/handle/10986/8423/wps3946.pdf?sequence=1&isAllowed=y		2006					English	Secondary	Exclude 13 household level
Marteleto 2016	Marteleto, L. J.; Cavanagh, S.; Prickett, K.; Clark, S.	Instability in Parent-Child Coresidence and Adolescent Development in Urban South Africa	2016	Stud. Fam. Plan.	47	1	19-38	English	Primary	Exclude 6
Mazzu	Mazzucato, V.	Transnational families and the well-	2015	Soc. Sci. Med.	132		208-214	English	Primary	Exclude 7

cato 2015		being of children and caregivers who stay in origin countries Introduction								
Meng 2010	Meng, X.; Zhang, T.J.	Research on the emotional characteristics and mental health problems among rural left-behind children	2010	Theory Research		3	68-70	Chinese	Primary	Exclude 6
Micu-Serbu 2014	Micu-Serbu, I. B.; Gafencu, M.; Nyiredi, A.; Bajireanu, D.; Stehlic, R.; Stan, V. O.	Risk and resilience: children's perspectives through drawings on parent's economical migration and ethnicity	2014	Second World Congress on Resilience: from Person to Society			627-633	English	Primary	Exclude 10
Mu 2010	Mu, M., Wang, S.F., Wang, Y.M., Ma, X.H., Yu, T., Wang, Y., Zhang, W.M.	Analysis on nutrition and health status of left-behind children in rural Anhui	2010	Acta Universitatis Medicinalis Anhui	45	6	829-831	Chinese	Primary	Exclude 6
Murphy 2016	Murphy, R.; Zhou, M. H.; Tao, R.	Parents' Migration and Children's Subjective Well-being and Health: Evidence from Rural China	2016	Popul. Space Place.	22	8	766-780	English	Primary	Exclude 6
Nanthamongkolchai 2011	http://journals.sagepub.com/doi/pdf/10.1177/1010539511424535		2011					English	Secondary	Exclude 5
Nguyen 2015	Nguyen, C. V.; Nguyen, H. Q.	Do internal and international remittances matter to health, education and labor of children and adolescents? The case of Vietnam	2015	Child. Youth Serv. Rev.	58		28-34	English	Primary	Exclude 5
Ning 2013	Ning, M. X.; Chang, H. H.	Migration decisions of parents and the nutrition intakes of children left at home in rural China	2013	Agric. Econ.	59	10	467-477	English	Primary	Exclude 6
Nyanja	Nyanjaya, A.	The plight of absent fathers caused by	2012	HTS Teol. Stud.-	68	1	10	English	Primary	Exclude 3

ya 2012	K.; Masango, M. J.	migrant work: Its traumatic impact on adolescent male children in Zimbabwe		Theol. Stud.						
Obregon-Velasco 2015	Obregon-Velasco, N.; Rivera-Heredia, M. E.	Impact of the father's migration in youth: when migration becomes abandonment	2015	CienciaUat	10	1	56-67	English	Primary	Exclude 3
Olayiwola 2006	Olayiwola, I. O.; Bandipo, M. S.	Nutrition and childcare practices -- the situation in Nigeria	2006	West African Journal of Nursing	17	1	21-27	English	Primary	Exclude 8
Onyango 2011	Onyango, E. B.; Khasakhala, A.; Agwanda, A. T.; Kimani, M.; K'Oyugi, B.	Effect of mothers' migration on under-two mortality in Kenya	2011	African Population Studies	25	2	543-555	English	Primary	Exclude 4
Owusu	http://www.childhoodstoday.org/download.php?id=74							English	Secondary	Exclude 3
Pajaron 2016	Pajaron, M.	Heterogeneity in the Intrahousehold Allocation of International Remittances: Evidence from Philippine Households	2016	J. Dev. Stud.	52	6	854-875	English	Primary	Exclude 6
Palos-Lucio 2015	Palos-Lucio, G.; Flores, M.; Rivera-Pasquel, M.; Salgado-de-Snyder, V. N.; Monterrubio, E.; Henao, S.; Macias, N.	Association between migration and physical activity of school-age children left behind in rural Mexico	2015	International journal of public health	60	1	49-58	English	Primary	Exclude 13 household level
Parasuraman	Parasuraman, S.	Migration and its effect on the family	1986	Special Issue: The family	47	1	41640	English	Primary	Exclude 8

1986										
Peng 2008	Peng, X. C.; Luo, J. Y.; Yao, K. B.; Hu, R. S.; Du, Q. Y.; Zhu, M. Y.	The status on care and nutrition of 774 children staying in rural areas while parents were in towns	2008	Zhonghua liu xing bing xue za zhi = Zhonghua liuxingbingxue zazhi	29	9	860-864	English	Primary	Exclude 13 English abstract of Chinese language paper; already included in Chinese search
Peng 2008	Peng, X.C., Luo, J.Y., Yao, K.B., Hu, R.S., Du, Q.Y. and Zhu, M.Y.,	The status on care and nutrition of 774 children staying in rural areas while parents were in towns.	2008	Chinese Journal of Epidemiology	29	9	860-864	Chinese	Primary	Exclude 6
Peng 2013	PENG, X.C. and LUO, J.Y.	Status and influencing factors of growth and development among rural left-behind children in Hunan province	2013	Chinese Journal of Public Health,	29	1	34-37	Chinese	Primary	Exclude 11 Protocol for a study not meeting the inclusion criteria
Peng 2014	Peng, J.; Shi, Y.J.; Gao, C.F.	Preschool left-behind children's health status and intervention strategies	2014	Education Review	10	10	78-80	Chinese	Primary	Exclude 6
Persona 2004	Persona, L.; Shimo, A. K.;	Profile of adolescents with repeated pregnancies attended at a prenatal	2004	Revista latino-americana de	12	5	745-750	English	Primary	Exclude 5

	Tarallo, M. C.	clinic		enfermagem						
Piao 2006	Piao, T.J.	Let all children grow up healthy	2006	Ethnic Education of China		9	20-22	Chinese	Primary	Exclude 3
Piko 2007	https://www.ncbi.nlm.nih.gov/pubmed/17130141							English	Secondary	Exclude 4
Pilarova 2016	Pilarova, T.; Kandakov, A.; Sgem	The impact of parental migration on student's involvement in household	2016	Sgem 2016, Bk 2: Political Sciences, Law, Finance, Economics and Tourism Conference Proceedings, Vol V			785-791	English	Primary	Exclude 6
Ponce 2011	Ponce, J.; Olivie, I.; Onofa, M.	The role of international remittances in health outcomes in Ecuador: Prevention and response to shocks	2011	International Migration Review	45	3	727-745	English	Primary	Exclude 4
Pribilsky 2001	Pribilsky, J.	Nervios and 'modern childhood' - Migration and shifting contexts of child life in the Ecuadorian Andes	2001	Child.-Glob. J. Child Res.	8	2	251-273	English	Primary	Exclude 3
Qin 2010	Qin, J.; Albin, B.	The mental health of children left behind in rural China by migrating parents: a literature review	2010	Journal of Public Mental Health	9	3	42461	English	Primary	Exclude 7
Racaite 2016	Racaite, J.; Jakubauskiene, M.; Surkiene, G.	Children left alone as a public health concern: a case of Lithuania	2016	Eur. J. Public Health	26		1	English	Primary	Exclude 10
Raslaviciene 2002	Raslaviciene, G.; Zaborskis, A.	The development of mixed emotional and behavioral disorders in children raised in foster care institutions	2002	Medicina (Kaunas, Lithuania)	38	7	759-768	English	Primary	Exclude 4
Reichert 2015	Reichert, Andrea; Atkins, Lydia; Ituah, Livio; Atkins, Richard	Family structure and sexual health behavior	2015	Caribbean adolescents: Some public health concerns.			59-71	English	Primary	Exclude 4
Riosmena	Riosmena, F.; Frank, R.;	U.S. Migration, Translocality, and the Acceleration of the Nutrition	2012	Ann. Assoc. Am. Geogr.	102	5	1209-1218	English	Primary	Exclude 4

2012	Akresh, I. R.; Kroeger, R. A.	Transition in Mexico								
Rivera-Heredi a 2013	Rivera-Heredia, M. E.; Martinez-Servin, L. G.; Obregon-Velasco, N.	Factors associated with depressive symptomatology in adolescents. The role of family migration and individual, family and social resources	2013	Salud Ment.	36	2	115-122	English	Primary	Exclude 4
Robila 2011	Robila, M.	Parental Migration and Children's Outcomes in Romania	2011	J. Child Fam. Stud.	20	3	326-333	English	Primary	Exclude 5
Robila 2014	Robila, M.	The Impact of Migration on Children's Psychological and Academic Functioning in the Republic of Moldova	2014	Int. Migr.	52	3	221-235	English	Primary	Exclude 6
Rousse au 2008	Rousseau, C.; Hassan, G.; Measham, T.; Lashley, M.	Prevalence and correlates of conduct disorder and problem behavior in Caribbean and Filipino immigrant adolescents	2008	European Child & Adolescent Psychiatry	17	5	264-273	English	Primary	Exclude 4
Rus 2012	Rus, O. A.; Pascanu, R. P.; Coman, L. V.; Cozma, R.; Andreica, B.	The impact of parents emigration on children anxiety	2012	European Psychiatry	27		no pagination	English	Primary	Exclude 10
Saber 2016	Saber, Al-Sobaihi; Keiko, Nakamura; Masashi, Kizuki	Undernutrition among children under 5 years of age in Yemen: Role of adequatechildcare provided by adults under conditions of food insecurity	2016	Journal of Rural Medicine	11	2	47-57	English	Primary	Exclude 4
Santos Mendo za 1959	Santos Mendoza, E.	[Problems of health in the abandoned child]	1959	Revista de sanidad y asistencia social	24		385-96	English	Primary	Exclude 4

Save the Children	Save the Children	Left behind, left out: impact on children and families of mothers migrating for work abroad	2006	Save the Children			https://resourcecentre.savethechildren.net/node/8007/pdf/left_behind_left_out_-_the_impact_of_children_and_families_of_mothers_migrating_for_work_abroad.pdf	English	Secondary	Exclude 13
Sawyer 2017	Sawyer, C. B.; Marquez, J.	Senseless Violence Against Central American Unaccompanied Minors: Historical Background and Call for Help	2017	J. Psychol.	151	1	69-75	English	Primary	Exclude 4
Schapiro 2013	Schapiro, N. A.; Kools, S. M.; Weiss, S. J.; Brindis, C. D.	Separation and Reunification: The Experiences of Adolescents Living in Transnational Families	2013	Curr. Probl. Pediatr. Adolesc. Health Care	43	3	48-68	English	Primary	Exclude 7
Schmeer 2008	Schmeer, Kammi	Changing childhood household contexts and individual well-being in Mexico and the Philippines	2008	Dissertation Abstracts International Section A: Humanities and Social Sciences	68	7-A	3167	English	Primary	Exclude 8
Senaratne 2011	Senaratne, B. C. V.; Perera, H.; Fonseka, P.	Mental health status and risk factors for mental health problems in left-behind children of women migrant workers in Sri Lanka	2011	The Ceylon medical journal	56	4	153-8	English	Primary	Exclude 6
Shang 2003	Shang, X. Y.; Wu, X. M.	Protecting children under financial constraints: 'Foster Mother Villages' in Datong	2003	J. Soc. Policy	32		549-570	English	Primary	Exclude 3

Shen 2008	Shen, M., Yang, S.B., Guo, Y., Liu, X.X. and Du, Y.K.	Study on nonfatal injuries among home-stranded children in rural environment of Hubei province.	2008	Chinese Journal of Epidemiology	29	4	333-337	Chinese	Primary	Exclude 6
Shenk 2013	Shenk, M. K.; Starkweather, K.; Kress, H. C.; Alam, N.	Does Absence Matter? A Comparison of Three Types of Father Absence in Rural Bangladesh	2013	Hum. Nat.-Interdiscip. Biosoc. Perspect.	24	1	76-110	English	Primary	Exclude 1
Sheppard 2014	Sheppard, P.; Snopkowski, K.; Sear, R.	Father absence and reproduction-related outcomes in Malaysia, a transitional fertility population	2014	Human nature (Hawthorne, N.Y.)	25	2	213-234	English	Primary	Exclude 6
Sheriff 2011	Sheriff, A.; Rahim, A.; Lailabi, M. P.; Gopi, J.	Unintentional injuries among children admitted in a tertiary care hospital in North Kerala	2011	Indian journal of public health	55	2	125-127	English	Primary	Exclude 4
Shi 2012	Shi, S.S.; Cui, W.X.	Current research on the health of left-behind children	2012	Chinese journal of preventive medicine	33	6	757-759	Chinese	Primary	Exclude 3
Silver 2014	Silver, A.	Families Across Borders: The Emotional Impacts of Migration on Origin Families*	2014	Int. Migr.	52	3	194-220	English	Primary	Exclude 6
Siqueira 1982	Siqueira, L.	Family disintegration in Latin America: the consequences for children	1982	Draper Fund report		11	42836	English	Primary	Exclude 6
Smeekens 2012	Smeekens, C.; Stroebe, M. S.; Abakoumkin, G.	The impact of migratory separation from parents on the health of adolescents in the Philippines	2012	Social Science and Medicine	75	12	2250-2257	English	Primary	Exclude 6
Smith 2004	Smith, A.; Lalonde, R. N.; Johnson, S.	Serial Migration and Its Implications for the Parent-Child Relationship: A Retrospective Analysis of the Experiences of the Children of	2004	Cultural Diversity and Ethnic Minority Psychology	10	2	107-122	English	Primary	Exclude 6

		Caribbean Immigrants								
Smith-Greenaway 2014	Smith-Greenaway, E.; Thomas, K. J. A.	Exploring Child Mortality Risks Associated with Diverse Patterns of Maternal Migration in Haiti	2014	Popul. Res. Policy Rev.	33	6	873-895	English	Primary	Exclude 5
Smuk 2016	Smuk, O.	Comprehensive support and correction of children-orphans' development	2016	Sci. Educ.		9	161-168	English	Primary	Exclude 6
Soares Luz 2015	Soares Luz, Luciana	Investments in children's health and schooling in rural southern Mozambique: The role of mothers' decision-making autonomy and father's labor migration	2015	Dissertation Abstracts International Section A: Humanities and Social Sciences	75	7-A(E)	No-Specified	English	Primary	Exclude 5
Sobkin 2015	Sobkin, Vladimir S.; Skobeltsina, Ksenia N.	Shared activities of parents and their preschool children during family pastime	2015	Special Issue: 4th Annual international research-to-practice conference "Early Childhood Care and Education"	8	2	52-60	English	Primary	Exclude 6
Song 2009	Song, Y. and Zhang, Y.	Parental migration, child health and healthcare services utilization in rural China	2009	Population Research	33	6	57-66	Chinese	Primary	Exclude 6
Srinivasan 1988	Srinivasan, T. N.; Raman, K. J.	Early child parent separation and risk for childhood psychopathology	1988	Indian journal of psychiatry	30	3	283-9	English	Primary	Exclude 4
Ssengonzi 2002	Ssengonzi, R.; De Jong, G. F.; Stokes, C. S.	The effect of female migration on infant and child survival in Uganda	2002	Popul. Res. Policy Rev.	21	5	403-431	English	Primary	Exclude 5
Steinhausen	Steinhausen, H. C.	Psychiatric disorders in children and family dysfunction. A study of migrant	1985	Social psychiatry. Sozialpsychiatrie.	20	1	42897	English	Primary	Exclude 4

1985		workers' families		Psychiatrie sociale						
Stoklosa 1981	Stoklosa, Bogumila	Specific conditions for the child growing up in the incomplete family	1981	Specyficzne warunki wzrastania dziecka w rodzinie niepelnej	24	4	496-504	English	Primary	Exclude 6
Su 2012	Su, P.; Hu, C.; Li, L.; Zhang, Y.; Pang, P.; Cheng, S.; Zheng, H.	[Study on dietary patterns and its effect on infant health among left-behind children aged 1-4 years old with both parents working out in rural Anhui]	2012	Wei sheng yan jiu = Journal of hygiene research	41	5	754-759	English	Primary	Exclude 13 English abstract of Chinese language paper; already included in Chinese search
Su 2012	Su, P., Hu, C., Li, L., Zhang, Y., Pang, P., Cheng, S. and Zheng, H.	Study on dietary patterns and its effect on infant health among left-behind children aged 1-4 years old with both parents working out in rural Anhui	2012	Journal of hygiene research	41	5	754-759	Chinese	Primary	Exclude 11 Protocol for a study not meeting the inclusion criteria
Su 2013	Su, S.; Li, X.; Lin, D.; Xu, X.; Zhu, M.	Psychological adjustment among left-behind children in rural China: the role of parental migration and parent-child communication	2013	Child: Care, Health & Development	39	2	162-170	English	Primary	Exclude 6
Suarez-	Suarez-	I Felt Like My Heart Was Staying	2011	J. Adolesc. Res.	26	2	222-257	English	Primary	Exclude 3

Orozco 2011	Orozco, C.; Bang, H. J.; Kim, H. Y.	Behind: Psychological Implications of Family Separations & Reunifications for Immigrant Youth								
Sun 2010	Sun, B.; Ge, H.M.; Li, Z.D.; Qian, Z.; Yin, C.; Dong, Y.; Ren, X.F.	Food intake and nutrition among rural left-behind children aged 0 to 5	2010	Maternal and Child Health Care of China	25	9	1237-1240.	Chinese	Primary	Exclude 5
Sun 2017	Sun, M.; Xue, Z. M.; Zhang, W.; Guo, R.; Hu, A. M.; Li, Y. H.; Mwansisya, T. E.; Zhou, L.; Liu, C.; Chen, X. D.; Huang, X. J.; Tao, H. J.; Shi, J. C.; Liu, Z. N.; Rosenheck, R.	Psychotic-like experiences, trauma and related risk factors among "left-behind" children in China	2017	Schizophr. Res.	181		43-48	English	Primary	Exclude 6
Sunil 2012	Sunil, T. S.; Flores, M.; Garcia, G. E.	New evidence on the effects of international migration on the risk of low birthweight in Mexico	2012	Maternal and Child Nutrition	8	2	185-198	English	Primary	Exclude 13 household level
Sushma 2014	Sushma, B.; Padmaja, G.; Agarwal, Swati	Internalizing problems, externalizing problems and depression among children under institutional care	2014	Journal of Psychosocial Research	9	1	45-54	English	Primary	Exclude 4
Tang 2016	Tang, X. Y.; Geater, A.; McNeil, E.; Zhou, H. X.;	Parental migration and children's timely measles vaccination in rural China: a cross-sectional study	2016	Trop. Med. Int. Health	21	7	886-894	English	Primary	Exclude 6

	Deng, Q. Y.; Dong, A. H.; Li, Q.									
Tang 2017	Tang, Z.	What Makes a Difference to Children's Health in Rural China? Parental Migration, Remittances, and Social Support	2017	Chin. Sociol. Rev.	49	2	89-109	English	Primary	Exclude 13 non-valid scale
Tao 2014	Tao, X. W.; Guan, H. Y.; Zhao, Y. R.; Fan, Z. Y.	Mental health among left-behind preschool-aged children: Preliminary survey of its status and associated risk factors in rural China	2014	Journal of International Medical Research	42	1	120-129	English	Primary	Exclude 6
Tavares 2004	Tavares, Beatriz Franck; Beria, Jorge Umberto; Lima, Mauricio Silva de	Factors associated with drug use among adolescent students in southern Brazil	2004	Fatores associados ao uso de drogas entre adolescentes escolares.	38	6	787-96	English	Primary	Exclude 4
Teller 1983	Teller, C. H.; Butz, W. P.	Circular migration, development conditions and young child malnutrition in Guatemala [tables]	1983				13 p.	English	Primary	Exclude 10
Terrelonge 2014	Terrelonge, S. C.	For Health, Strength, and Daily Food: The Dual Impact of Remittances and Public Health Expenditure on Household Health Spending and Child Health Outcomes	2014	J. Dev. Stud.	50	10	1397-1410	English	Primary	Exclude 5
Tollman 1999	Tollman, S. M.; Herbst, K.; Garenne, M.; Gear, J. S. S.; Kahn, K.	The Agincourt demographic and health study - Site description, baseline findings and implications	1999	South African Medical Journal	89	8	858-864	English	Primary	Exclude 6
Tong	Tong, Y. Y.;	The Association Between Parental	2015	Eur. J. Popul.	31	5	561-586	English	Primary	Exclude 6

2015	Luo, W. X.; Piotrowski, M.	Migration and Childhood Illness in Rural China								
UNICEF 2009	UNICEF	The impact of international migration on children left behind	2009	UNICEF			http://www.childrenleftbehind.eu/wp-content/uploads/2011/05/2009_UNICEF_ChildLeftB_impact.pdf	English	Secondary	Exclude 6
Unknown	Unknown	Parental migration and health of children left behind	Unknown				https://iussp.org/sites/default/files/event_call_for_papers/Parental%20migration%20and%20health%20of%20children%20left%20behind.pdf	English	Secondary	Exclude 6
Valtolina 2012	Valtolina GG, Colombo C	Psychological well-being, family relations, and developmental issues of children left behind	2012	Psychological reports: Relationships and Communications	111	3	905-928	English	Secondary	Exclude 7
Wang 2008	Wang, T.; Gao, W. B.; Liu, Z. K.	The impact of parents' migration from rural to urban areas on the mental health of their children left behind in China	2008	Int. J. Psychol.	43	42828	767-767	English	Primary	Exclude 13 English abstract of Chinese language paper; already included in Chinese

										search
Wang 2009	Wang, J.X.	Sexual safety among left-behind adolescents	2009	Contemporary Youth Research		3	17-22	Chinese	Primary	Exclude 5
Wang 2010	Wang, F.; Sun, Y.; Niu, J.; Gong, L.; Cai, B.; Sun, L.	[SCL-90 test result on the left-behind children in rural area: a meta-analysis]	2010	Wei sheng yan jiu = Journal of hygiene research	39	2	224-227	English	Primary	Exclude 7
Wang 2010	Wang, F., Sun, Y., Niu, J., Gong, L., Cai, B. and Sun, L.	SCL-90 test result on the left-behind children in rural area: a meta-analysis	2010	Journal of hygiene research	39	2	224-227	Chinese	Secondar y	Exclude 7 Review study
Wang 2011	Wang, Y. J.; He, B. Y.; Fang, L. H.; Li, H. J.	Preliminary study on the health status among the "left-behind" children in the Xian-tao rural area of Hubei Province	2011	Chinese Journal of Contemporary Pediatrics	13	12	977-980	English	Primary	Exclude 13 English abstract of Chinese language paper; already included in Chinese search
Wang 2012	Wang, R. X.; Zhu, L. Q.; Niu, S. X.	Investigation on mental health of left-behind primary and secondary school students in northern remote rural of Hebei Province	2012	Journal of Jilin University Medicine Edition	38	6	1214-1218	English	Primary	Exclude 13 English abstract of Chinese language paper; already included

										in Chinese search
Wang 2012	Wang, F.M.	Investigation and analysis on the mental health status of rural Year 3 to Year 6 students	2012	Modern Educational Science		3	28-29	Chinese	Primary	Exclude 6
Wang 2015	Wang, L. M.; Mesman, J.	Child Development in the Face of Rural-to-Urban Migration in China: A Meta-Analytic Review	2015	Perspect. Psychol. Sci.	10	6	813-831	English	Primary	Exclude 7
Wang 2015	Wang, X.; Ling, L.; Su, H.; Cheng, J.; Jin, L.; Sun, Y. H.	Self-concept of left-behind children in China: a systematic review of the literature	2015	Child: Care, Health & Development	41	3	346-355	English	Primary	Exclude 7
Wang 2015	Wang, L. F.; Feng, Z. Z.; Yang, G. Y.; Yang, Y. L.; Dai, Q.; Hu, C. B.; Liu, K. Y.; Guang, Y.; Zhang, R.; Xia, F.; Zhao, M. X.	The epidemiological characteristics of depressive symptoms in the left-behind children and adolescents of Chongqing in China	2015	J. Affect. Disord.	177		36-41	English	Primary	Exclude 5
Wang 2015	Wang, J. L.; Zhang, D. J.; Zimmerman, M. A.	Resilience theory and its implications for Chinese adolescents	2015	Psychological reports	117	2	354-375	English	Primary	Exclude 3
Wang 2015	Wang, Fei; Pan, Jianping; Zhang, Songjie; Zhang, Hua;	Impact factors analysis on child neglect of children aged 3-6 year-old in rural areas of China	2015	Zhonghua Yu Fang Yi Xue Za Zhi	49	10	866-72	English	Primary	Exclude 13 English abstract of Chinese

	Wang, Weiqing; Tao, Fangbiao; Qin, Rui; Gu, Guixiong; Shi, Shuhua; Zhang, Jing; Du, Qiyun; Zhong, Zhaohui; Jiao, Feng; Wang, Huishan; Xu, Tao; Wang, Guixiang; Xi, Weiping; Pang, Songtao; Wang, Xin; Cao									language paper; already included in Chinese search
Wang 2015	Wang, Y.; Hesketh, T.; Zhou, X. D.	Behavioural and emotional problems in children affected by parental migration in rural China: a cross-sectional survey	2015	Lancet	386		82-82	English	Primary	Exclude 10
Wang 2016	Wang, L.; Wei, Y.; Ma, Y.; Wang, T.	Fundamental literature and hot topics on rural left-behind children in China: a bibliometric analysis	2016	Child: Care, Health & Development	42	6	852-858	English	Primary	Exclude 7
Wei 2011	Wei, S.; Ju, L.; Li, M.; Wang, W.	Child health and nutrition: Getting better and facing new challenges in China	2011	Australasian Medical Journal	4	3	123-132	English	Primary	Exclude 3
Wen 2015	Wen, M.; Su, S. B.; Li, X. M.; Lin, D. H.	Positive youth development in rural China: The role of parental migration	2015	Soc. Sci. Med.	132		261-269	English	Primary	Exclude 6
Wen 2015	Wen, Yi; Liu, Qin; Zhang,	Mental resilience tested with the Resilience Scale for Chinese	2015	Chinese Mental Health Journal	29	11	826-832	English	Primary	Exclude 13

	Fan; Huang, Ke; Lu, Jia; Guo, Xue; Wang, Hong	Adolescents (RSCA) in Chinese children: A meta-analysis								English abstract of Chinese language paper; already included in Chinese search
Wen 2016	Wen, M.; Li, K. L.	Parental and sibling migration and high blood pressure among rural children in China	2016	J. Biosoc. Sci.	48	1	129-142	English	Primary	Exclude 6
Wickramage 2015	Wickramage, K, Siriwardhana C, Peiris S	Promoting the health of left-behind children of Asian labour migrants: evidence for policy and action	2015	IOM Report		44		English	Secondary	Exclude 7
Wittig 1997	Wittig, M. C. W.; Wright, J. D.; Kaminsky, D. C.	Substance use among street children in Honduras	1997	Substance Use and Misuse	32	42954	805-827	English	Primary	Exclude 6
Wray 1976	Wray, S. R.; McLaren, E.	Parent-child separation as a determinant of psychopathology in children: a Jamaican study	1976	The West Indian medical journal	25	4	251-7	English	Primary	Exclude 8
Wu 2008	Wu, X.Y.; Gao, C.X.	A Survey of the Psychological State of Left-behind Children in the Rural Areasof Qiandongnan Prefecture	2008	Journal of Guizhou University for Nationalities		5	111-115	Chinese	Primary	Exclude 6
Wu 2013	Wu, Chenggu; Xie Jun; Luo Xingjian et al.	Logistic Regression Analysis on Infection Rate of Enterobius Vermicularis of Preschool Children in Chongqing City	2013	Chinese Journal of Health Statistics		3	167-172	Chinese	Primary	Exclude 6
Wu 2014	Wu, R.; Li, J.; Liu, Q.;	Influences of life event and coping style on left-behind middle school	2014	Wei sheng yan jiu = Journal of	43	4	586-590	English	Primary	Exclude 13

	Wang, H.	student mental health in a three-gorge area county		hygiene research						English abstract of Chinese language paper; already included in Chinese search
Wu 2015	Wu, Yi-Le; Zhao, Xue; Ding, Xiu-Xiu; Yang, Hui-Yun; Qian, Zhen-Zhong; Feng, Fang; Lu, Shan-Shan; Hu, Cai-Yun; Gong, Feng-Feng; Sun, Ye-Huan	A prospective study of psychological resilience and depression among left-behind children in China	2015	Journal of health psychology				English	Primary	Exclude 5
Wu 2017	Wu, J.; Zhang, J. S.	The Effect of Parental Absence on Child Development in Rural China	2017	Asian Econ. Policy. Rev.	12	1	117-134	English	Primary	Exclude 5
Xing 2016	Xing, S. F.; Zhou, Q.; Archer, M.; Yue, J. H.; Wang, Z. Y.	Infant temperamental reactivity, maternal and grandparental sensitivity: Differential susceptibility for behavior problems in China	2016	Early Hum. Dev.	101		99-105	English	Primary	Exclude 4
Xing 2016	Xing, Q. Y.; Zhong, X. N.; Yang, G. J.	The influence of "left-behind phenomenon" on quality of life of children in Chongqing	2016	Academic Journal of Second Military Medical	37	4	424-428	English	Primary	Exclude 13 English

				University						abstract of Chinese language paper; already included in Chinese search
Xing 2017	Xing, H. Y.; Yu, W.; Xu, F. J.; Chen, S. M.	Influence of social support and rearing behavior on psychosocial health in left-behind children	2017	Health Qual. Life Outcomes	15		6	English	Primary	Exclude 6
Xiong 2013	Xiong, Y.; Cheng, X.	Analysis of psychological resilience of left-behind high school students in Nanchong rural	2013	Wei sheng yan jiu = Journal of hygiene research	42	6	955-959	English	Primary	Exclude 13 English abstract of Chinese language paper; already included in Chinese search
Xu 2013	Xu, Y. and Ma, L.	Self-harm behavioral characteristics and rationales among rural left-behind adolescents	2013	Journal of South-Central University for Nationalities	33	4	90-96	Chinese	Primary	Exclude 5
Xu 2015	Xu, H.; Mu, L.; Xie, X.	Relationship of left-behind children's mental health, coping style, personality and self-efficacy	2015	Wei sheng yan jiu = Journal of hygiene research	44	4	559-569	English	Primary	Exclude 13 English abstract of Chinese language

										paper; already included in Chinese search
Yabiku 2012	Yabiku, S. T.; Agadjanian, V.; Cau, B.	Labor migration and child mortality in Mozambique	2012	Social Science and Medicine	75	12	2530-2538	English	Primary	Exclude 13 less than 3 months
Yan & Lu 2013	Yan, M.H. & Lu, C.M.	Health Investigation and Analysis on the Left-behind and Non Left-behind Children in Lianyungang Rural Regions	2013	Journal of Anhui Agricultural Sciences	41	2	898-900	Chinese	Primary	Exclude 6
Yang 2008	Yang, C.L.	Survey and analysis on the left-behind primary-school and middle-school students	2008	Education research monthly		12	36-37	Chinese	Primary	Exclude 3
Yang 2009	Yang, L.J.	The socialization of left-behind children in rural Wenzhou	2009	Education Review		6	97-100	Chinese	Primary	Exclude 3
Yang 2014	Yang, L., Chen, W.X.	Comparison between different patterns of parental absence and health status of left-behind young children	2014	Maternal and Child Health Care of China	29	22	3652-3655	Chinese	Primary	Exclude 6
Yang 2014	Yang, L., Chen, W.X., Yang, K.C.	The health status of left-behind children in Nanchong City	2014	Maternal and Child Health Care of China	29	28	4613-4615	Chinese	Primary	Exclude 6
Yang 2016	YANG Xue-wen， ZHA Wen-ting, ZHANG Guo-chao， LIANG Ｒong, LIANG Wei-jun	Multilevel model study on status and influencing factors of accident injury among rural school-age children in Hunan Province	2016	Chinese Journal of Disease Control and Prevention	20	3	266-270	Chinese	Secondary	Exclude 7

Yao 2010	Yao, Y. S.; Kang, Y. W.; Jin, Y. L.; Gong, W. Z.; Chen, Y.; Zheng, L.; An, Z.	A prevalence survey on the mental health of left behind adolescent in Anhui province	2010	Zhonghua liu xing bing xue za zhi = Zhonghua liuxingbingxue zazhi	31	12	1359-1362	English	Primary	Exclude 13 English abstract of Chinese language paper; already included in Chinese search
Yao 2012	Yao, Y. S.; Kang, Y. W.; Jin, Y. L.; Chen, Y.; Gong, W. Z.; Zheng, L.; An, Z.; Tao, F. B.; Hao, J. H.	Analysis on physical and mental health and related influential factors among those 'left behind' adolescents in Anhui province	2012	Zhonghua liu xing bing xue za zhi = Zhonghua liuxingbingxue zazhi	33	7	681-684	English	Primary	Exclude 13 English abstract of Chinese language paper; already included in Chinese search
Yao 2012	Yao, Y.S., Kang, Y.W., Jin, Y.L., Chen, Y., Gong, W.Z., Zheng, L., An, Z., Tao, F.B. and Hao, J.H.	Analysis on physical and mental health and related influential factors among those 'left behind' adolescents in Anhui province	2012	Chinese Journal of Epidemiology	33	7	681-684	Chinese	Primary	Exclude 6
Ye	YE, M.,	The Analysis of the Influence Factors	2006	Medicine &	27	6	67-69	Chinese	Primary	Exclude 3

2006	ZHANG, J. P., & HE, D. R	of the Psychological Health of Guarded Children and Ways to Solve it		Philosophy (Humanistic & Social Medicine Edition)						
Ye 2006	YE Jingzhong , WANG Yihuan , ZHANG Keyun and LU Jixia	Impacts of the Migrating Parents on Life of the Left-behind Children	2006	Issues in Agricultural Economy		4	19-24	Chinese	Primary	Exclude 6
Ye 2010	Ye, M.; Zhang, J.P.	Influential factors on mental health of rural junior middle school students	2010	Chinese Journal of Behavioral Medicine and Brain Science	19	6	547-549	Chinese	Primary	Exclude 6
Ye 2011	Ye, J. Z.; Pan, L.	Differentiated childhoods: impacts of rural labor migration on left-behind children in China	2011	J. Peasant Stud.	38	2	355-377	English	Primary	Exclude 6
Ye 2011	Ye, J. Z.	Left-behind children: the social price of China's economic boom	2011	J. Peasant Stud.	38	3	613-620	English	Primary	Exclude 3
Yeung 2016	Yeung, Wei-Jun Jean; Park, Hyunjoon	Growing up in one-parent families in Asia	2016	Marriage & Family Review	52	4276 7	41640	English	Primary	Exclude 7
Yeung 2016	Yeung, Wei-Jun Jean; Gu, Xiaorong	Left behind by parents in China: Internal migration and adolescents' well-being	2016	Marriage & Family Review	52	4276 7	127-161	English	Primary	Exclude 6
Yu 2011	Yu, D.; Liu, A.; Yu, W.; Zhang, B.; Zhang, J.; Jia, F.; Li, J.; Zhao, L.	Status of malnutrition and its influencing factors in children under 5 years of age in poor areas of China in 2009	2011	Wei sheng yan jiu = Journal of hygiene research	40	6	714-718	English	Primary	Exclude 13 English abstract of Chinese language paper;

										already included in Chinese search
Yu 2012	Yu, H.; Lu, K. R.	Mental health and education status of rural left-behind primary-school students	2010	Chinese Journal of School Health	26	19	426-432	Chinese	Primary	Exclude 6
Yu 2013	Yu, D.; Liu, A.; Yu, W.; Jia, F.; Li, J.; Zhao, L.	Malnutrition status and influencing factors in left-behind children with migrant worker mother in poor areas in China	2013	Annals of Nutrition and Metabolism	63		1044	English	Primary	Exclude 8
Yun 2015	Yun, Huang; Xiao-Ni, Zhong; Qing-Ying, Li; Dan, Xu; Xuan-Lin, Zhang; Chao, Feng; Guo-Xiu, Yang; Yun-Yun, Bo; Bing, Deng	Health-related quality of life of the rural-China left-behind children or adolescents and influential factors: a cross-sectional study	2015	Health & Quality of Life Outcomes	13	1	43009	English	Primary	Exclude 6
Yu-tao 2013	Yu-tao, Feng; Chang-zhou, H. U.; Qiu-li, L. I.; Miao-miao, L. I. U.; Xue, Y. U.; Xiu-ying, D. A. I.	Detectable rate and correlative factors of behavior problems among Hui nationality left-behind children in rural district of Ningxia	2013	Chinese Journal of Behavioral Medical and Brain Science		2	157-159	English	Primary	Exclude 13 English abstract of Chinese language paper; already included in Chinese

										search
Zeng 2009	Zeng, Rong; Zhang, Ling-Li; Luo, Jia-You; Gong, Wen-Jie; Du, Qi-Yun; Wu, Hong	[Study on emotional, behavioral problems and related determinants among stranded children aged 4 to 7 years in the rural areas of China]	2009	Zhonghua liu xing bing xue za zhi = Zhonghua liuxingbingxue zazhi	30	7	706-9	English	Primary	Exclude 13 English abstract of Chinese language paper; already included in Chinese search
Zhan 2014	Zhan, X. F.; Li, S. P.; Liu, C. F.; Zhang, L. X.	Effect of Migration on Children's Self-esteem in Rural China	2014	China World Econ.	22	4	83-101	English	Primary	Exclude 6
Zhang 2006	Zhang, D.S.	Mental health status among rural left-behind children and the corresponding strategies	2006	The Modern Education Journal		3	19-22	Chinese	Primary	Exclude 5
Zhang 2008	ZHANG, J.Y. and HE, X.H.	Study on relationship between mental health and personality of parents-absent children	2008	Chinese Journal of Public Health	24	8	932-933	Chinese	Primary	Exclude 3
Zhang 2011	Zhang, F.; Liu, Q.; Zhao, Y.; Sun, M. H.; Wang, H.	Left-behind children's mental health problems: A systematic review	2011	Chinese Journal of Evidence-Based Medicine	11	8	849-857	English	Primary	Exclude 7
Zhang 2012	Zhang, X. J.; Zhang, Y.; Yang, H.; Zhang, Y. J.; Jiang, L. Q.; Liu, S. L.; Bi,	Neurodevelopment of left-behind children under 3 years old in a town in China	2012	Hong Kong Journal of Paediatrics	17	4	217-222	English	Primary	Exclude 6

	Y.; Liu, Y. X.; Chen, J.; Wei, X. P.; Li, T. Y.									
Zhang 2013	Zhang, F.; Liu, Q.; Guo, X.; Wang, H.; He, J.; Wang, Y.	Resilience status and impact factors in rural middle school students in Three Gorges areas	2013	Wei sheng yan jiu = Journal of hygiene research	42	4	632-636	English	Primary	Exclude 13 English abstract of Chinese language paper; already included in Chinese search
Zhang 2013	Zhang F., Liu,Q., Guo, X., Wang, H., He, J.Y., Wang Y.	Mental health and its relationship with resilience in children in rural three gorges area	2013	Journal of Chongqing Medical University	38	8	822-826	Chinese	Primary	Exclude 6
Zhang 2015	Zhang, N.; Becares, L.; Chandola, T.	Does the timing of parental migration matter for child growth? A life course study on left-behind children in rural China	2015	BMC Public Health	15		12	English	Primary	Exclude 6
Zhang 2015	Zhang, N.; Becares, L.; Chandola, T.; Callery, P.	Intergenerational differences in beliefs about healthy eating among carers of left-behind children in rural China: A qualitative study	2015	Appetite	95		484-491	English	Primary	Exclude 3
Zhang 2015	Zhang, F.; Zhang, T.; Xiong, J.; Zhang, L.;	Analysis on prevalence and influence factors of smoking and drinking among middle shool students of Tujia and Miao in Xiangxi Area	2015	Wei sheng yan jiu = Journal of hygiene research	44	5	750-766	English	Primary	Exclude 13 English abstract of

	Chen, J.									Chinese language paper; already included in Chinese search
Zhang 2016	Zhang, N.; Becares, L.; Chandola, T.	A multilevel analysis of the relationship between parental migration and left-behind children's macronutrient intakes in rural China	2016	Public Health Nutr.	19	11	1913-1927	English	Primary	Exclude 6
Zhao 2008	Zhao, K. F.; Su, H.; Fang, X. H.; He, L.; Chen, J.; Chen, M. C.; Ye, D. Q.	Study on the distribution and risk factors of injuries among home-stranded children in rural area of Anhui province	2008	Zhonghua liu xing bing xue za zhi = Zhonghua liuxingbingxue zazhi	29	4	338-342	English	Primary	Exclude 13 English abstract of Chinese language paper; already included in Chinese search
Zhao 2008	Zhao, J.X., Liu, X. and Shen, J.L.	Left-home adolescents’ perception of social support networks and their associations with individual depression and loneliness: Variable-centered and person-centered perspectives.	2008	Psychological Development and Education	23	1	36-42	Chinese	Primary	Exclude 6
Zhao 2010	Zhao, F.	Mental health status of and education strategies for left-behind children	2010	Journal of Capital Normal University		3	128-130	Chinese	Primary	Exclude 6
Zhao 2013	Zhao, Jingxin; Liu, Xia;	Peer rejection, peer acceptance and psychological adjustment of left-behind	2013	Acta Psychologica	45	7	797-810	English	Primary	Exclude 13

	Zhang, Wenxin	children: The roles of parental cohesion and children's cultural beliefs about adversity		Sinica						English abstract of Chinese language paper; already included in Chinese search
Zhao 2015	Zhao, J. X.; Liu, X.; Wang, M. F.	Parent-child cohesion, friend companionship and left-behind children's emotional adaptation in rural China	2015	Child Abuse Negl.	48		190-199	English	Primary	Exclude 5
Zhao 2016	Zhao, F. Q.; Yu, G. L.	Parental Migration and Rural Left-Behind Children's Mental Health in China: A Meta-Analysis Based on Mental Health Test	2016	J. Child Fam. Stud.	25	12	3462-3472	English	Primary	Exclude 7
Zhao 2016	Zhao, Y. J.; Du, M. M.; Gao, X.; Xiao, Y.; Shah, C.; Sun, H. Q.; Chen, F. Q.; Yang, L. L.; Yan, Z. H.; Fu, Y. C.; Lui, S.	Altered brain network topology in left-behind children: A resting-state functional magnetic resonance imaging study	2016	Child Abuse Negl.	62		89-99	English	Primary	Exclude 6
Zhao 2016	Zhao, Chenyue; Wang, Feng; Li, Leah; Zhou,	Persistent effects of parental migration on psychosocial wellbeing of left-behind children in two Chinese provinces: a cross-sectional survey	2016	Lancet (London, England)	388 Suppl 1		S6	English	Primary	Exclude 10

	Xudong; Hesketh, Therese									
Zheng 2015	Zheng, Y.; Zheng, X.	Current state and recent developments of child psychiatry in China	2015	Child and Adolescent Psychiatry and Mental Health			no pagination	English	Primary	Exclude 7
Zheng 2016	Zheng, X. X.	The Case Studies of Depression among Migrant Children and Left-Behind Children during China's Rapid Urbanization	2016	Proceedings of the 2016 International Conference on Public Management	9		392-395	English	Primary	Exclude 3
Zhong 2012	Zhong, Y.; Zhong, Z. H.; Pan, J. P.; Wang, Y. X.; Liu, C. Y.; Yang, X.; Hu, C.; Cai, L. L.; Xu, Y.	[The situation of children neglect between left-behind children and living-with-parents children in rural areas of two western provinces of China]	2012	Zhonghua yu fang yi xue za zhi [Chinese journal of preventive medicine]	46	1	38-41	English	Primary	Exclude 13 English abstract of Chinese language paper; already included in Chinese search
Zhong 2015	Zhong, Yin; Zhong, Zhaohui; Pan, Jianping; Li, Qunying; Zhong, Yun; Sun, Haoling	Research on child neglect situation and influential factors of left-behind children and living-with-parents children aged 6-17 year-old in rural areas of two provinces, western China	2015	Zhonghua Yu Fang Yi Xue Za Zhi	49	10	873-8	English	Primary	Exclude 13 English abstract of Chinese language paper; already included in

										Chinese search
Zhou 2005	Zhou, Z.K., Sun, X., Liu, Y. and Zhou, D.M.	Psychological development and education problems of children left in rural areas	2005	Journal of Beijing Normal University		1	71-79	Chinese	Primary	Exclude 6
Zhou 2011	Zhou, W. J.; Gao, W. B.; Sun, X. Y.; Luo, J.	[Psychological resilience features of urban migrant children and rural left-behind children in Sichuan province of China]	2011	Beijing da xue xue bao. Yi xue ban = Journal of Peking University. Health sciences	43	3	386-390	English	Primary	Exclude 13 English abstract of Chinese language paper; already included in Chinese search
Zhou 2012	Zhou, Z.H.	Investigation on the current stuation of mental health status among rural left-behind children in Jangxi Province and the corresponding strategies	2012	Literary Circles of CPC History		3	54-55	Chinese	Primary	Exclude 5
Zhou 2013	Zhou, Y., Lv, C. and Xu, F.	Chinese Journal of Special Education	2013	Chinese Journal of Special Education		10	52-58	Chinese	Primary	Exclude 6
Zhou 2015	Zhou, C. L.	Analysis about the path to improve the growth environment of rural children left behind	2015	Proceedings of the 2015 International Conference on Social Science and Technology Education	18		560-562	English	Primary	Exclude 13 English abstract of Chinese language paper; already included

										in Chinese search
Zhunio 2012	Zhunio, M. C.; Vishwasrao, S.; Chiang, E. P.	The influence of remittances on education and health outcomes: a cross country study	2012	Appl. Econ.	44	35	4605-4616	English	Primary	Exclude 6

Funnel plots

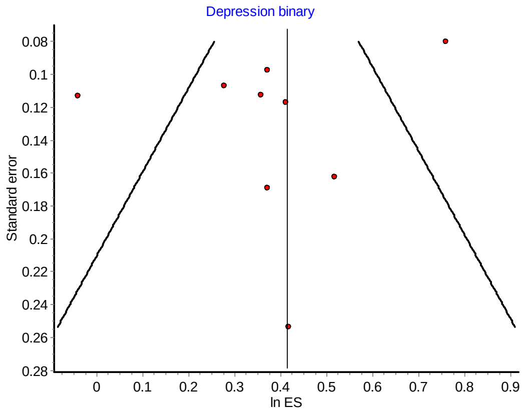

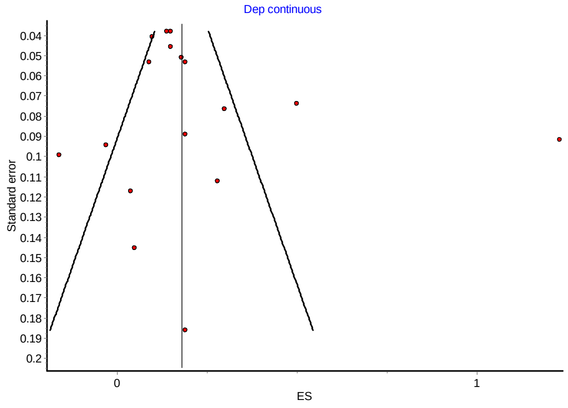

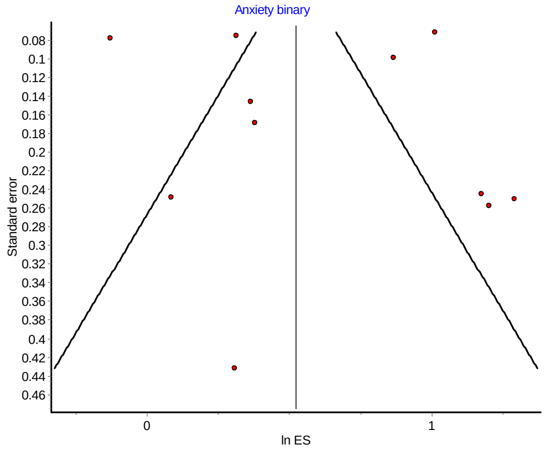

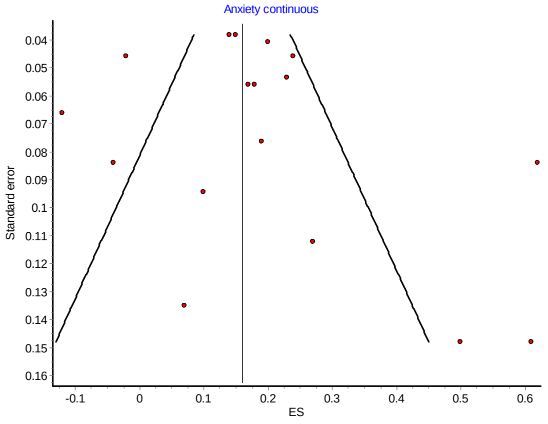

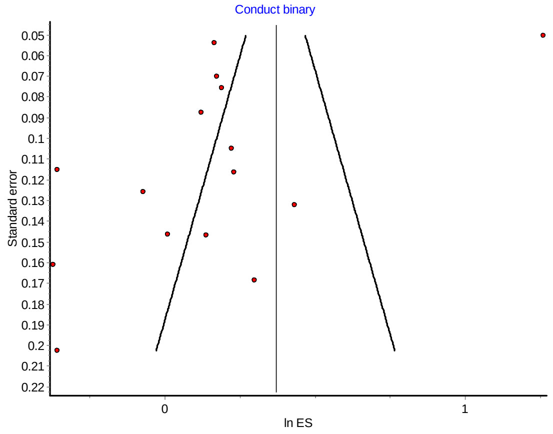

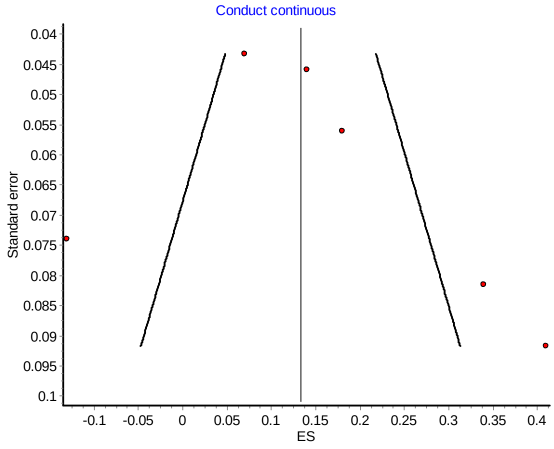

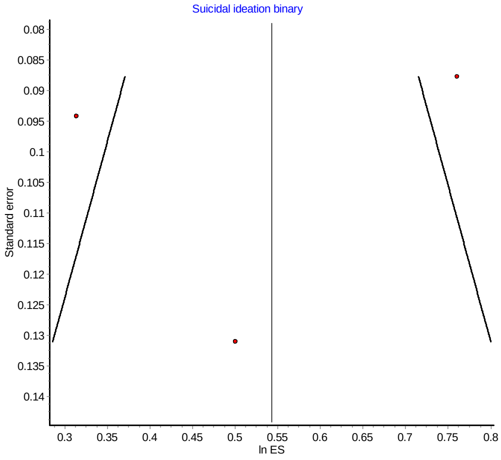
Suicidal ideation binary
Standard error
ln ES

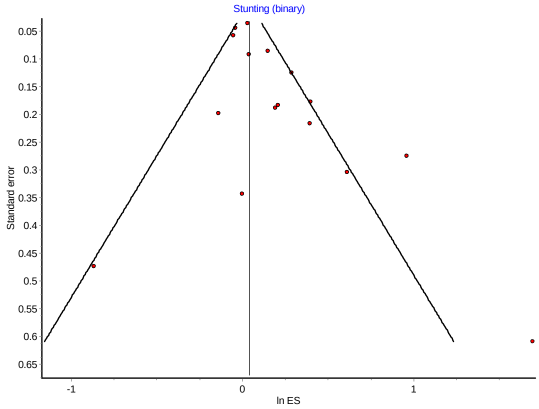
Stunting (binary)
Standard error
ln ES

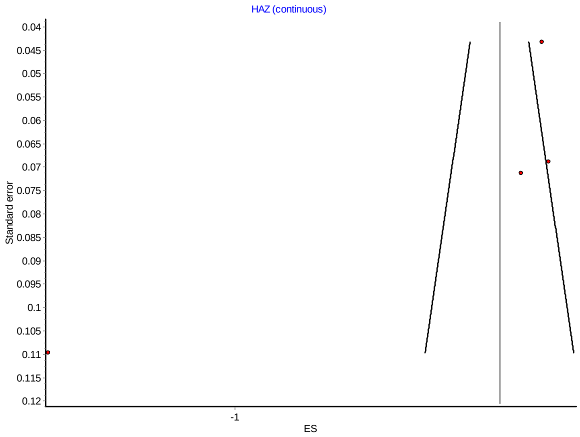
HAZ (continuous)
Standard error
ES

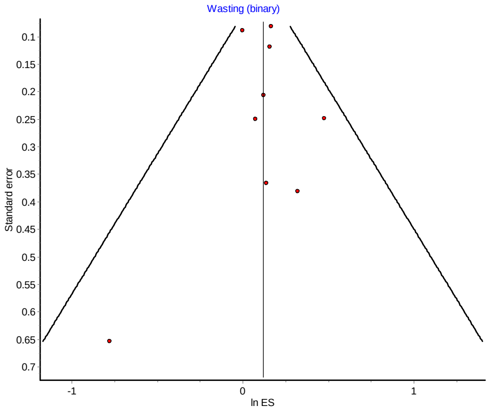
Wasting (binary)
Standard error
ln ES

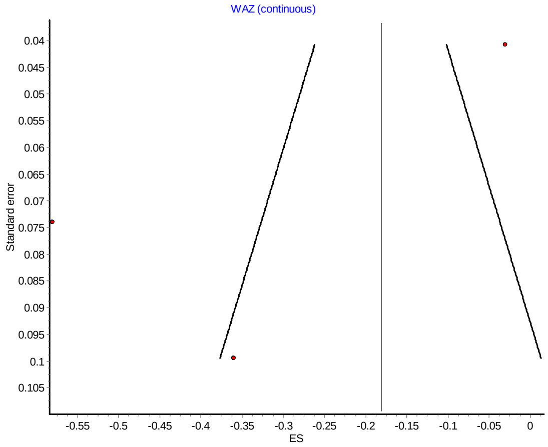
WAZ (continuous)
Standard error
ES

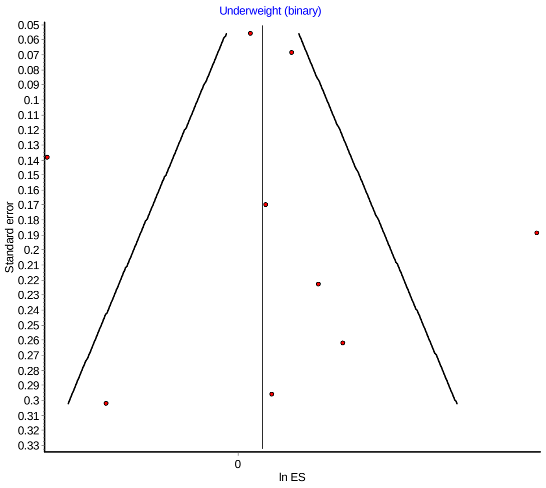
Underweight (binary)
Standard error
0.05
0.06
0.07
0.08
0.09
0.1
0.11
0.12
0.13
0.14
0.15
0.16
0.17
0.18
0.19
0.2
0.21
0.22
0.23
0.24
0.25
0.26
0.27
0.28
0.29
0.3
0.31
0.32
0.33
0
ln ES

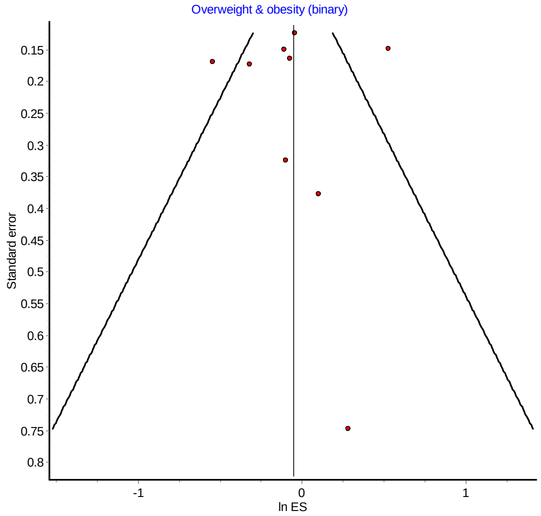
Overweight & obesity (binary)
Standard error
0.15
0.2
0.25
0.3
0.35
0.4
0.45
0.5
0.55
0.6
0.65
0.7
0.75
0.8
-1
0
1
ln ES

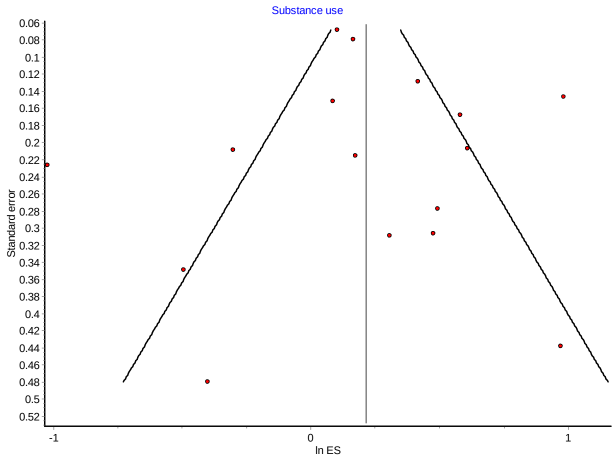
Substance use
Standard error
0.06
0.08
0.1
0.12
0.14
0.16
0.18
0.2
0.22
0.24
0.26
0.28
0.3
0.32
0.34
0.36
0.38
0.4
0.42
0.44
0.46
0.48
0.5
0.52
-1
0
1
ln ES

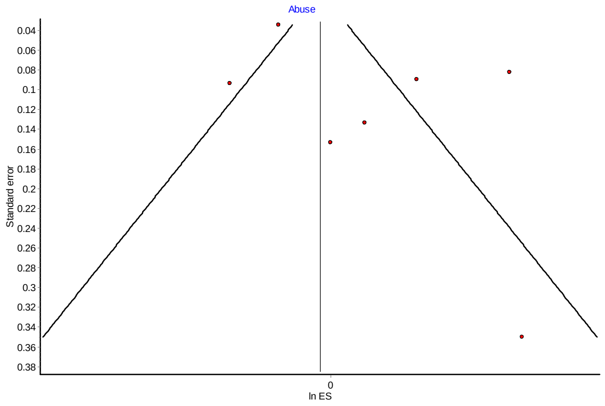
Abuse
Standard error
0.04
0.06
0.08
0.1
0.12
0.14
0.16
0.18
0.2
0.22
0.24
0.26
0.28
0.3
0.32
0.34
0.36
0.38
0
ln ES

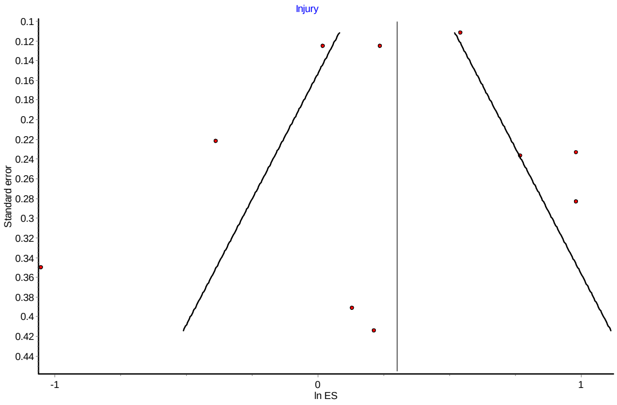
Injury
Standard error
0.1
0.12
0.14
0.16
0.18
0.2
0.22
0.24
0.26
0.28
0.3
0.32
0.34
0.36
0.38
0.4
0.42
0.44
-1
0
1
ln ES

Subgroup analysis

Both parents absent – mental health (binary outcomes)

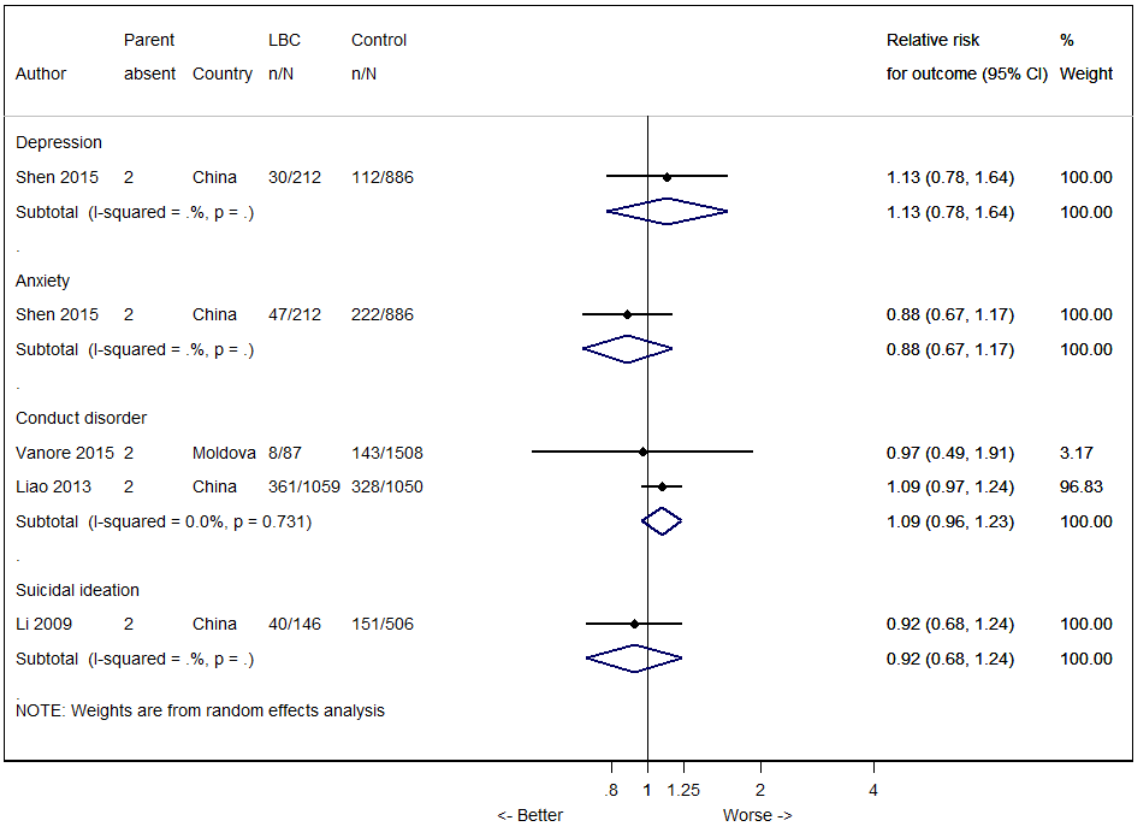

Both parents absent – mental health (continuous outcomes)

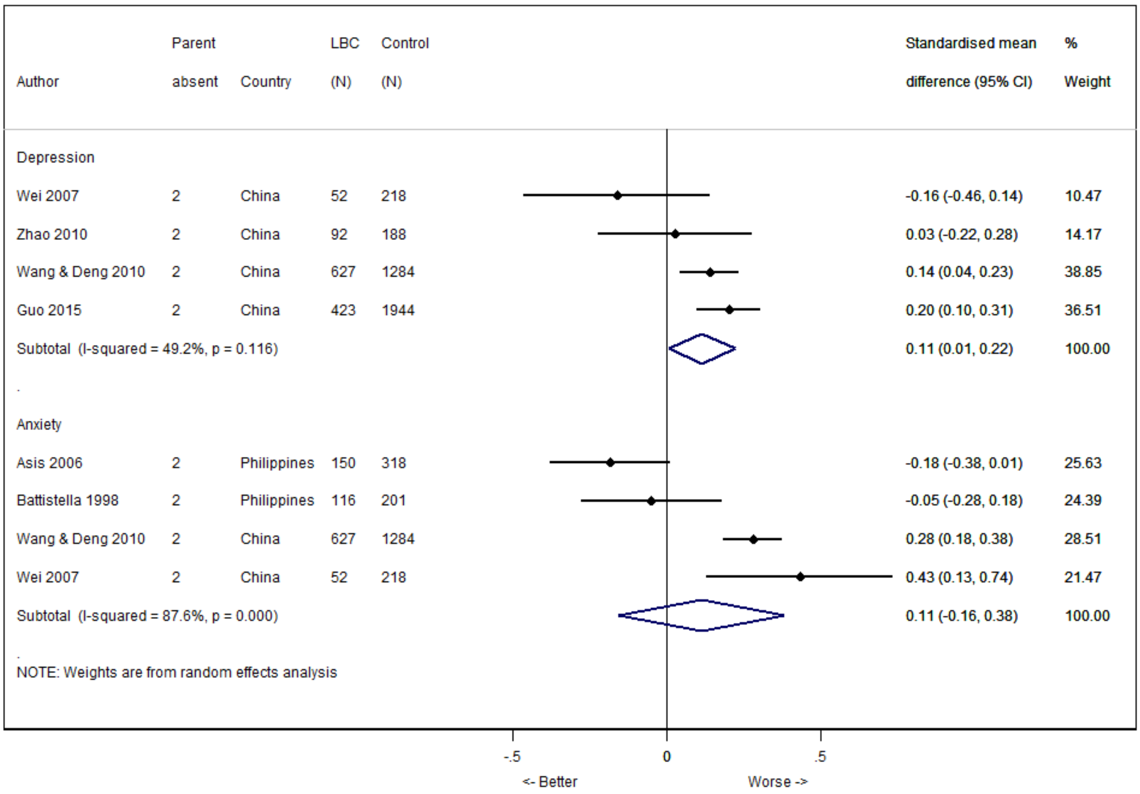

Both parents absent – nutrition (binary outcomes)

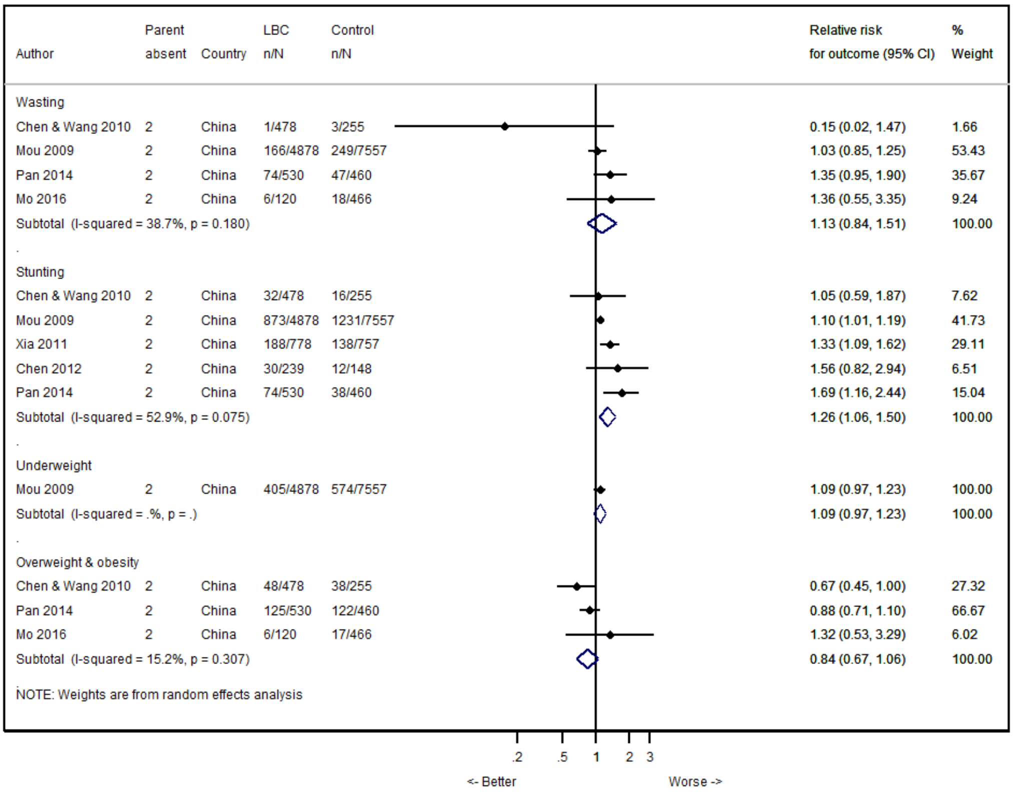

Both parents absent – nutrition (continuous outcomes)

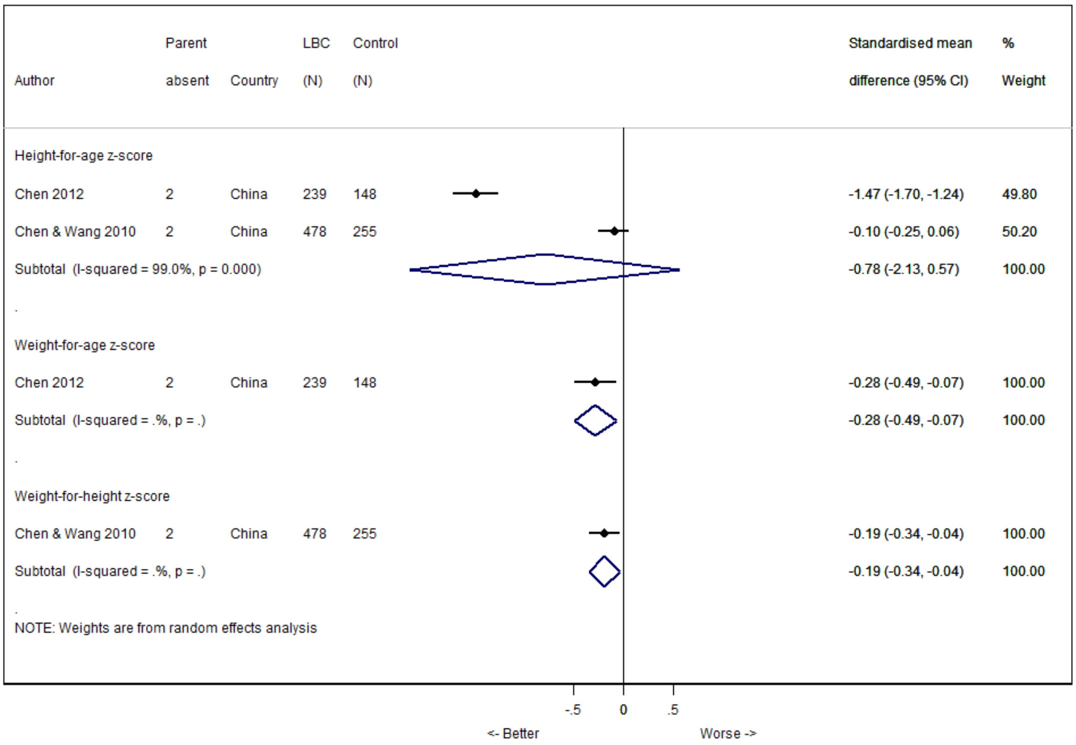

Both parents absent – other outcomes (binary outcomes)

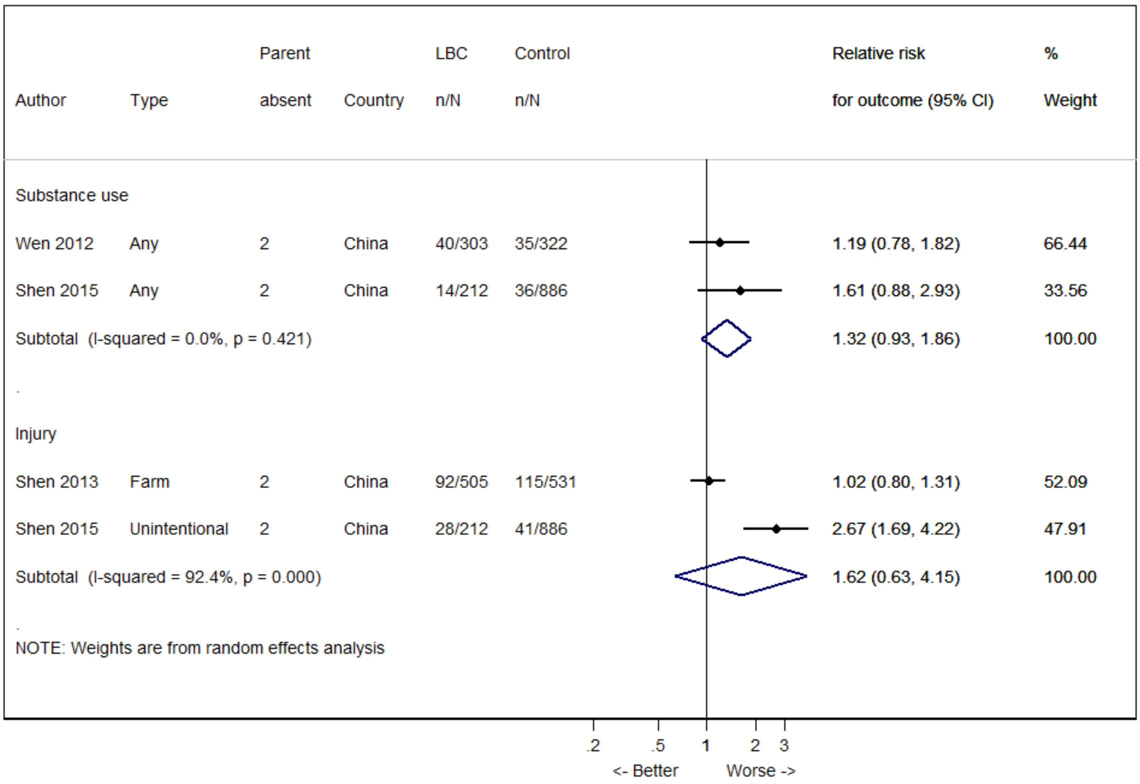

One parent absent – mental health (binary outcomes)

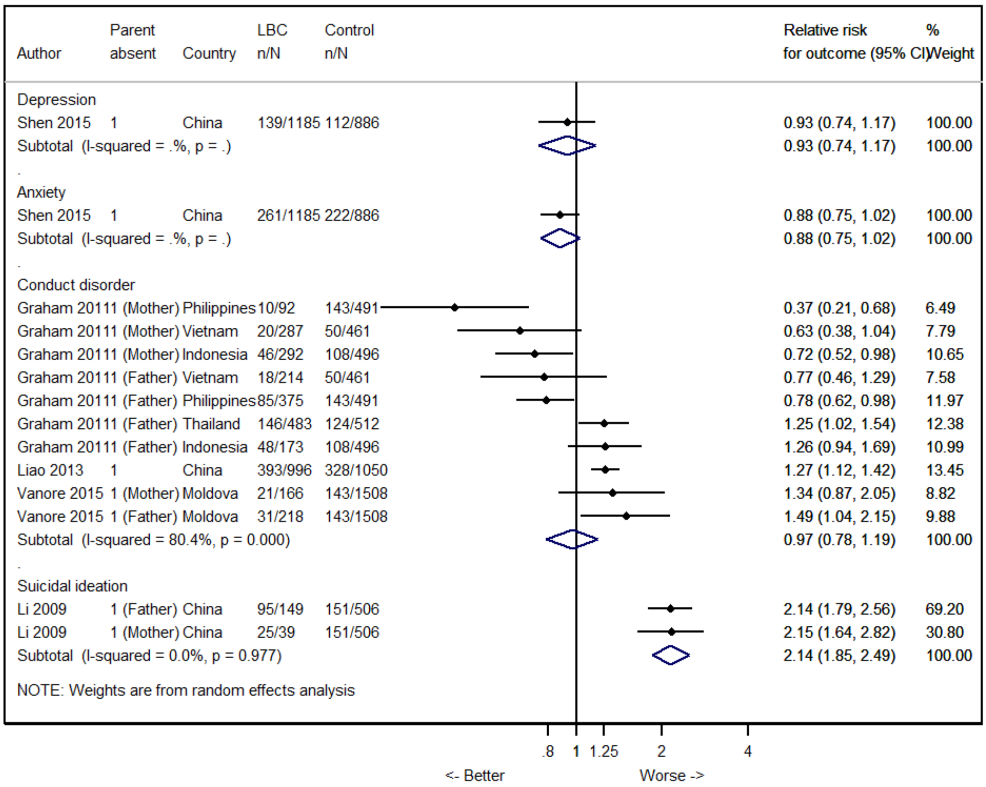

One parent absent – mental health (continuous outcomes)

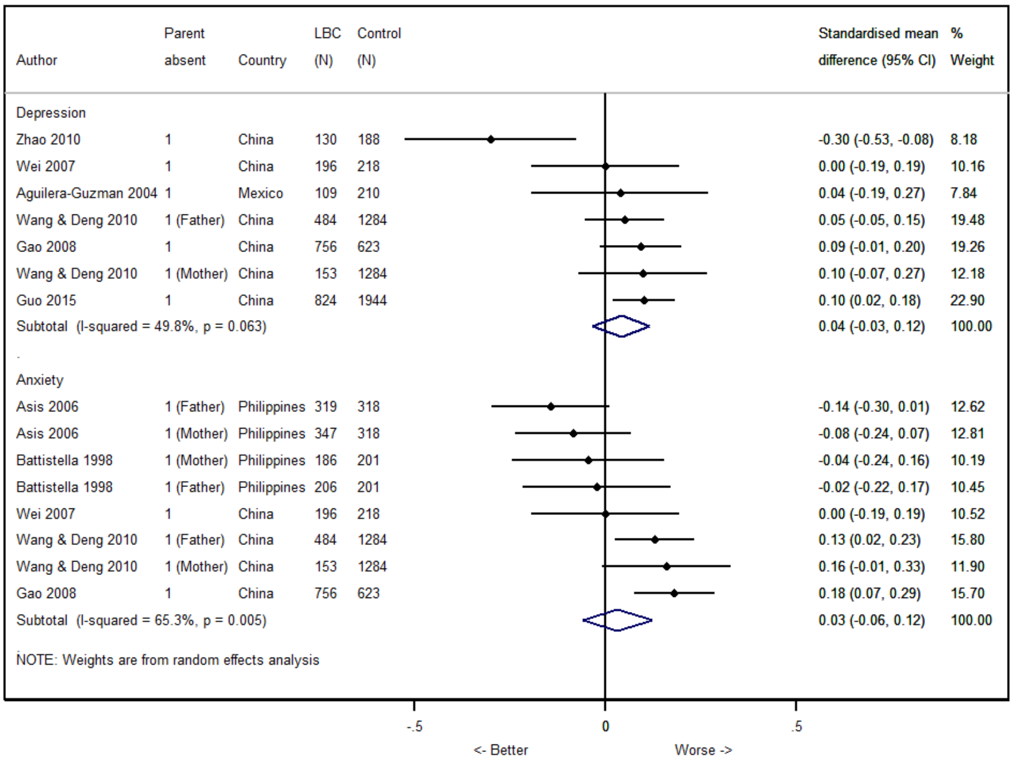

One parent absent – nutrition (binary outcomes)

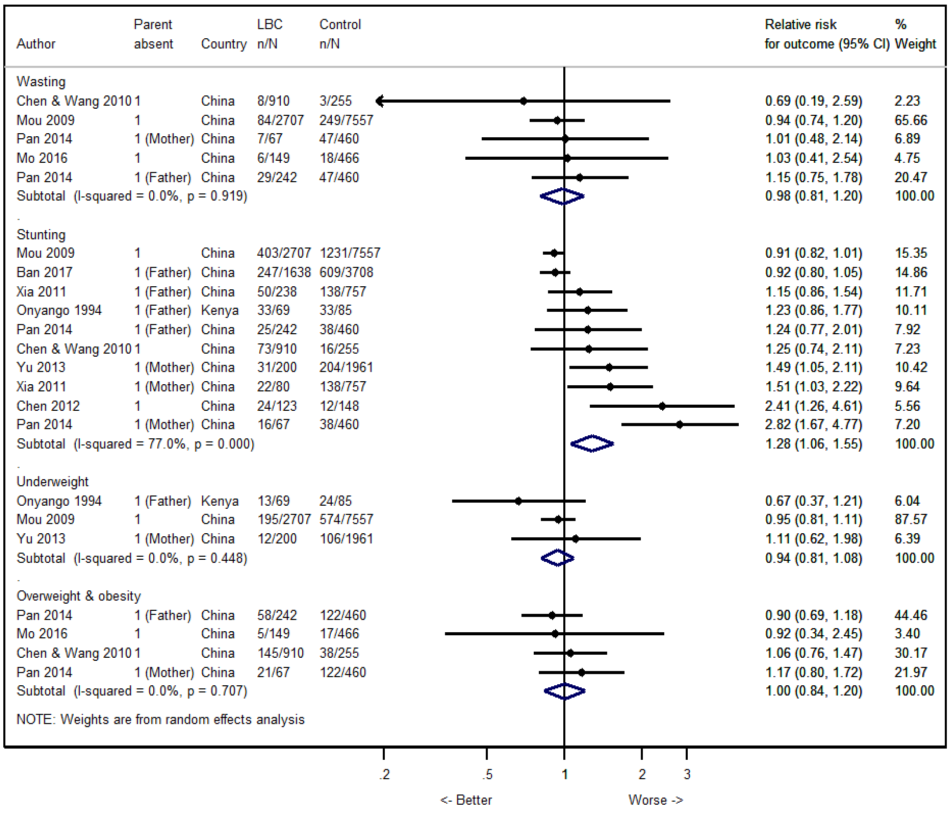

One parent absent – nutrition (continuous outcomes)

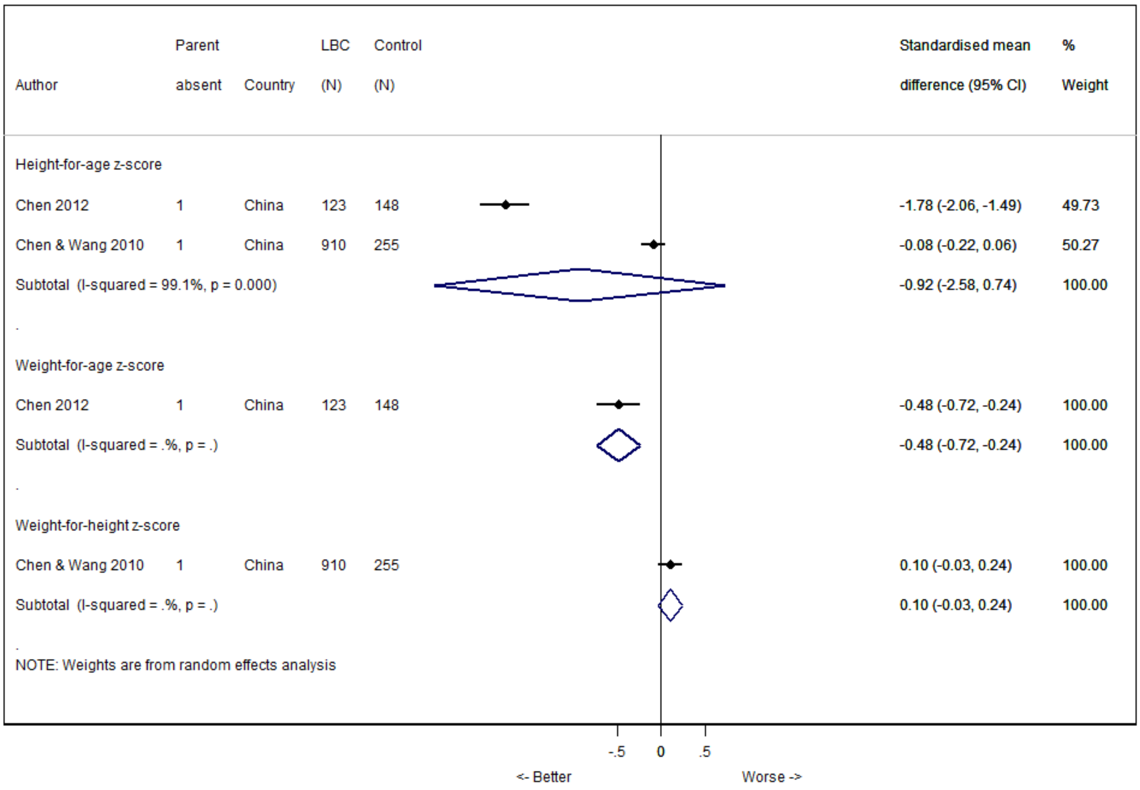

One parent absent – other outcomes (binary outcomes)

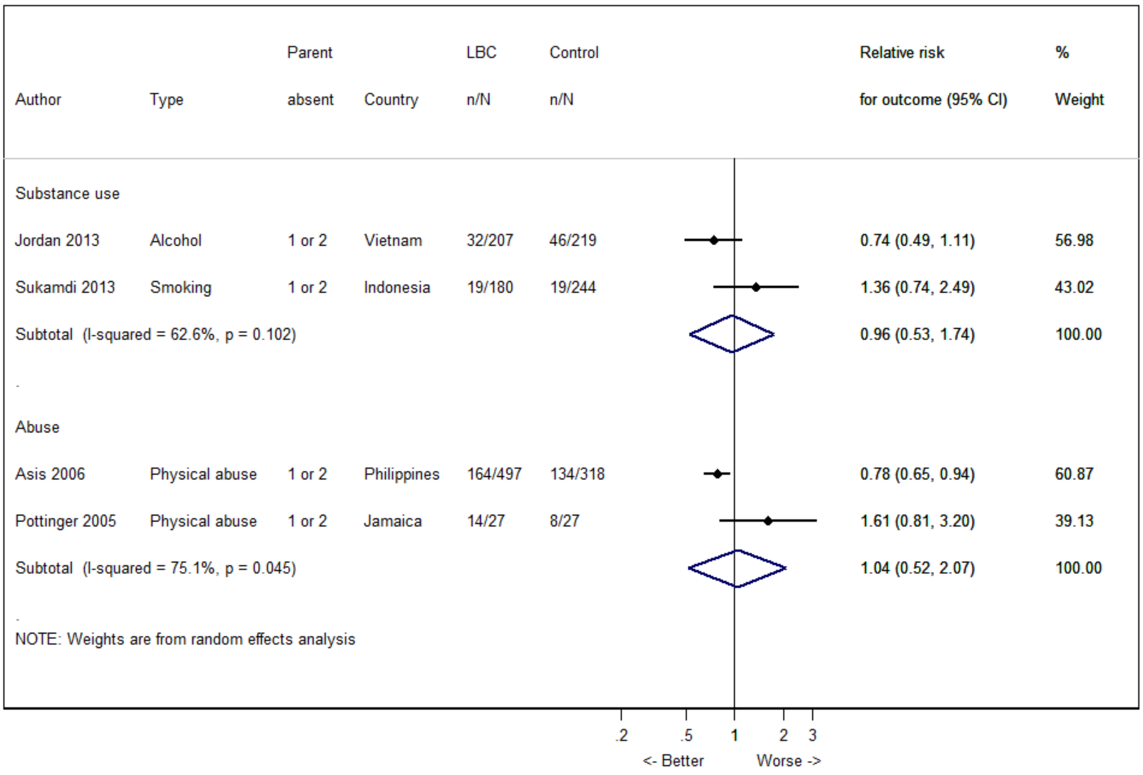

China – mental health (binary outcomes)

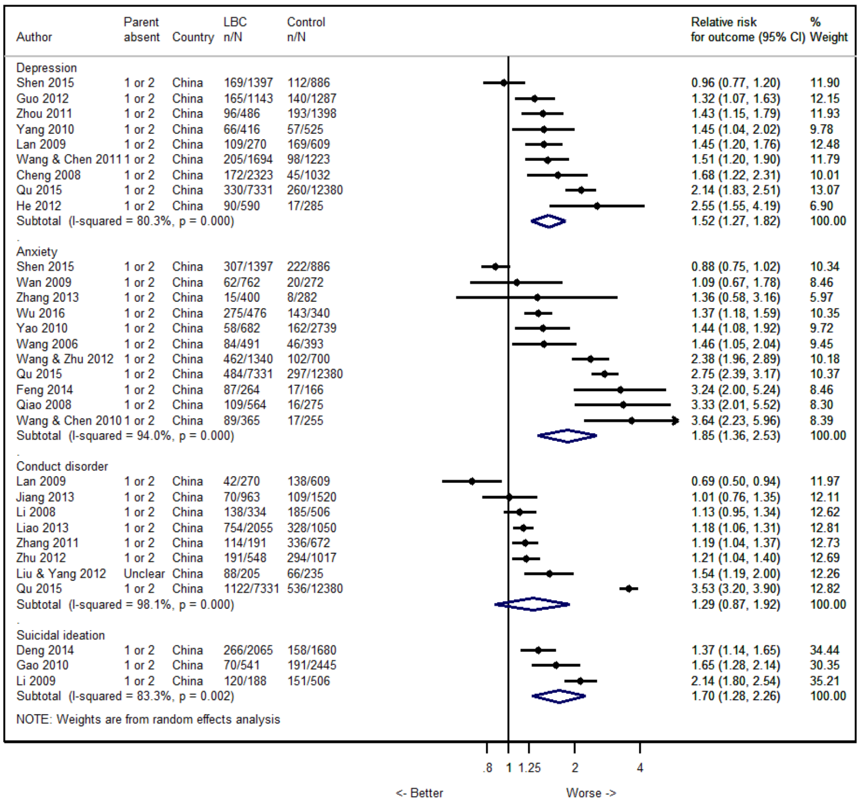

China – mental health (continuous outcomes)

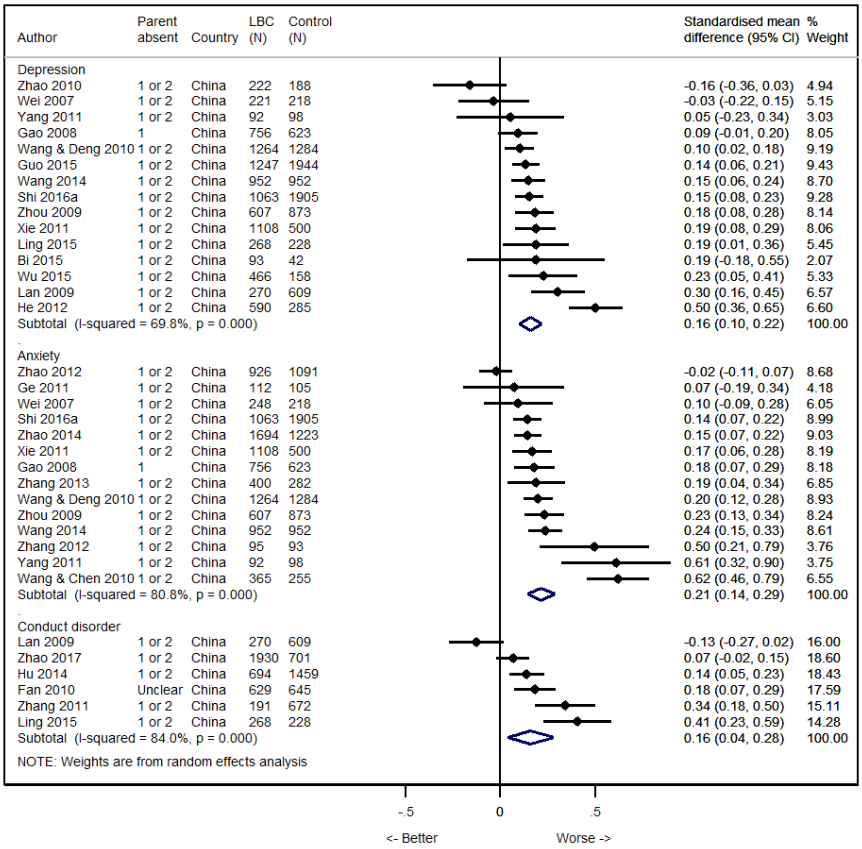

China – nutrition (binary outcomes)

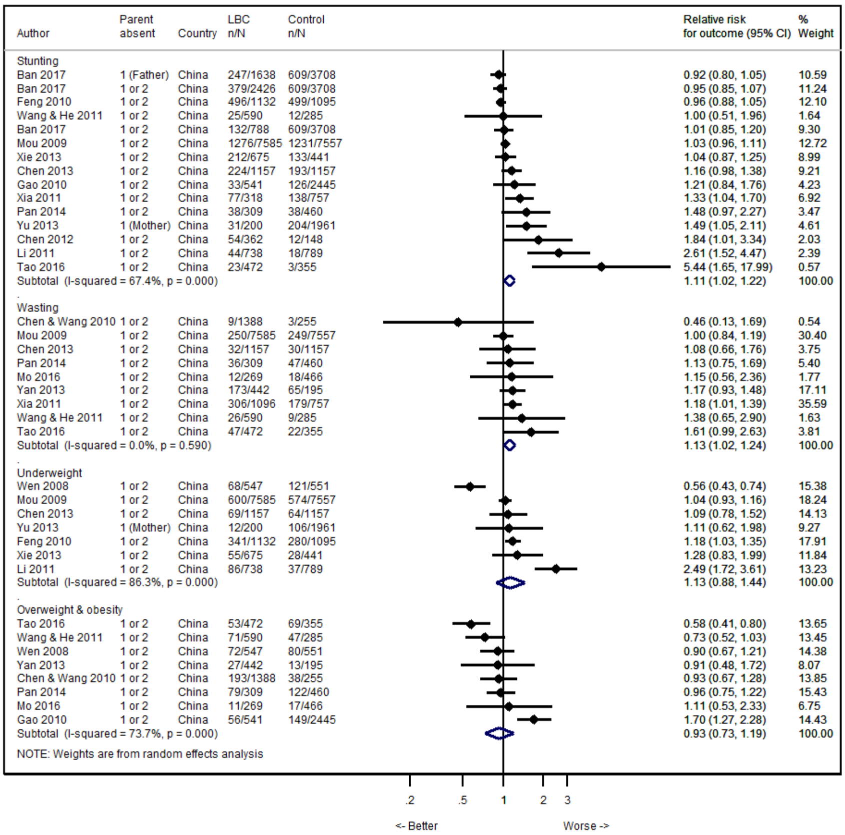

China – nutrition (continuous outcomes)

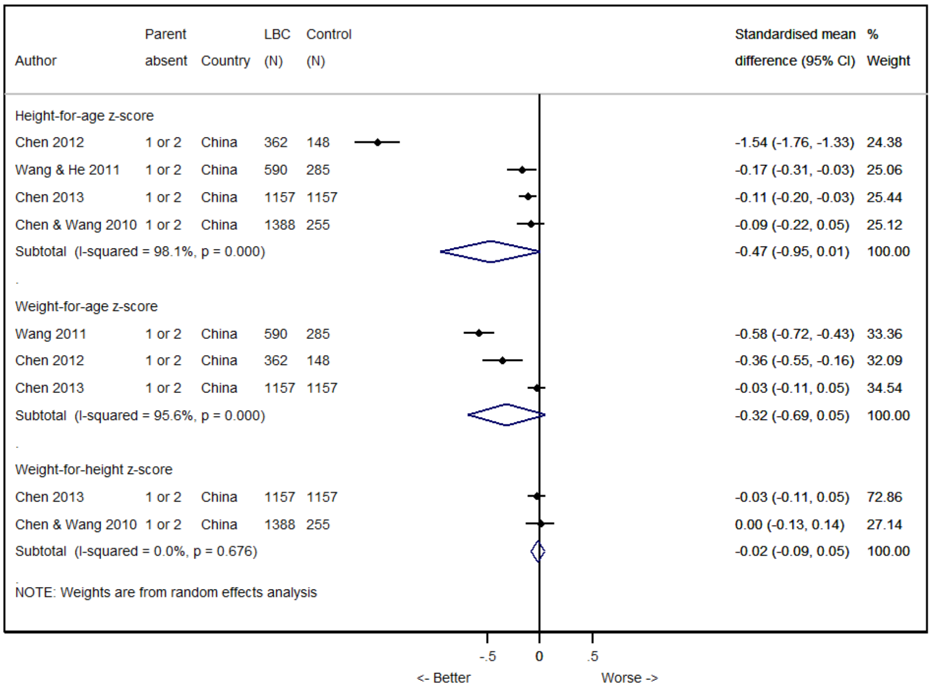

China – other outcomes (binary)

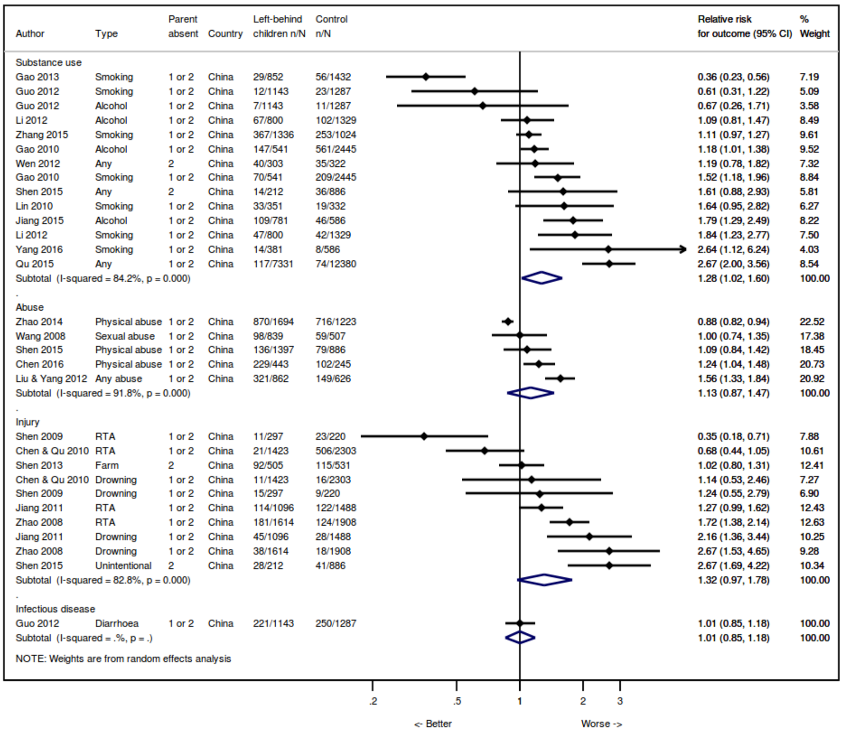

Rest of the world – mental health (binary outcomes)

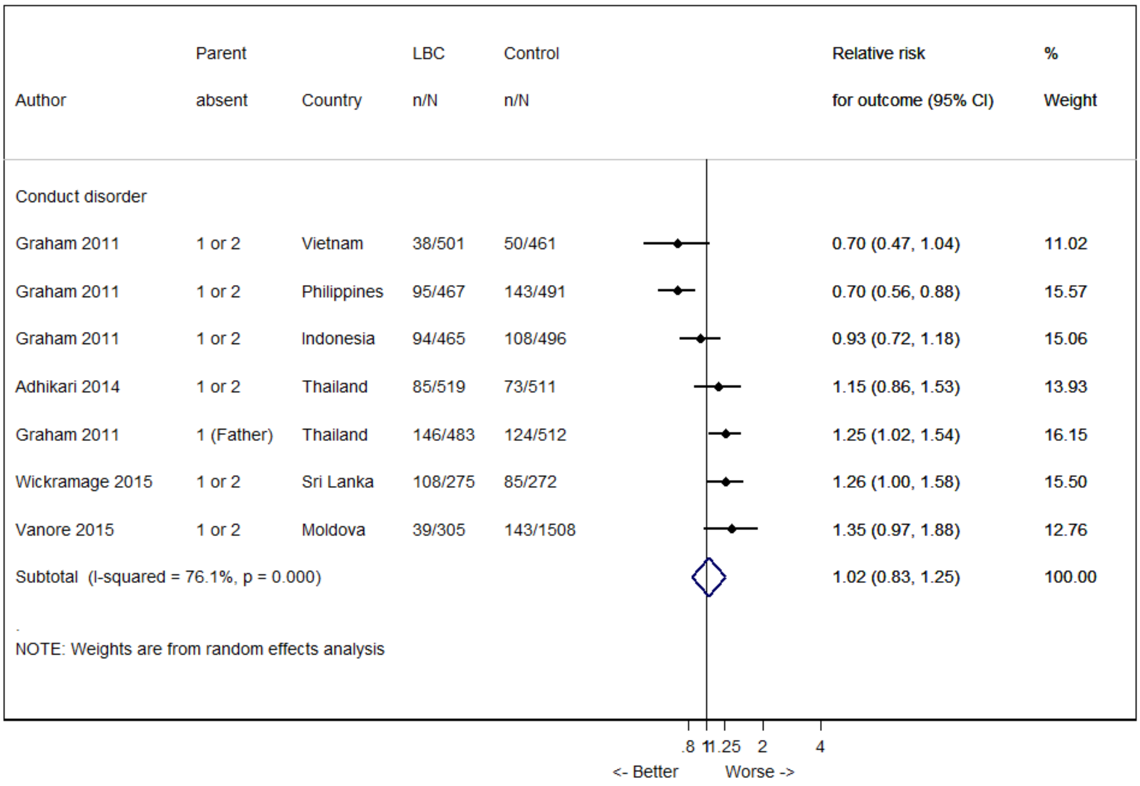

Rest of the world – mental health (continuous outcomes)

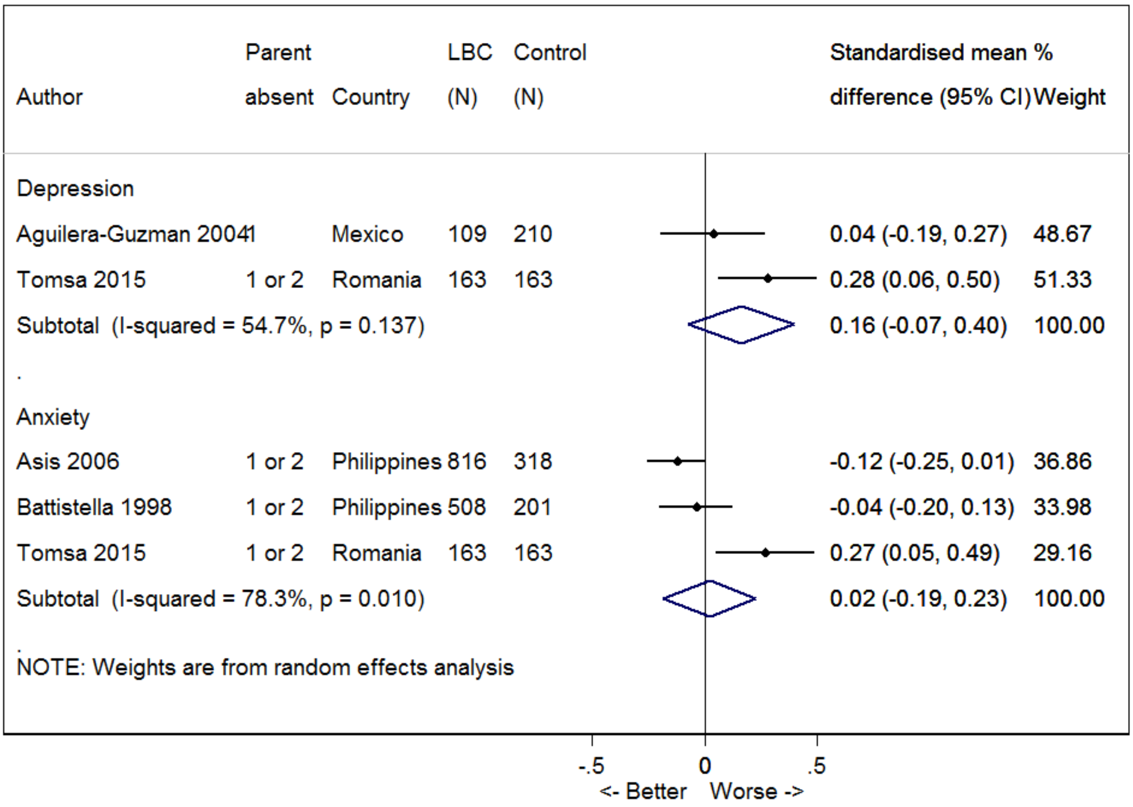

Rest of the world – nutrition (binary outcomes)

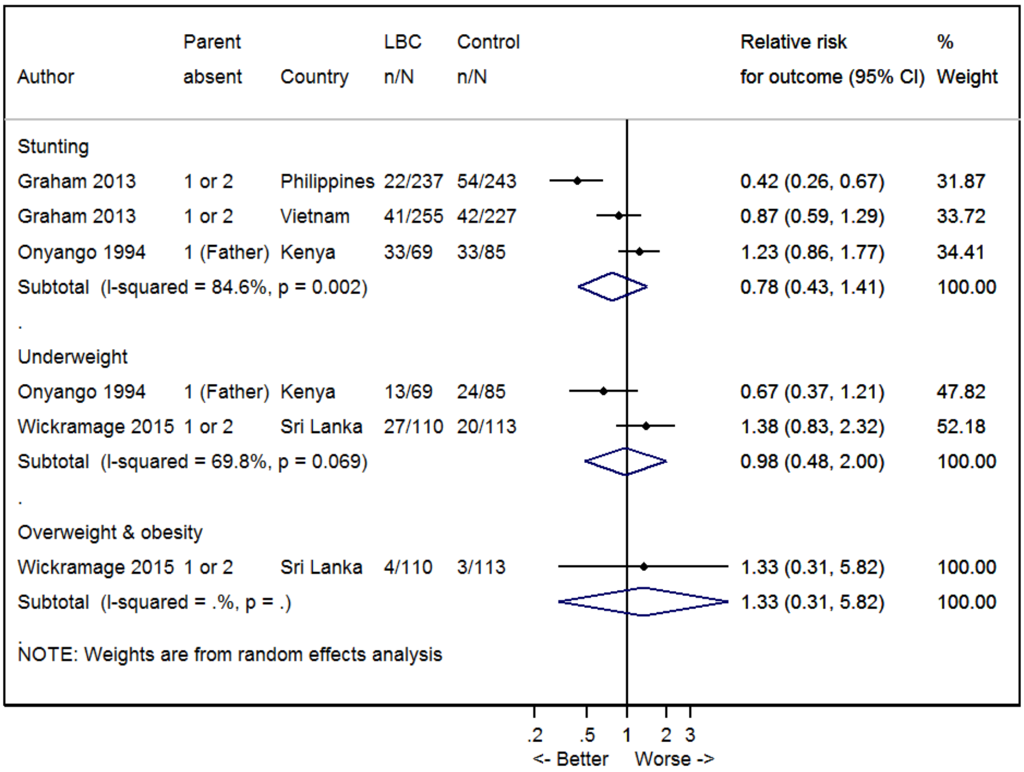

Rest of the world – other outcomes (binary outcomes)

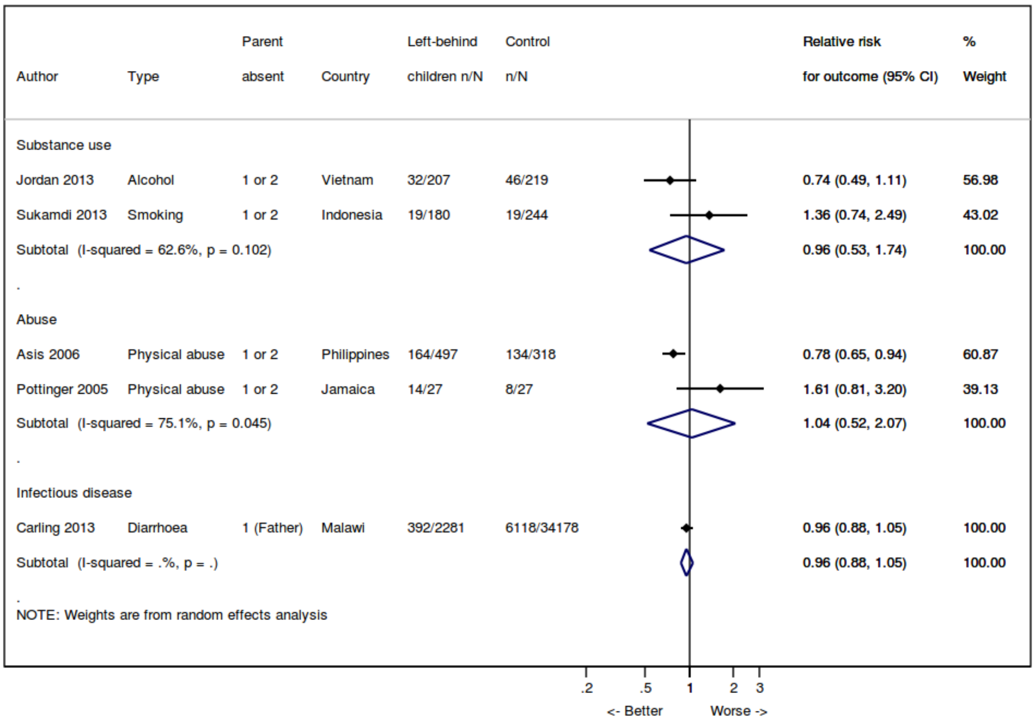

Sensitivity analysis

High quality studies – mental health (binary outcomes)

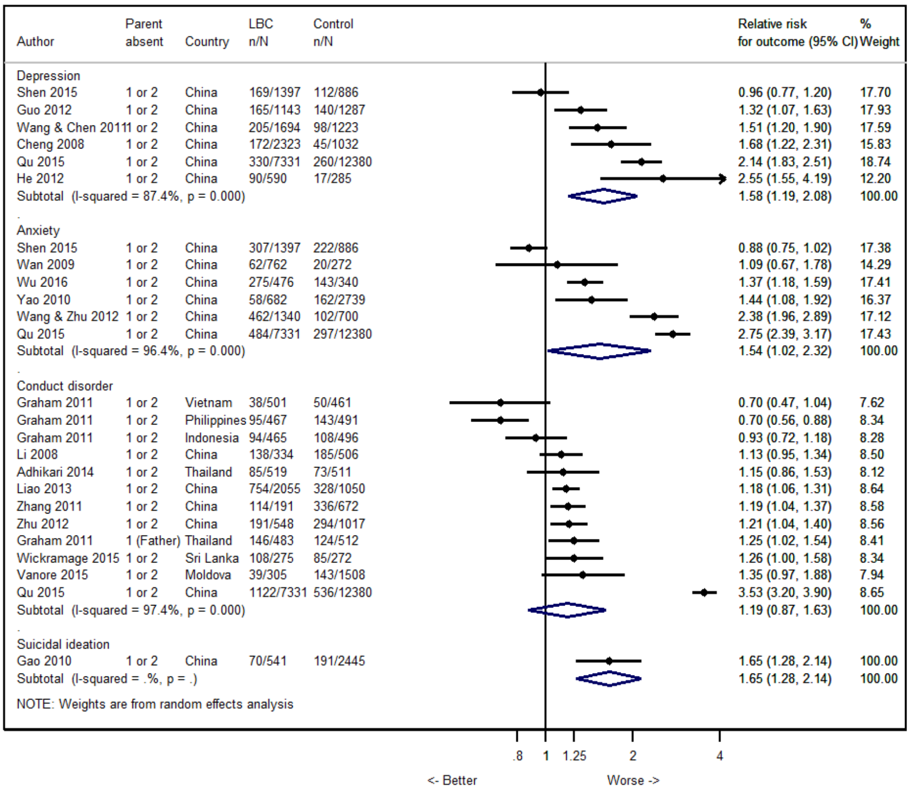

High quality studies – mental health (continuous outcomes)

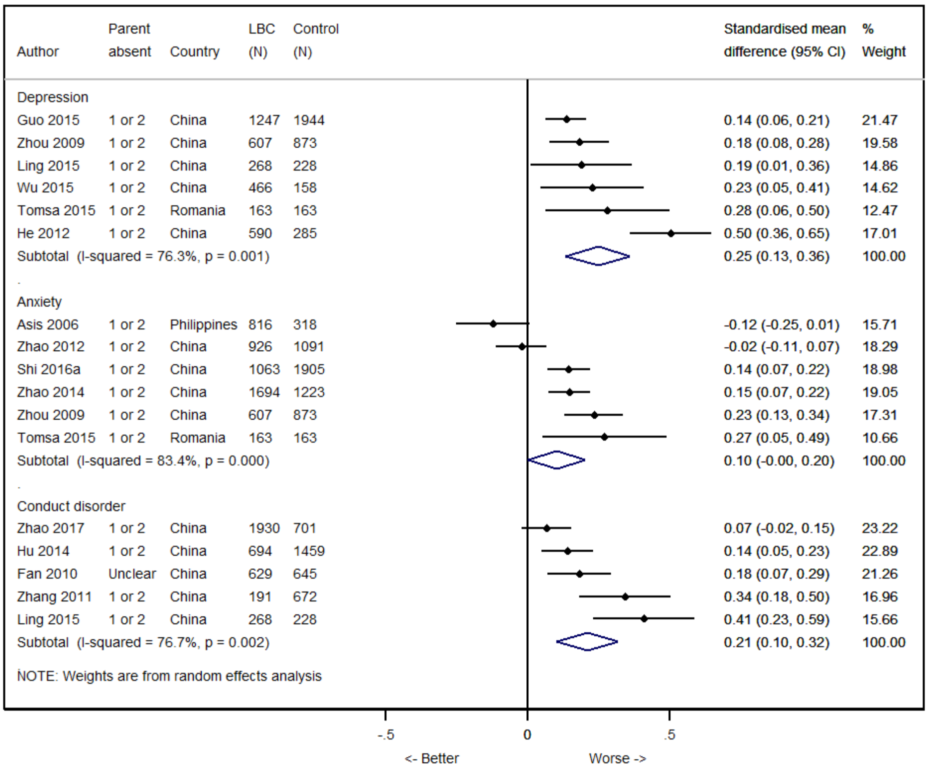

High quality studies – nutrition (binary outcomes)

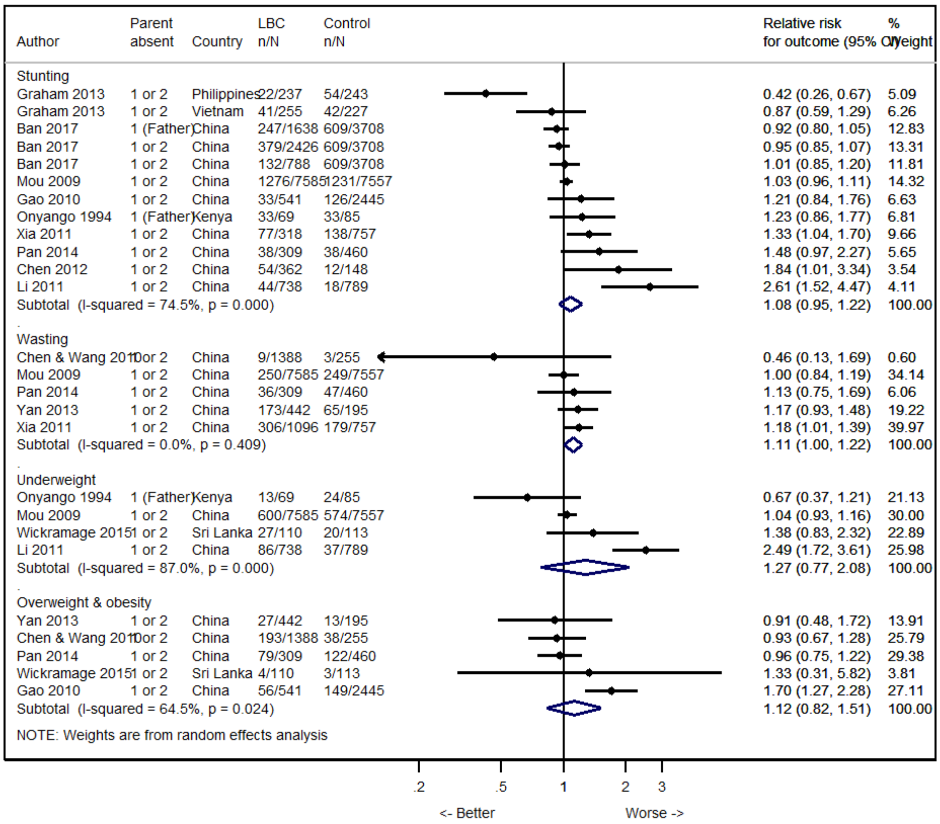

High quality studies – nutrition (continuous outcomes)

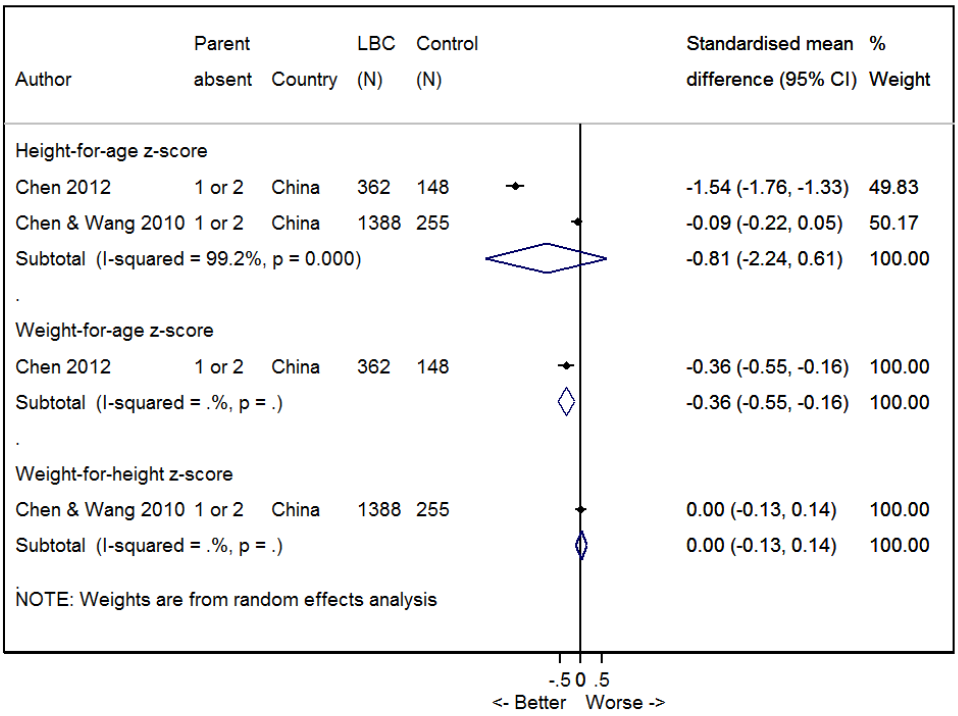

High quality – other outcomes (binary)

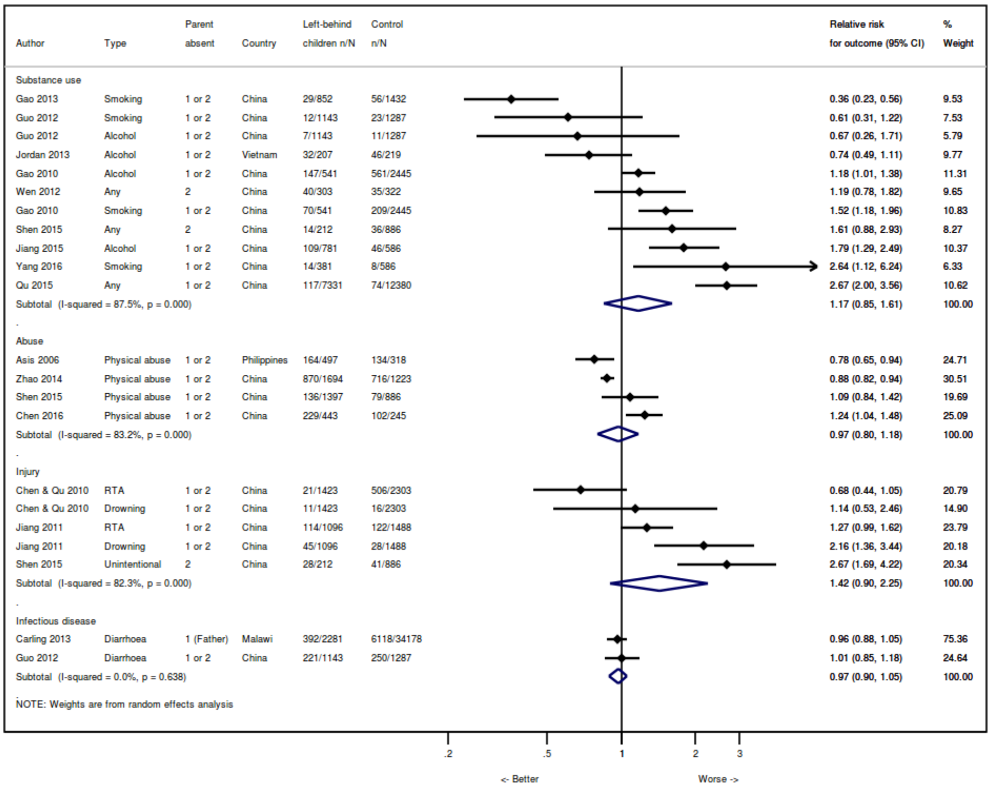

Mental health (binary outcomes), fixed effects

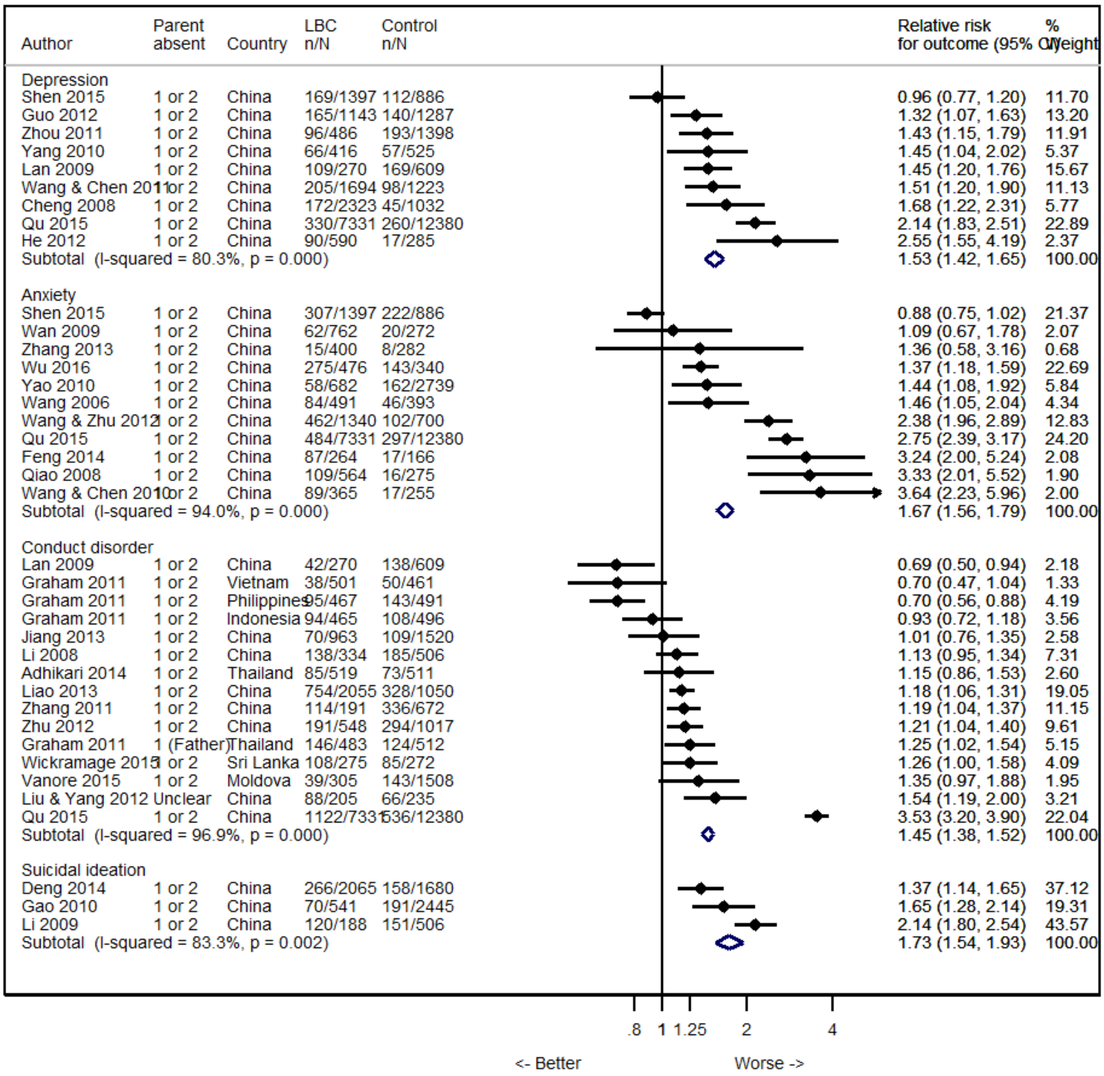

Mental health (continuous outcomes), fixed effects

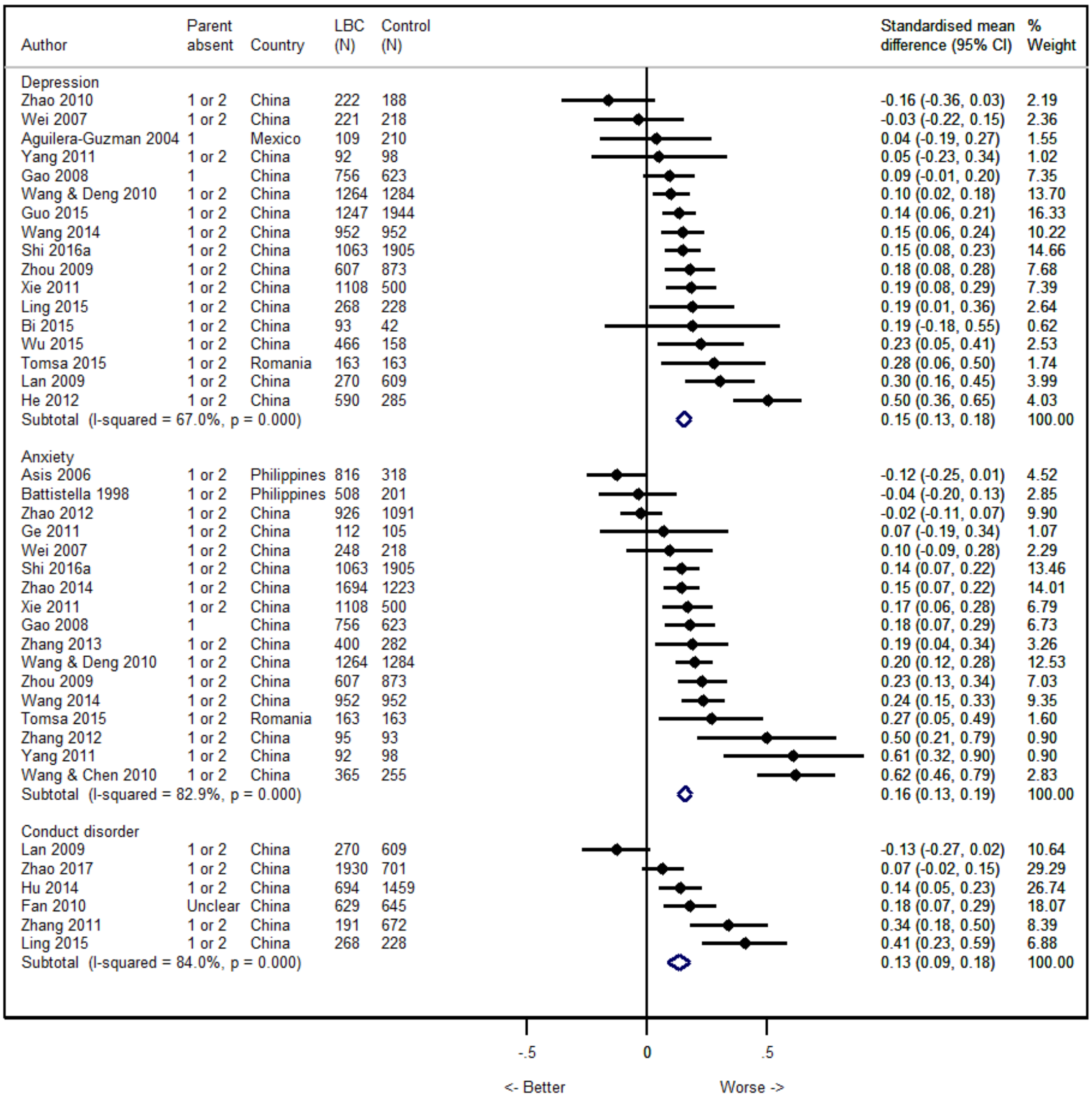

Nutrition (binary outcomes), fixed effects

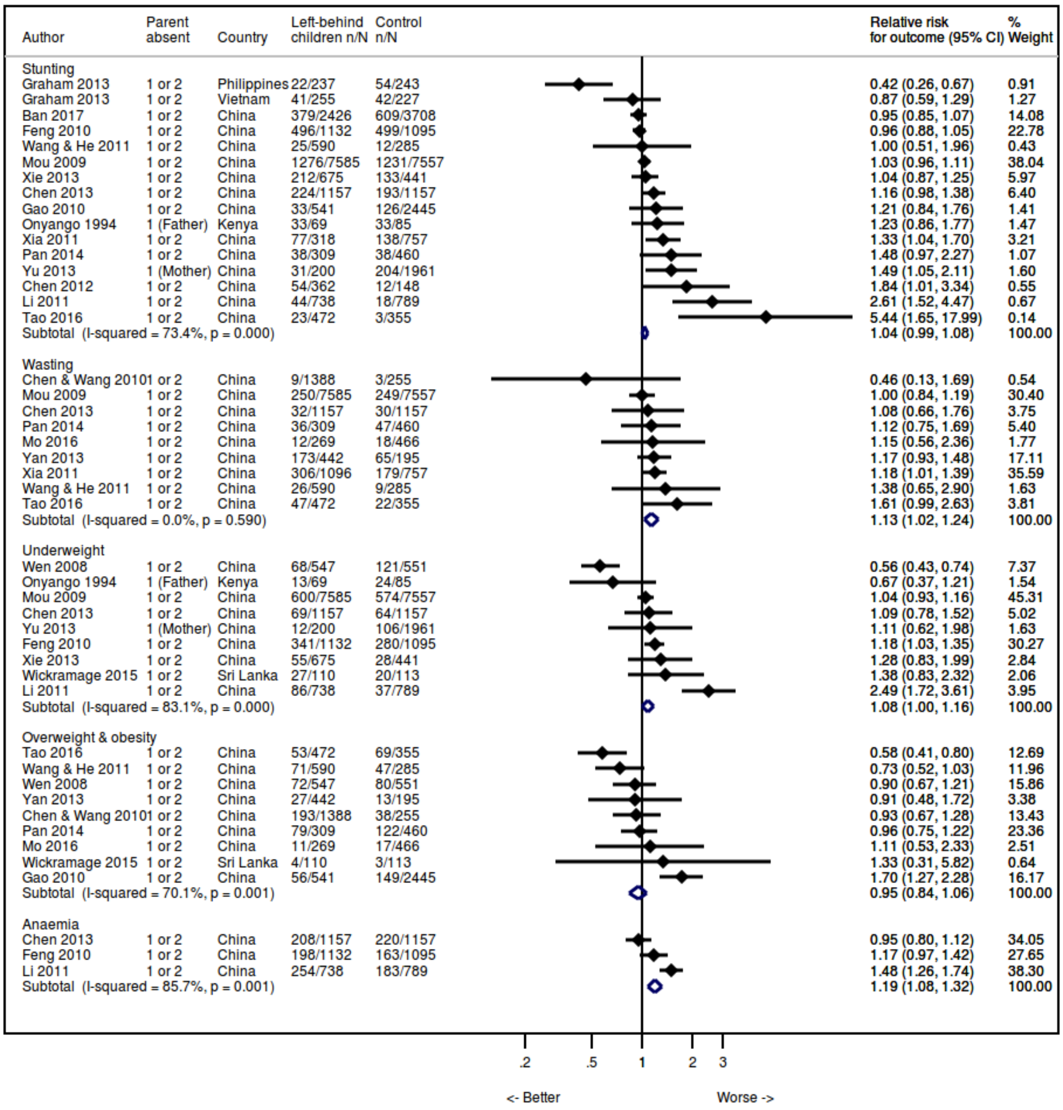
Author | Parent absent | Country | Left-behind children n/N | Control n/N | Relative risk for outcome (95% CI) | % Weight
Stunting
Graham 2013 1 or 2 Philippines 22/237 54/243 0.42 (0.26, 0.67) 0.91
Graham 2013 1 or 2 Vietnam 41/255 42/227 0.87 (0.59, 1.29) 1.27
Ban 2017 1 or 2 China 379/2426 609/3708 0.95 (0.85, 1.07) 14.08
Feng 2010 1 or 2 China 496/1132 499/1095 0.96 (0.88, 1.05) 22.78
Wang & He 2011 1 or 2 China 25/590 12/285 1.00 (0.51, 1.96) 0.43
Mou 2009 1 or 2 China 1276/7585 1231/7557 1.03 (0.96, 1.11) 38.04
Xie 2013 1 or 2 China 212/675 133/441 1.04 (0.87, 1.25) 5.97
Chen 2013 1 or 2 China 224/1157 193/1157 1.16 (0.98, 1.38) 6.40
Gao 2010 1 or 2 China 33/541 126/2445 1.21 (0.84, 1.76) 1.41
Onyango 1994 1 (Father) Kenya 33/69 33/85 1.23 (0.86, 1.77) 1.47
Xia 2011 1 or 2 China 77/318 138/757 1.33 (1.04, 1.70) 3.21
Pan 2014 1 or 2 China 38/309 38/460 1.48 (0.97, 2.27) 1.07
Yu 2013 1 (Mother) China 31/200 204/1961 1.49 (1.05, 2.11) 1.60
Chen 2012 1 or 2 China 54/362 12/148 1.84 (1.01, 3.34) 0.55
Li 2011 1 or 2 China 44/738 18/789 2.61 (1.52, 4.47) 0.67
Tao 2016 1 or 2 China 23/472 3/355 5.44 (1.65, 17.99) 0.14
Subtotal (I-squared = 73.4%, p = 0.000) 1.04 (0.99, 1.08) 100.00
Wasting
Chen & Wang 2010 1 or 2 China 9/1388 3/255 0.46 (0.13, 1.69) 0.54
Mou 2009 1 or 2 China 250/7585 249/7557 1.00 (0.84, 1.19) 30.40
Chen 2013 1 or 2 China 32/1157 30/1157 1.08 (0.66, 1.76) 3.75
Pan 2014 1 or 2 China 36/309 47/460 1.12 (0.75, 1.69) 5.40
Mo 2016 1 or 2 China 12/269 18/466 1.15 (0.56, 2.36) 1.77
Yan 2013 1 or 2 China 173/442 65/195 1.17 (0.93, 1.48) 17.11
Xia 2011 1 or 2 China 306/1096 179/757 1.18 (1.01, 1.39) 35.59
Wang & He 2011 1 or 2 China 26/590 9/285 1.38 (0.65, 2.90) 1.63
Tao 2016 1 or 2 China 47/472 22/355 1.61 (0.99, 2.63) 3.81
Subtotal (I-squared = 0.0%, p = 0.590) 1.13 (1.02, 1.24) 100.00
Underweight
Wen 2008 1 or 2 China 68/547 121/551 0.56 (0.43, 0.74) 7.37
Onyango 1994 1 (Father) Kenya 13/69 24/85 0.67 (0.37, 1.21) 1.54
Mou 2009 1 or 2 China 600/7585 574/7557 1.04 (0.93, 1.16) 45.31
Chen 2013 1 or 2 China 69/1157 64/1157 1.09 (0.78, 1.52) 5.02
Yu 2013 1 (Mother) China 12/200 106/1961 1.11 (0.62, 1.98) 1.63
Feng 2010 1 or 2 China 341/1132 280/1095 1.18 (1.03, 1.35) 30.27
Xie 2013 1 or 2 China 55/675 28/441 1.28 (0.83, 1.99) 2.84
Wickramage 2015 1 or 2 Sri Lanka 27/110 20/113 1.38 (0.83, 2.32) 2.06
Li 2011 1 or 2 China 86/738 37/789 2.49 (1.72, 3.61) 3.95
Subtotal (I-squared = 83.1%, p = 0.000) 1.08 (1.00, 1.16) 100.00
Overweight & obesity
Tao 2016 1 or 2 China 53/472 69/355 0.58 (0.41, 0.80) 12.69
Wang & He 2011 1 or 2 China 71/590 47/285 0.73 (0.52, 1.03) 11.96
Wen 2008 1 or 2 China 72/547 80/551 0.90 (0.67, 1.21) 15.86
Yan 2013 1 or 2 China 27/442 13/195 0.91 (0.48, 1.72) 3.38
Chen & Wang 2010 1 or 2 China 193/1388 38/255 0.93 (0.67, 1.28) 13.43
Pan 2014 1 or 2 China 79/309 122/460 0.96 (0.75, 1.22) 23.36
Mo 2016 1 or 2 China 11/269 17/466 1.11 (0.53, 2.33) 2.51
Wickramage 2015 1 or 2 Sri Lanka 4/110 3/113 1.33 (0.31, 5.82) 0.64
Gao 2010 1 or 2 China 56/541 149/2445 1.70 (1.27, 2.28) 16.17
Subtotal (I-squared = 70.1%, p = 0.001) 0.95 (0.84, 1.06) 100.00
Anaemia
Chen 2013 1 or 2 China 208/1157 220/1157 0.95 (0.80, 1.12) 34.05
Feng 2010 1 or 2 China 198/1132 163/1095 1.17 (0.97, 1.42) 27.65
Li 2011 1 or 2 China 254/738 183/789 1.48 (1.26, 1.74) 38.30
Subtotal (I-squared = 85.7%, p = 0.001) 1.19 (1.08, 1.32) 100.00
.2
.5
1
2
3
<- Better
Worse ->

Nutrition (continuous outcomes), fixed effects

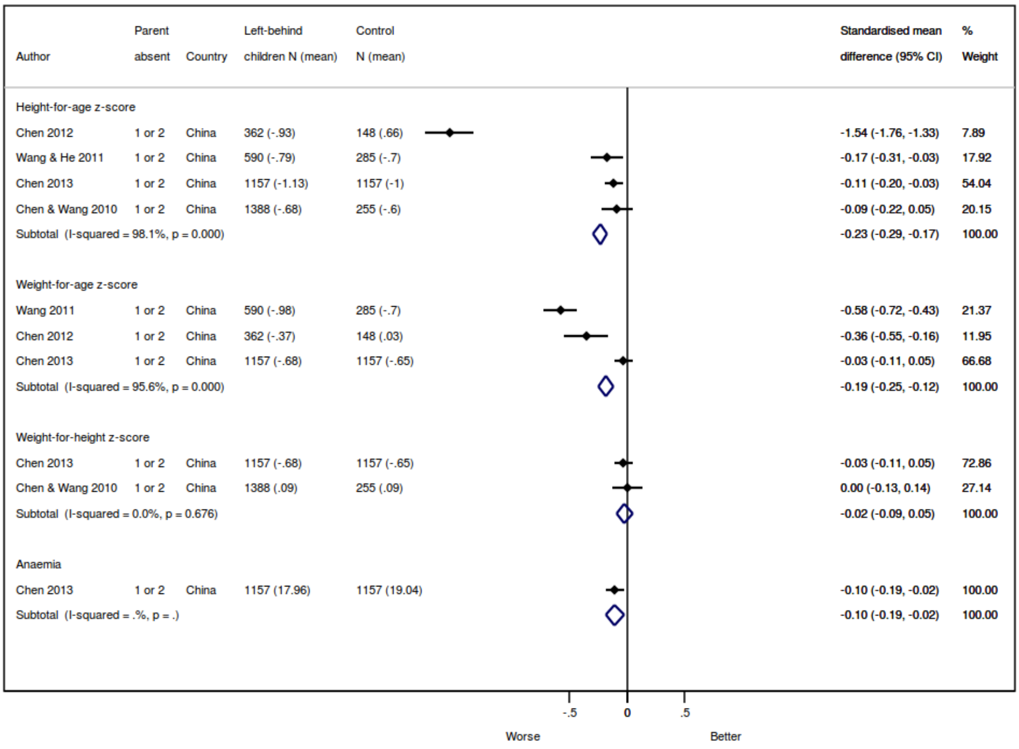

Other outcomes (binary), fixed effects

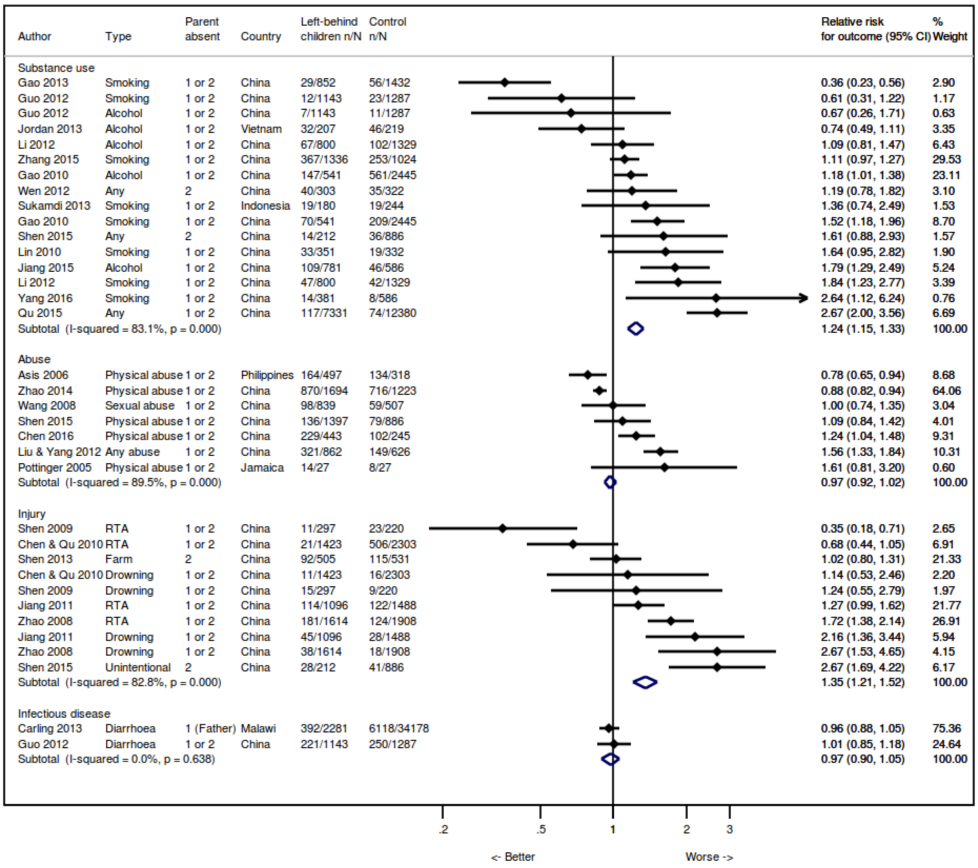
Author Type Parent absent Country Left-behind children n/N Control n/N Relative risk for outcome (95% CI) % Weight
Substance use
Gao 2013 Smoking 1 or 2 China 29/852 56/1432 0.36 (0.23, 0.56) 2.90
Guo 2012 Smoking 1 or 2 China 12/1143 23/1287 0.61 (0.31, 1.22) 1.17
Guo 2012 Alcohol 1 or 2 China 7/1143 11/1287 0.67 (0.26, 1.71) 0.63
Jordan 2013 Alcohol 1 or 2 Vietnam 32/207 46/219 0.74 (0.49, 1.11) 3.35
Li 2012 Alcohol 1 or 2 China 67/800 102/1329 1.09 (0.81, 1.47) 6.43
Zhang 2015 Smoking 1 or 2 China 367/1336 253/1024 1.11 (0.97, 1.27) 29.53
Gao 2010 Alcohol 1 or 2 China 147/541 561/2445 1.18 (1.01, 1.38) 23.11
Wen 2012 Any 2 China 40/303 35/322 1.19 (0.78, 1.82) 3.10
Sukamdi 2013 Smoking 1 or 2 Indonesia 19/180 19/244 1.36 (0.74, 2.49) 1.53
Gao 2010 Smoking 1 or 2 China 70/541 209/2445 1.52 (1.18, 1.96) 8.70
Shen 2015 Any 2 China 14/212 36/886 1.61 (0.88, 2.93) 1.57
Lin 2010 Smoking 1 or 2 China 33/351 19/332 1.64 (0.95, 2.82) 1.90
Jiang 2015 Alcohol 1 or 2 China 109/781 46/586 1.79 (1.29, 2.49) 5.24
Li 2012 Smoking 1 or 2 China 47/800 42/1329 1.84 (1.23, 2.77) 3.39
Yang 2016 Smoking 1 or 2 China 14/381 8/586 2.64 (1.12, 6.24) 0.76
Qu 2015 Any 1 or 2 China 117/7331 74/12380 2.67 (2.00, 3.56) 6.69
Subtotal (I-squared = 83.1%, p = 0.000) 1.24 (1.15, 1.33) 100.00
Abuse
Asis 2006 Physical abuse 1 or 2 Philippines 164/497 134/318 0.78 (0.65, 0.94) 8.68
Zhao 2014 Physical abuse 1 or 2 China 870/1694 716/1223 0.88 (0.82, 0.94) 64.06
Wang 2008 Sexual abuse 1 or 2 China 98/839 59/507 1.00 (0.74, 1.35) 3.04
Shen 2015 Physical abuse 1 or 2 China 136/1397 79/886 1.09 (0.84, 1.42) 4.01
Chen 2016 Physical abuse 1 or 2 China 229/443 102/245 1.24 (1.04, 1.48) 9.31
Liu & Yang 2012 Any abuse 1 or 2 China 321/862 149/626 1.56 (1.33, 1.84) 10.31
Pottinger 2005 Physical abuse 1 or 2 Jamaica 14/27 8/27 1.61 (0.81, 3.20) 0.60
Subtotal (I-squared = 89.5%, p = 0.000) 0.97 (0.92, 1.02) 100.00
Injury
Shen 2009 RTA 1 or 2 China 11/297 23/220 0.35 (0.18, 0.71) 2.65
Chen & Qu 2010 RTA 1 or 2 China 21/1423 506/2303 0.68 (0.44, 1.05) 6.91
Shen 2013 Farm 2 China 92/505 115/531 1.02 (0.80, 1.31) 21.33
Chen & Qu 2010 Drowning 1 or 2 China 11/1423 16/2303 1.14 (0.53, 2.46) 2.20
Shen 2009 Drowning 1 or 2 China 15/297 9/220 1.24 (0.55, 2.79) 1.97
Jiang 2011 RTA 1 or 2 China 114/1096 122/1488 1.27 (0.99, 1.62) 21.77
Zhao 2008 RTA 1 or 2 China 181/1614 124/1908 1.72 (1.38, 2.14) 26.91
Jiang 2011 Drowning 1 or 2 China 45/1096 28/1488 2.16 (1.36, 3.44) 5.94
Zhao 2008 Drowning 1 or 2 China 38/1614 18/1908 2.67 (1.53, 4.65) 4.15
Shen 2015 Unintentional 2 China 28/212 41/886 2.67 (1.69, 4.22) 6.17
Subtotal (I-squared = 82.8%, p = 0.000) 1.35 (1.21, 1.52) 100.00
Infectious disease
Carling 2013 Diarrhoea 1 (Father) Malawi 392/2281 6118/34178 0.96 (0.88, 1.05) 75.36
Guo 2012 Diarrhoea 1 or 2 China 221/1143 250/1287 1.01 (0.85, 1.18) 24.64
Subtotal (I-squared = 0.0%, p = 0.638) 0.97 (0.90, 1.05) 100.00
.2 .5 1 2 3
<- Better
Worse ->

Meta-regression

Outcome	Regression coefficient	95% CI	I^2	No. of studies
**Mental health (continuous)**				
Age	-0.01	-0.05-0.03	70.5%	20
Gender	0.01	-0.01-0.03		
**Mental health (binary)**				
Age	0.91	0.78-1.05	92.4%	13
Gender	1.03	0.97-1.09		
**Nutrition (continuous)**				
Age	--	--	97.0%	4
Gender	-0.32	-1.23-0.59		
**Nutrition (binary)**				
Age	1.06	0.98-1.16	63.6%	14
Gender	1.08	0.97-1.21		
**Other outcomes**				
Age	1.42	0.82-2.44	79.7%	9
Gender	1.02	0.92-1.15		

References (same numbering as the main text)

9. Ban L, Guo S, Scherpbier RW, Wang X, Zhou H, Tata LJ. Child feeding and stunting prevalence in left-behind children: a descriptive analysis of data from a central and western Chinese population. *International journal of public health* 2017; **62**(1): 143-51.

10. Wen M, Lin D. Child development in rural China: children left behind by their migrant parents and children of nonmigrant families. *Child development* 2012; **83**(1): 120-36.

13. Gao Y, Li LP, Kim JH, Congdon N, Lau J, Griffiths S. The impact of parental migration on health status and health behaviours among left behind adolescent school children in China. *BMC public health* 2010; **10**: 56

21 Adhikari R, Jampaklay A, Chamratrithirong A, Richter K, Pattaravanich U, Vapattanawong P. The impact of parental migration on the mental health of children left behind. *J Immigr Minor Health* 2014; **16:** 781–89.

22 Aguilera-Guzman RM, Garcia MS, Garcia FJ. Psychometric characteristics of CES-D in a sample of Mexican rural adolescents in areas with a strong migratory tradition. *Salud Mental* 2004; **27:** 57–66.

23 Asis MM, Ruiz-Marave C. Leaving a legacy: parental migration and school outcomes among young children in the Philippines. *Asian Pac Migr J* 2013; **22:** 349–76.

24 Battistella G, Conaco CG. The impact of labour migration on the children left behind: a study of elementary school children in the Philippines. *SOJOURN* 1998; **13:** 220–41.

25 Bi C, Oyserman D. Left behind or moving forward? Effects of possible selves and strategies to attain them among rural Chinese children. *J Adolesc* 2015; **44:** 245–58.

26 Chen M, Chan KL. Parental absence, child victimization, and psychological well-being in rural China. *Child Abuse Negl* 2016; **59:** 45–54.

27 Cheng SG, Hao JH, Tao FB. Mental health status of left-behind adolescents in rural areas in Anhui. *Zhongguo Xue Xiao Wei Sheng* 2008; **29:** 429–41 (in Chinese).

28 Deng ZX, Li GY. Relationship between suicidal ideation and parental out-migration for work among left-behind children aged 14 years or younger in Guizhou province. *Zhong Guo Gong Gong Wei Sheng* 2014; **30:** 1154–56 (in Chinese).

29 Fan F, Su L, Gill MK, Birmaher B. Emotional and behavioral problems of Chinese left-behind children: a preliminary study. *Soc Psychiatry Psychiatr Epidemiol* 2010; **45:** 655–64.

30 Feng L. Investigation and comparison of psychological and behavioral characteristics of rural left-behind children and non-left-behind children and the intervention countermeasures. *Zhong Guo Fu You Bao Jian* 2014; **29:** 2027–29 (in Chinese).

31 Gao Y. A comparative study on mental development characteristics between non-parent-absent children and parent-absent children in different care-taking modes. *Zhong Guo Te Shu Jiao Yu* 2008; **97:** 56–61 (in Chinese).

32 Ge J, Luo S. The study of rural left-behind children's psychological anxiety. *Xian Dai Jiao Yu Ke Xue* 2011; **1:** 72–74 (in Chinese).

33 Graham E, Jordan LP. Migrant parents and the psychological well-being of left-behind children in southeast Asia. *J Marriage Fam* 2011; **73:** 763–87.

34 Guo J, Chen L, Wang X, et al. The relationship between internet addiction and depression among migrant children and left-behind children in China. *Cyberpsychol Behav Soc Netw* 2012; **15:** 585–90.

35 Guo J, Ren X, Wang X, et al. Depression among migrant and left-behind children in China in relation to the quality of parent-child and teacher-child relationships. *PLoS One* 2015; **10:** e0145606.

36 He B, Fan J, Liu N, et al. Depression risk of 'left-behind children' in rural China. *Psychiatry Res* 2012; **200:** 306–12.

37 Hou K, Liu Y, Qu Z, Jiang S. The social adjustment of left-behind children in rural China: a propensity score analysis. *Xin Li Fa Zhan Yu Jiao Yu* 2014; **30:** 646–55 (in Chinese).

38 Hu HW, Lu S, Huang CC. The psychological and behavioral outcomes of migrant and left-behind children in China. *Child Youth Serv Rev* 2014; **46:** 1–10.

39 Jiang Q. Emotional and behavioral problems among left-behind middle school children in Fujian province. *Zhong Guo Gong Gong Wei Sheng* 2013; **12:** 015 (in Chinese).

40 Jin YL, Wu XJ, Huang SP, Zhao HS, Sun GX, Xu JC. Comparative analysis on the mental health status of rural left-behind children in northern Jiangsu province. *Zhongguo xue xiao wei sheng* 2009; **30:** 472–73 (in Chinese).

41 Jones A, Sharpe J, Sogren M. Children's experiences of separation from parents as a consequence of migration. *CJSW* 2004; **3**: 89–109.

42 Lan YL, Li Y, Tan XJ, Zhang YY, Tan JY. Personality and emotion and behavior status of children left behind in rural areas. *Zhong Hua Xing Wei Yi Xue Yu Nao Ke Xue* 2009; **18:** 343–45 (in Chinese).

43 Li XY, Tang JL, Wu D, Xu WM, Yang L, Jin L. Study on behavioral problems among 5,973 middle-school students in an area. *Anhui Yi Ke Da Xue Xue Bao* 2008; **43:** 108–10 (in Chinese).

44 Li GY, Tao FB. Survey on mental health status and suicidal ideation among rural children left-behind. *Chin J Publ Health* 2009; **25:** 905–07.

45 Liao ZG, Chen ZH, Wang SH, Xi BR, Liu H, He SJ. Study and comparison of behavioral problems of left-behind children and non-left behind children in rural area of Jiangxi province. *Zhong Guo Fu You Bao Jian* 2013; **28:** 4501–3 (in Chinese).

46 Ling H, Fu E, Zhang JR. Effects of separation age and separation duration among left-behind children in China. *Soc Behav Pers* 2015; **43:** 241–54.

47 Liu XH, Yang YY, Ha LN, Wang X, Li Q, Dai X. Relationship between emotional problem behavior and social support in left-behind children. *Zhong Guo Quan Ke Yi Xue* 2012; **28:** 3287–90 (in Chinese).

48 Pottinger AM. Children's experience of loss by parental migration in inner-city Jamaica. *Am J Orthopsychiatry* 2005; **75:** 485–96.

49 Qiao L, Chen XN, Yuan P. Status of mental health of the left-behind children in certain regions of Sichuan. *Xian Dai Yu Fang Yi Xue* 2008; **16:** 3108–11 (in Chinese).

50 Qu Y, Jiang H, Zhang N, Wang D, Guo L. Prevalence of mental disorders in 6-16-year-old students in Sichuan province, China. *Int J Environ Res Public Health* 2015; **12:** 5090–107.

51 Ren Q, Treiman DJ. The consequences of parental labor migration in China for children's emotional wellbeing. *Soc Sci Res* 2016; **58:** 46–67.

52 Shen M, Gao J, Liang Z, Wang Y, Du Y, Stallones L. Parental migration patterns and risk of depression and anxiety disorder among rural children aged 10–18 years in China: a cross-sectional study. *BMJ Open* 2015; **5:** e007802.

53 Shi J, Chen Z, Yin F, Zhao J, Zhao X, Yao Y. Resilience as moderator of the relationship between left-behind experience and mental health of Chinese adolescents. *Int J Soc Psychiatry* 2016; **62:** 386–93.

54 Shi YJ, Bai Y, Shen YN, Kenny K, Rozelle S. Effects of parental migration on mental health of left-behind children: evidence from Northwestern China. *China World Econ* 2016; **24:** 105–22.

55 Sun XJ, Tian Y, Zhang YX, Xie XC, Heath MA, Zhou Z. Psychological development and educational problems of left-behind children in rural China. *SPI* 2015; **36:** 227–52.

56 Tao S, Yu L, Gao W, Xue W. Food preferences, personality and parental rearing styles: analysis of factors influencing health of left-behind children. *Qual Life Res* 2016; **25:** 2921–29.

57 Tomsa R, Jenaro C. Children left behind in romania: anxiety and predictor variables. *Psychol Rep* 2015; **116:** 485–512.

58 Vanore M, Mazzucato V, Siegel M. 'Left behind' but not left alone: parental migration & the psychosocial health of children in Moldova. *Soc Sci Med* 2015; **132:** 252–60.

59 Wan YM, Wang SF, Ma XH. Mental health condition of the left-behind children in rural area of Anhui province. *Zhongguo Xue Xiao Wei Sheng* 2009; **10:** 911–12 (in Chinese).

60 Wang XD, Chen X. A comparative study of social anxiety and cognitive deviation between home-staying and non-home-staying children. *Sichuan Shi Fan Da Xue Xue Bao* 2010; **37:** 57–61 (in Chinese).
61 Wang TZ, Chen MC, Sun YH, et al. Research on Children's depression and the influence of left-behind status in rural area. *Zhongguo xue xiao wei sheng* 2011; **32:** 1445–47 (in Chinese).
62 Wang SM, Deng DH, Zhou ZH. Mental health of the left-behind children in different types in rural Hanchuan. *Zhongguo xue xiao wei sheng* 2010; **6:** 005 (in Chinese).
63 Wang RX, Zhu LQ, Niu SX. Investigation on mental health of left-behind primary and secondary school students in northern remote rural of Hebei Province. *Jilin Da Xue Xue Bao* 2012; **6:** 065 (in Chinese).
64 Wang LF, Zhang S, Sun YH. Social anxiety and the influencing factors among pupils in rural area in Anhui. *Zhongguo xue xiao wei sheng* 2006; **10:** 014 (in Chinese).
65 Wang W, Zhao HW, Guo Y, Duan ZY. Study on the mental health status and the influencing factors of left-behind children in rural areas. *Xian Dai Yu Fang Yi Xue* 2014; **41:** 994–99 (in Chinese).
66 Wei J, Zheng H. Negative influence of the absence of parental rearing among middle school students left in rural areas. *Zhong Guo Xin Li Wei Sheng* 2007; **21:** 83 (in Chinese).
67 Wickramage K, Siriwardhana C, Vidanapathirana P, et al. Risk of mental health and nutritional problems for left-behind children of international labor migrants. *BMC Psychiatry* 2015; **15:** 39.
68 Wu Q, Lu D, Kang M. Social capital and the mental health of children in rural China with different experiences of parental migration. *Soc Sci Med* 2015; **132:** 270–77.
69 Wu YL, Zhao X, Li YF, et al. The risk and protective factors in the development of childhood social anxiety symptoms among Chinese children. *Psychiatry Res* 2016; **240:** 103–09.
70 Xie QH, Yang YP, Zhong L. Mental health status of rural left-behind children in Sinan county, Guizhou province. *Zhongguo xue xiao wei sheng* 2011; **32:** 468–69 (in Chinese).
71 Xu HW, Xie Y. The causal effects of rural-to-urban migration on children's well-being in China. *Eur Sociol Rev* 2015; **31:** 502–19.
72 Yang YJ, Tao FB, Wan YH. Depressive symptoms and the influencing factors among left-behind children. *Zhongguo xue xiao wei sheng* 2010; **3:** 321–23 (in Chinese).
73 Yang XM, Wu YW. A survey on the mental health status of rural left-behind children in Gansu. *Zhong Guo Fu You Bao Jian* 2011; **26:** 2165–67 (in Chinese).
74 Yao YS, Kang YW, Jin YL, et al. A prevalence survey on the mental health of left behind adolescent in Anhui province. *Zhong Hua Liu Xing Bing Xue* 2010; **31:** 1359–62 (in Chinese).
75 Zhang L, Luo XR, Li FF. Behavior problems in left-behind children living in rural areas of Changzhi City. *Zhong Hua Jian Kang Guan Li Xue* 2011; **5:** 50–53 (in Chinese).
76 Zhang JP. Analysis on the assessment results of family cohesion and adaptability of left-behind children. *Qian Yan*2012; **15:** 126–28 (in Chinese).
77 Zhang YL. Investigation on the mental health status of rural left-behind children. *Jiao Yu Xue Shu Yue Kan* 2013; **2013:** 83–90 (in Chinese).
78 Zhao JX, Liu X. Rural left-home-children's depression and antisocial behavior: the protective role of daily pleasures. *Xin Li Fa Zhan Yu Jiao Yu* 2010; **6:** 013 (in Chinese).
79 Zhao M, Li H, Li J, et al. Studying on the influence of migrant parents on mental health of their left-behind children in rural China. *Zhong Guo Wei Sheng Shi Ye Guan Li* 2012; **283:** 60–63 (In Chinese).
80 Zhao X, Chen J, Chen MC, Lv XL, Jiang YH, Sun YH. Left-behind children in rural China experience higher levels of anxiety and poorer living conditions. *Acta Paediatr* 2014; **103:** 665–70.
81 Zhao C, Wang F, Li L, Zhou X, Hesketh T. Long-term impacts of parental migration on Chinese children's psychosocial well-being: mitigating and exacerbating factors. *Soc Psychiatry Psychiatr Epidemiol* 2017; **52:** 669–77.

82 Zhou JY, Luo XR, Wei Z, et al. Characteristics of behavioral and emotional problems of left-behind children in rural area of Changsha. *Shi Yong Er Ke Lin Chuang Za Zhi* 2009; **24:** 1901–03 (in Chinese).
83 Zhou Z, Wang J. Analysis on the depression symptoms and associated factors in rural primary and middle school students. *Anhui Yi Ke Da Xue Xue Bao* 2011; **46:** 976–78 (in Chinese).
84 Zhu Y, Wang F, Li FH. Health condition of left-behind children in Dingxi county of Guizhou province. *Zhong Guo Gong Gong Wei Sheng* 2012; **4:** 045 (in Chinese).
85 Chen ZY. The health status of the left-behind children in rural China. *Zhong Guo Ren Kou Ke Xue* 2009; **5:** 011 (in Chinese).
86 Chen L, Wang XH, Qu ZY. An analysis of the growth and nutritional status of migrant children and left-at-home children. *Zhong Guo Te Shu Jiao Yu* 2010; **122:** 48–54 (in Chinese).
87 Chen C, He W, Wang Y, Deng L, Jia F. Nutritional status of children during and post-global economic crisis in China. *Biomed Environ Sci* 2011; **24:** 321–28.
88 Chen JY, Zhou H, Cao X, Zhou HY, Zhang S, Ma Y. Analysis on growth status of children in Ziyang, Sichuan province. *Zhongguo xue xiao wei sheng* 2012; **33:** 236–37 (in Chinese).
89 Chen SH, Liao ZG, He SJ. Analysis on the nutritional status of unattended children under 7 years in rural area of Jiangxi province. *Zhong Guo Fu You Bao Jian* 2013; **28:** 68–70 (in Chinese).
90 Davis J, Brazil N. Migration, remittances and nutrition outcomes of left-behind children: a national-level quantitative assessment of Guatemala. *PLoS One* 2016; **11:** e0152089.
91 Feng H, Zhang Y, Xian Y, Li Y. Analysis on the physical growth and nutrition status children under 7 years old in Guizhou. *Zhong Guo Fu You Bao Jian* 2010; **25:** 2407–09 (in Chinese).
92 Frank R. International migration and infant health in Mexico. *J Immigr Health* 2005; **7:** 11–22.
93 Graham E, Jordan LP. Does having a migrant parent reduce the risk of undernutrition for children who stay behind in south-east Asia? *Asian Pac Migr J* 2013; **22:** 315–48.
94 Li CM, Yang N, Zhang JN. Investigation of physical development among left-behind children in rural areas in western Hunan province. *Zhong Guo Quan Ke Yi Xue* 2011; **9:** 026 (in Chinese).
95 Lu Y. Internal migration, international migration, and physical growth of left-behind children: a study of two settings. *Health Place* 2015; **36:** 118–26.
96 Mo X, Xu L, Luo H, Wang X, Zhang F, Gai Tobe R. Do different parenting patterns impact the health and physical growth of 'left-behind' preschool-aged children? A cross-sectional study in rural China. *Eur J Public Health* 2016; **26:** 18–23.
97 Mou JS, Luo JY, Li YP, Shuai ZR, Liu XH. Study on the nutritional status and determinants among rural stranded children in China. *Zhonghua Liu Xing Bing Xue Za Zhi* 2009; **30:** 439–43 (in Chinese).
98 Mu R, de Brauw A. Migration and young child nutrition: evidence from rural China. *J Popul Econ* 2015; **28:** 631–57.
99 Nguyen CV. Does parental migration really benefit left-behind children? Comparative evidence from Ethiopia, India, Peru and Vietnam. *Soc Sci Med* 2016; **153:** 230–39.
100 Onyango A, Tucker K, Eisemon T. Household headship and child nutrition: a case study in western Kenya. *Soc Sci Med* 1994; **39:** 1633–39.
101 Pan CM, Chen XR. Growth and nutritional status among rural left-behind children. *Zhong Guo Gong Gong Wei Sheng* 2014; **6:** 004 (in Chinese).
102 Schmeer KK. Family structure and child anemia in Mexico. *Soc Sci Med* 2013; **95:** 16–23.
103 Wang YJ, He BY, Fang LH, Li HJ. Preliminary study on the health status among the" left-behind" children in the Xiantao rural area of Hubei Province. *Zhongguo dang dai er ke za zhi* 2011; **13:** 977–80 (in Chinese).
104 Wen YF, Wang JQ, Liu RQ. Study on the health condition of the 'left behind' children in rural areas of south Anhui province. *Xian Dai Yu Fang Yi Xue* 2008; **4:** 038 (in Chinese).
105 Xia YQ, Qing ZG, Su SH, Liu GH, Tang J. Physical growth and nutrition status among left-behind students in Guangdong. *Zhongguo xue xiao wei sheng* 2011; **32:** 1521–22 (in Chinese).

106 Xie Q, Long H, Shen XH, Tang WJ, Xie LX, Huang YC. Study on the food intake and medium and severe malnutrition in poor rural primary students in Guangxi. *Zhongguo Xue Xiao Wei Sheng* 2013; **34:** 979–80 (in Chinese).

107 Yan MH, Wang JS, Xin ZH. Investigation and research on physical health of the left-behind and the non left-behind children in north Jiangsu. *Anhui Nong Ye Ke Xue* 2013; **41:** 359–61 (in Chinese).

108 Yu D, Liu A, Yu W, et al. Malnutrition status and influencing factors in children with migrant worker mother in poor areas in China. *Wei sheng yan jiu* 2013; **42:** 429–32 (in Chinese).

109 Zhou C, Sylvia S, Zhang L, et al. China's left-behind children: impact of parental migration on health, nutrition, and educational outcomes. *Health Aff (Millwood)* 2015; **34:** 1964–71.

110 Gao Y, Li L, Chan EY, Lau J, Griffiths SM. Parental migration, self-efficacy and cigarette smoking among rural adolescents in south China. *PLoS One* 2013; **8:** e57569.

111 Jiang S, Chu J, Li C, et al. Alcohol consumption is higher among left-behind Chinese children whose parents leave rural areas to work. *Acta Paediatr* 2015; **104:** 1298–304.

112 Jin CC, Qu ZY, Wang XH. The characteristics and influence factors of smoking behavior of migrated children and unattended Children. *Zhong Guo Te Shu Jiao Yu* 2009; **113:** 70–74 (in Chinese).

113 Jordan LP, Graham E, Vinh ND. Alcohol Use among Very Early Adolescents in Vietnam: What Difference Does Parental Migration Make? *Asian Pac Migr J* 2013; **22:** 401–19.

114 Li GY, Luo TM, Tao FB. Effects of parental working out-of-home on health risk behavior and psychological status among left-behind children. *Zhong Guo Gong Gong Wei Sheng* 2012; **7:** 16–21 (in Chinese).

115 Lin D, Xu Y, Li X, Fan X. On the risk to and the future trend of the family environment and their relationship with rural children's smoking behavior. *Zhong Guo Te Shu Jiao Yu* 2011; **131:** 74–49 (in Chinese).

116 Sukamdi, Wattie AM. Tobacco use and exposure among children in migrant and non-migrant households in Java, Indonesia. *Asian Pac Migr J* 2013; **22:** 447–64.

117 Yang TT, Li C, Zhou C, et al. Parental migration and smoking behavior on left-behind children: evidence from a survey in rural Anhui, China. *Int J Equity Health* 2016; **15:** 127.

118 Zhang F, Zhang T, Xiong J, Zhang L, Chen J. Analysis on prevalence and influence factors of smoking and drinking among middle school students of Tujia and Miao in Xiangxi Area. *Wei Sheng Yan Jiu* 2015; **44:** 750–55 (in Chinese).

119 Carling J, Tonnessen M. Fathers' whereabouts and children's welfare in Malawi. *Dev South Afr* 2013; **30:** 724–42.

120 Schmeer K. Father absence due to migration and child illness in rural Mexico. *Soc Sci Med* 2009; **69:** 1281–86.

121 Wang Z, Zhang JY, Chen J, Tan L, Fan H, Gan HP. Analysis the prevalence status and influence factors of diarrhea among children under 5 years in rural areas of Sichuan province. *Xian Dai Yu Fang Yi Xue* 2012; **39:** 4069–75 (in Chinese).

122 Liu CY, Zhong ZH, Pan JP, et al. Analysis on the current situation of neglected rural children aged 0–6 years and its impact factors in the western areas of China. *Zhong Hua Liu Xing Bing Xue* 2012; **33:** 145–49 (in Chinese).

123 Wang JX. Investigation on safe sex issues among adolescent left-behind children. *Qing Nian Yan Jiu* 2008; **2008:** 7–14 (in Chinese).

124 Chen L, Qu ZY. Character and impact factors of injuries among left-behind children and migrant children. *Zhongguo xue xiao wei sheng* 2010; **31:** 332–34 (in Chinese).

125 Jiang W, Huang H, Wei J, Shi H, Zhu W, Zhang D. Epidemiological investigation on unintentional injuries of left-behind children in rural area of Nanning. *Zhong Guo Fu You Bao Jian* 2011; **26:** 563–65 (in Chinese).

126 Shen M, Yang S, Han J, et al. Non-fatal injury rates among the "left-behind children" of rural China. *Inj Prev* 2009; **15:** 244–47.

127 Shen M, Wang Y, Yang S, Du Y, Xiang H, Stallones L. Agricultural exposures and farm-related injuries among adolescents in rural China. *Inj Prev* 2013; **19:** 214–17.

128 Zhao KF, Su H, Fang XH, et al. Study on the distribution and risk factors of injuries among home-stranded children in rural area of Anhui province. *Zhong Hua Liu Xing Bing Xue* 2008; **29:** 338–42 (in Chinese).
